# Ru–Cu Nanoheterostructures for Efficient Hydrogen Evolution Reaction in Alkaline Water Electrolyzers

**DOI:** 10.1021/jacs.3c06726

**Published:** 2023-09-25

**Authors:** Yong Zuo, Sebastiano Bellani, Gabriele Saleh, Michele Ferri, Dipak V. Shinde, Marilena Isabella Zappia, Joka Buha, Rosaria Brescia, Mirko Prato, Roberta Pascazio, Abinaya Annamalai, Danilo Oliveira de Souza, Luca De Trizio, Ivan Infante, Francesco Bonaccorso, Liberato Manna

**Affiliations:** † Nanochemistry Department, Istituto Italiano di Tecnologia, Via Morego 30, 16163 Genova, Italy; ‡ BeDimensional S.p.A., Via Lungotorrente Secca, 30R, 16163 Genova, Italy; § Electron Microscopy Facility, Istituto Italiano di Tecnologia, Via Morego 30, 16163 Genova, Italy; ∥ Materials Characterization Facility, Istituto Italiano di Tecnologia, Via Morego 30, 16163 Genova, Italy; ⊥ Department of Chemistry and Industrial Chemistry, Università degli Studi di Genova, Via Dodecaneso 31, 16146 Genova, Italy; # ELETTRA Sincrotrone Trieste S.C.p.A., S.S. 14 Km 163.5, 34149 Trieste, Italy; ¶ BCMaterials, Basque Center for Materials, Applications, and Nanostructures, UPV/EHU, Science Park, Leioa 48940, Spain; ∇ Ikerbasque, Basque Foundation for Science, Bilbao 48009, Spain; ○ Graphene Laboratories, Istituto Italiano di Tecnologia, Via Morego 30, 16163 Genova, Italy

## Abstract

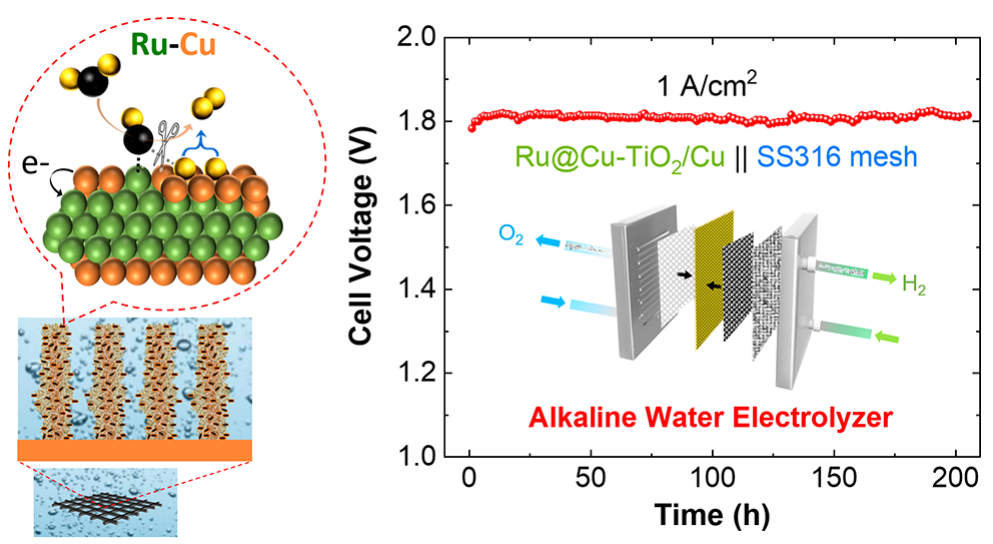

Combining multiple species working in tandem for different hydrogen evolution reaction (HER) steps is an effective strategy to design HER electrocatalysts. Here, we engineered a hierarchical electrode for the HER composed of amorphous-TiO_2_/Cu nanorods (NRs) decorated with cost-effective Ru–Cu nanoheterostructures (Ru mass loading = 52 μg/cm^2^). Such an electrode exhibits a stable, over 250 h, low overpotential of 74 mV at −200 mA/cm^2^ for the HER in 1 M NaOH. The high activity of the electrode is attributed, by structural analysis, operando X-ray absorption spectroscopy, and first-principles simulations, to synergistic functionalities: (1) mechanically robust, vertically aligned Cu NRs with high electrical conductivity and porosity provide fast charge and gas transfer channels; (2) the Ru electronic structure, regulated by the size of Cu clusters at the surface, facilitates the water dissociation (Volmer step); (3) the Cu clusters grown atop Ru exhibit a close-to-zero Gibbs free energy of the hydrogen adsorption, promoting fast Heyrovsky/Tafel steps. An alkaline electrolyzer (AEL) coupling the proposed cathode and a stainless-steel anode can stably operate in both continuous (1 A/cm^2^ for over 200 h) and intermittent modes (accelerated stress tests). A techno-economic analysis predicts the minimal overall hydrogen production cost of US$2.12/kg in a 1 MW AEL plant of 30 year lifetime based on our AEL single cell, hitting the worldwide targets (US$2–2.5/kg_H_2__).

## Introduction

Hydrogen is considered as a sustainable energy alternative to fossil fuels for the future worldwide economy.^1^ Green H_2_, generated from water electrolysis driven by electricity from renewable energy sources (e.g., solar and wind), represents a potential competitive solution against “gray” H_2_ produced by steam methane reforming, during which CO and CO_2_ are greatly released.^2^ Nevertheless, apart from the cost of electricity, the production cost of green H_2_ strongly relies on the energy efficiency of the water electrolyzer. For market-leading alkaline electrolyzers (AELs), the energy efficiency is still low, leading to an overall H_2_ production cost of ca. US$5/kg_H_2__, much higher than that of the “gray” H_2_ (ca. US$2.5/kg_H_2__).^3^ To boost the economic competitiveness of green H_2,_ it is crucial to design a novel generation of viable and highly efficient electrocatalysts integrable in water electrolyzers, capable of continuously working at practical current densities, that is, in the range of 0.2–1 A/cm^2^ or even higher.^4^ Indeed, a water electrolyzer produces H_2_ via the water electrolysis, which consists of the hydrogen evolution reaction (HER), occurring at the cathode, and the oxygen evolution reaction (OER), taking place at the anode.^5,6^ Although the HER process is kinetically favored in acidic media, efficient and widely available catalysts for the OER (e.g., Co–Fe,^7^ Ni–Fe materials,^8^ and stainless steel^9^) have been found to work efficiently mainly in alkaline media.^8,10^ Consequently, the design of high-performance electrocatalysts for the HER in alkaline media is pivotal to developing next-generation AELs.

Despite advances in catalysts based on earthly abundant materials,^11,12^ Pt-group metal (PGM)-based ones still feature the highest HER activity,^13,14^ even in alkaline media.^15^ In particular, Pt deposited on mesoporous carbon (Pt/C) is still considered the benchmark catalyst for the HER, not only in acidic but also in alkaline electrolytes,^16^ allowing the realization of state-of-the-art AELs.^17,18^ According to the Sabatier principle,^19^ the nearly zero Gibbs free energy of the hydrogen adsorption on the Pt surface (Δ*G*_Pt–Had_) makes Pt an ideal catalyst for the HER.^20,21^ Nevertheless, the catalytic activity of bare Pt toward the HER in alkaline media decreases drastically by 2 orders of magnitude compared to that in acidic electrolytes.^22^ This observation indicates that other factors also affect the HER activity of an electrode in alkaline media,^22,23^ including: (1) catalyst–electrolyte interactions^24^ (e.g., coadsorption of alkali cations on the surface of the catalyst^25^); (2) the structural reorganization of water molecules on the catalyst surface;^26^ and most importantly, (3) the water dissociation energy barrier associated with the generation of adsorbed hydrogen (H*) and hydroxyl (HO*) species (Volmer step),^14^ possibly leading to an acid-like reaction environment around catalytic nanoparticles in multicomponent electrodes.^15^

In addition, the massive use of Pt in AELs may implicate cost-related issues. In this regard, Ru, being the least expensive PGM (ca. one-fifth of the Pt cost^27^), has been considered as a valuable catalyst for the alkaline HER due to the rapid water dissociation process on its surface.^28^ However, Ru-based catalysts face several drawbacks: (1) the strong metal–hydrogen (Ru–H) bond impedes an efficient H-desorption, slowing down the overall HER kinetics;^28,29^ (2) the strong Ru–OH binding energy leads to the poisoning of Ru active sites required for readsorption/dissociation of water.^28,30^ Therefore, additional insights are needed to clarify the origin of the HER activity of Ru-based catalysts and define robust guidelines for their design. To address the former drawback, researchers have been focusing on the development of single-atom Ru catalysts^31^ or Ru-based heterostructures^32^ to properly tailor the electronic structure of the catalytic species for an efficient HER. Also, foreign metals with weak hydrogen adsorption have been incorporated into the Ru matrix to counteract the strong hydrogen adsorption of the latter (e.g., RuAu^28^ and RuCo^29^). Regarding the second drawback, additional oxophilic components (e.g., SnO_2_^33^ and Cr^34^) have been combined with Ru to act as water dissociation centers that might free the Ru surface from OH*. Recent studies also evidenced that subnanometric Ru nanocrystals have an optimal water dissociation ability, even superior to that of Ru single atoms, accelerating the alkaline HER rates.^35^ Recently, we reported Ru-decorated Cu nanoplates for alkaline HER, using an expensive Ti substrate to support Cu nanoplate arrays. The high activity of the resulting electrode was attributed to their peculiar structure, in which Cu nanoplates provided a high surface area support for Ru nanocrystals and abundant Ru–Cu interfaces were created.^36,37^

To go beyond these advances, here, we rationally designed catalysts using Ru nanocrystals as the active phase for water dissociation, growing them on a TiO_2_-decorated three-dimensional (3D) array of vertically oriented Cu nanorods (NRs) grown on a Cu mesh (CM) substrate (electrode named Ru@Cu–TiO_2_/Cu). A thin TiO_2_ layer was introduced atop Cu NRs to maintain the 3D architecture during the synthesis processes, while promoting the Volmer step during HER operation.^38,39^ Contrary to our previous work, the use of Ti was drastically reduced by replacing the Ti substrate with CM. By optimizing electroreduction and electrodeposition steps, we created Ru–Cu nanoheterostructures atop TiO_2_/Cu NRs. The mechanistic insights into the HER on our Ru–Cu nanoheterostructures were corroborated by systematic structural analysis, operando X-ray absorption spectroscopy (XAS), and first-principles simulations, providing the guidelines for the design of efficient electrodes for alkaline HER. Importantly, differently from our previous works where Ru nanoparticles were grown onto Cu nanoplatelets, we now realized that, since the surface energy of Cu is lower compared to that of Ru,^40^ Cu clusters are favorably formed atop Ru nanocrystals. Also, the final catalytic performances depend on the size of the Cu clusters, and the functional role of single species in performing the various HER steps was supported through theoretical calculations.

Our optimized Ru@Cu–TiO_2_/Cu stably delivered a HER current density of −200 mA/cm^2^ (@η = 74 mV) for more than 250 h, or at −500 mA/cm^2^ (@η = 115 mV) for over 160 h. The obtained high performance allowed us to validate Ru@Cu–TiO_2_/Cu as a cathode in a lab-scale AEL, in which stacked stainless-steel meshes (SSMs) were chosen as a cost-effective anode. The as-produced AEL demonstrates excellent operating performance (e.g., being stable at 1 A/cm^2^ for over 200 h) with an estimated overall cost of US$2.12/kg_H_2__ in a 1 MW AEL plant with a 30 year lifetime.

## Results and Discussion

### Synthesis and Characterization of the Electrode Catalysts

**Figure 1:**
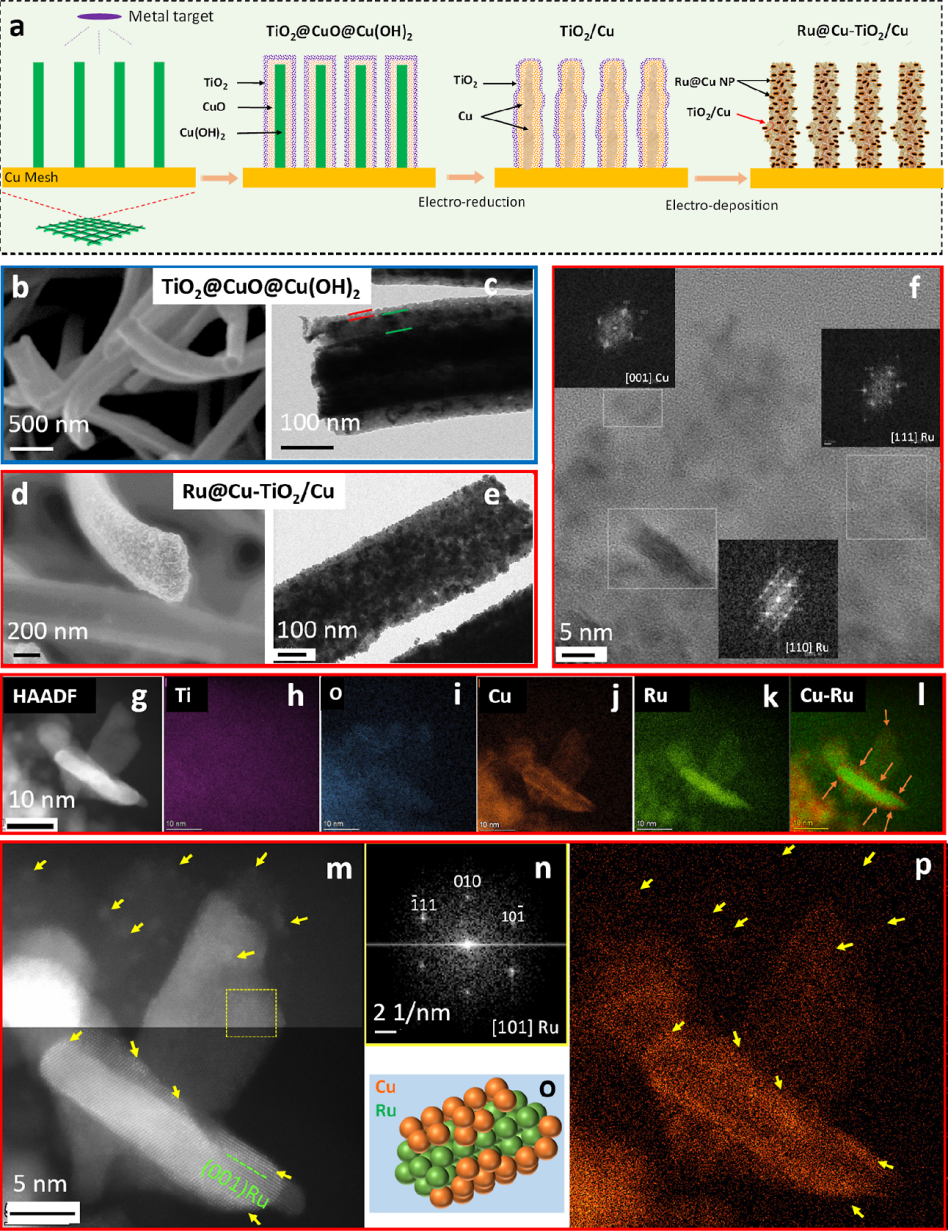
(a) Scheme of the in situ fabrication of the Ru@Cu–TiO_2_/Cu. Characterization of Ti@Cu@Cu(OH)_2_ and Ru@Cu–TiO_2_/Cu: (b) HRSEM and (c) TEM images of TiO_2_@CuO@Cu(OH)_2_ NRs grown on a Cu current collector (CM). The solid lines in (c) outline the different surface layers. (d) SEM and (e) TEM images of the Ru@Cu–TiO_2_/Cu sample. (f) HRTEM image of the Ru@Cu–TiO_2_/Cu surface showing Ru nanocrystals along with Cu nanocrystals. (g–l) HAADF STEM image and corresponding EDS elemental maps for Ti, O, Cu, and Ru acquired on a small surface region of the Ru@Cu–TiO_2_/Cu sample. The EDS maps confirm that the elongated features visible in STEM image are Ru-rich, thus correspond to the Ru nanocrystals identified by HRTEM, viewed side-on. These Ru nanocrystals are placed on top of the Cu-rich nanocrystalline core covered with some Ti and O, corresponding to the TiO_2_ layer. In panel (l), the arrows indicate additional concentrated Cu species, found on surface of one of the Ru nanocrystals, and distinct from the subsurface Cu core nanocrystals. (m) HRSTEM image revealing crystalline features of Ru nanocrystals, and (n) fast Fourier transform from one of these nanocrystals. Additional high contrast features are observed on surfaces of Ru nanocrystals and on other surfaces corresponding to the TiO_2_ layer (arrowed). According to the corresponding (p) Cu EDS map, such features are enriched in Cu only. Closer view of these clusters containing Cu atoms without any crystalline arrangement is presented in Figure S16. These observations indicate that Ru nanocrystals, as well as other surfaces, are covered by very small/thin Cu clusters, as depicted in the model structure in (o).

The preparation of the Ru@Cu–TiO_2_/Cu starts from the sequential sputtering of Cu and Ti layers onto the surface of a 3D array of vertically oriented Cu(OH)_2_ NRs grown on a CM substrate via a wet chemical approach^41^ (Figures 1a and S1 and Experimental Section). After the decoration of Cu(OH)_2_ NRs with sputtered Cu and Ti layers (Figure 1b), the latter spontaneously oxidize to CuO and TiO_2_ when exposed to environmental oxygen, leading to the sample named TiO_2_@CuO@Cu(OH)_2_. Then, Cu(OH)_2_ NRs and the CuO layer were electrochemically reduced to metallic Cu under a cathodic current in a 1 M NaOH electrolyte. Notably, such a treatment performed on bare Cu(OH)_2_ NRs (that is without sputtered Cu and Ti layers) led to a collapse of the NRs arrangement (Figure S2). The resulting sample, TiO_2_/Cu, was then decorated with Ru nanocrystals via electrodeposition employing K_2_RuCl_6_ solubilized in the electrolyte.

The entire fabrication protocol for Ru@Cu–TiO_2_/Cu was systematically optimized to retain the structural stability of the 3D NRs array while enhancing the overall HER activity (see details in Figures S3–S6). In this regard, the optimal thicknesses for Cu and Ti layers were found to be 60 and 30 nm, respectively. As regards the Ru electrodeposition, we systematically varied the applied potential, identifying −0.2 V_RHE_ as the optimal one. Meanwhile, the dosage of Ru precursor was systematically investigated (details in Figures S7–S9), finding that increasing the Ru concentration in the electrolyte accelerated the electrodeposition procedure and, at the same time, maximized the HER activity of the synthesized electrodes (Figure S9).

Figure 1b shows a high-resolution scanning electron microscopy (HRSEM) image of TiO_2_@CuO@Cu(OH)_2_ NRs. The transmission electron microscopy (TEM) images reveal that the TiO_2_@CuO@Cu(OH)_2_ NRs were composed of a relatively solid Cu(OH)_2_ core coated with two layers made of CuO and TiO_2_, respectively (Figures 1c, S10, and S11). Upon the electrochemical reduction step, the electrode was transformed into porous Cu NRs, mainly covered by a TiO_2_ surface layer (TiO_2_/Cu, Figure S12). After Ru electrodeposition, the electrode no longer exhibited a clear separation of various material layers (Figure 1e and S13). The native smooth surface of the NRs became rough (Figure 1d) and decorated with nanocrystals corresponding to Ru nanoplatelets protruding from the surface, recognized more easily when viewed side-on (Figure S14a). The mean diameter of the final NRs was ∼300 nm (Figure 1e), while their length was ∼15 μm (Figure S13d).

The high-resolution TEM (HRTEM) and high-angle annular dark field (HAADF) scanning TEM (STEM) observations with corresponding energy-dispersive X-ray spectroscopy (EDS) maps reveal that the surface of the NRs is composed of different species: (1) the Ru nanocrystals, protruding from the surface, with thickness of up to 5 nm and diameter of at least 20 nm; (2) TiO_2_ as well as (3) Cu nanocrystals exposed on the surface but originating from the core structure; and additionally (4) very fine Cu clusters present on all topmost surfaces, particularly on the Ru nanocrystals. These observations are summarized in Figures 1f–n, S14, and S15. The HAADF STEM image and the corresponding EDS maps in Figure S16 reveal the porous nature of the NRs with Cu and Ru associated with the characteristic elongated features corresponding to side-oriented Ru nanocrystals. The HRTEM image in Figure 1f shows one such side-oriented Ru nanocrystal in the [110] zone axis of the hcp structure (PDF card 01-071-3766), along with another Ru nanocrystal in [111] orientation and one of the Cu nanocrystals oriented favorably for its fcc crystal structure (PDF card 01-089-2838). The TiO_2_, also present on the surface between the Ru nanocrystals, was mostly amorphous. The Z-contrast in high resolution STEM (HRSTEM) images, combined with EDS mapping, further reveals very fine clusters and similar low-dimensional Cu structures on all surfaces, in some instances forming what appears as a patchy shell of Cu atoms on the surface of Ru nanocrystals (Figure 1m–p). Higher magnification HRSTEM images from Ru nanocrystals and the surrounding structures clearly reveal high contrast features of 1–2 nm in size and, according to EDS mapping, enriched in Cu only, not exhibiting any structural ordering characteristic of a crystal. The formation of such amorphous Cu clusters is attributed to the migration and dissolution-redeposition of Cu (Cu → CuOH → Cu) on Ru under negative potentials (i.e., −0.2 V vs RHE) in alkaline media.^42,43^ There was no evidence of Cu–Ru alloy formation, which can be explained considering that Cu and Ru are immiscible.^44,45^ The formation of Ru–Cu nanoheterostructures is consistent with the work of Chyan et al.,^46^ in which the electrodeposition of Cu onto Ru did not produce any alloyed structure even with annealing up to 800 °C. A thorough discussion on Ru–Cu bulk immiscibility and alloying at the nanoscale can be found in the Supporting Information (section “Discussion on Ru–Cu Miscibility and Possible Alloying”).

X-ray diffraction and X-ray photoelectron spectroscopy (XPS) analyses of Ru@Cu–TiO_2_/Cu (Figure S17) indicated the presence of metallic Cu, Cu_2_O, TiO_2_, and metallic Ru. The Cu XPS and Auger spectra revealed an electron transfer from Cu to Ru, while no relevant interaction between TiO_2_ and Ru was noticed from the Ti 2p spectrum. Inductively coupled plasma optical emission spectroscopy (ICP–OES) measurements showed that the amounts of Ru and Ti species were as low as ∼52 and ∼31 μg/cm^2^, respectively.

**Figure 2:**
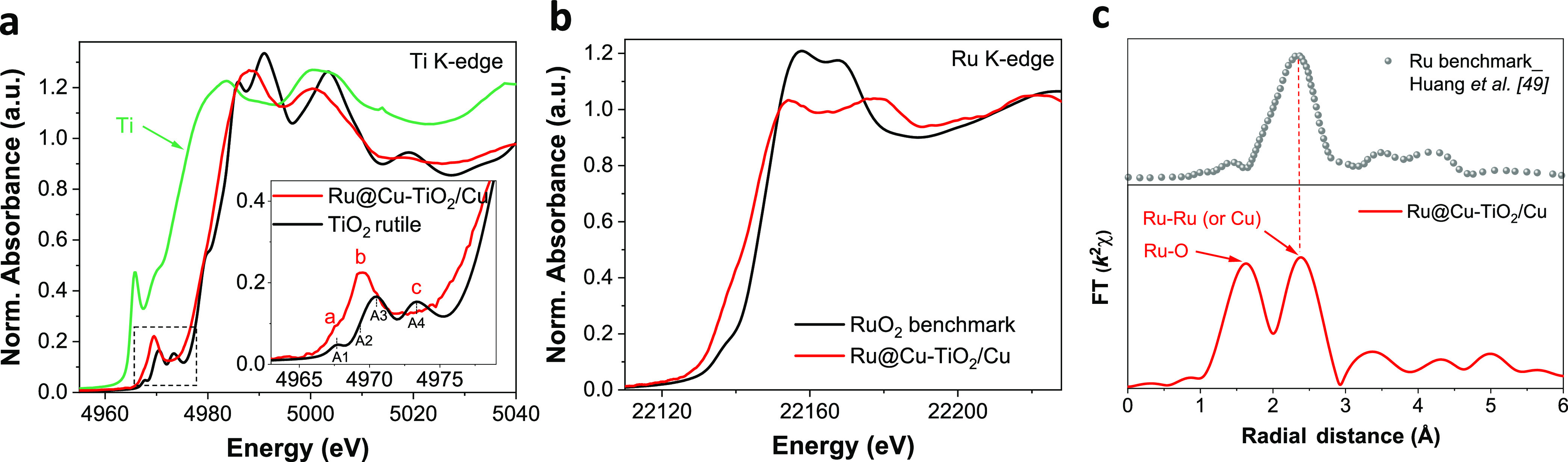
XAS analysis of the as-prepared Ru@Cu–TiO_2_/Cu and benchmarks acquired over the energy ranges for (a) the Ti K-edge, (b) the Ru K-edge, and (c) the corresponding FT-EXAFS spectra showing the bond lengths of Ru–Ru (or Ru–Cu).

XAS was further carried out to analyze the structure of Ru@Cu–TiO_2_/Cu at the atomic level. The X-ray absorption near-edge spectrum (XANES) of the Ti K-edge clearly excluded the presence of metallic Ti (Figure 2a), being its profile similar to that of TiO_2_.^47^ Moreover, in the pre-edge region of the Ti K-edge, our Ru@Cu–TiO_2_/Cu displayed a single prominent peak “b” at 4970 eV, with two very small shoulder peaks “a” and “c” (inset of Figure 2a), similarly to what reported in the literature for amorphous TiO_2_.^47,48^ Considering that in the same region crystalline TiO_2_ is characterized by three prominent peaks and a shoulder peak, our XANES analysis indicates that Ti is present in our electrode in the form of amorphous TiO_2_.^47^ The XANES recorded for the Ru K-edge clearly shows that Ru species within the electrode are not in the form of RuO_2_ (Figure 2b). In fact, the corresponding Fourier Transform of the extended X-ray absorption fine structure (FT-EXAFS) exhibits a prominent main peak at ca. 2.4 Å, which is consistent with Ru–Ru bonds of metallic Ru (Figure 2c).^49^ Noteworthy, it is difficult to differentiate the coordination of Ru–Ru (cluster) from the Ru–Cu coordination (interface) in Ru–Cu nanoheterostructures within Ru@Cu–TiO_2_/Cu NR, as the difference in radii is less than 0.1 Å.^50^ In addition, a peak at ca. 1.5 Å is observed and could be assigned to Ru–O bond (Figure 2c).^50^

### Evaluation of Alkaline HER Performances in a Three-electrode Cell Configuration

**Figure 3:**
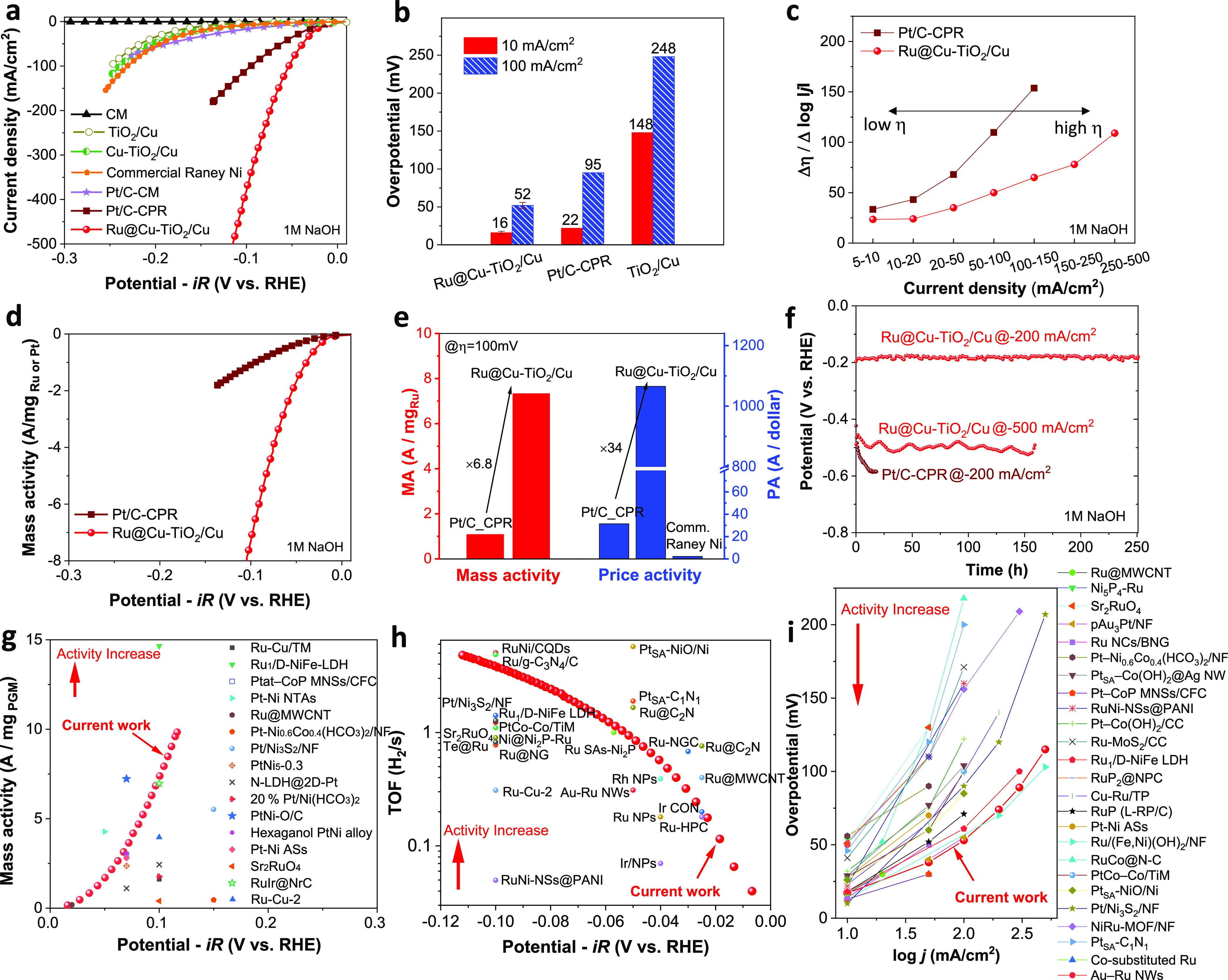
(a) Comparison between the *iR*-corrected LSV curves measured for blank CM, TiO_2_/Cu, Cu–TiO_2_/Cu, commercial Raney Ni, Ru@Cu–TiO_2_/Cu, Pt/C-CM, and Pt/C–CPR (100 μg_Pt_/cm^2^). (b) Overpotentials for the HER at −10 and −100 mA/cm^2^, as extracted from the LSV plots. (c) Δη/Δlog|*j*| ratios of different electrodes. (d) Mass activities measured for Ru@Cu–TiO_2_/Cu and Pt/C–CPR benchmark. (e) Mass activities and price activities measured for the investigated electrodes at 100 mV HER overpotential. (f) Non *iR*-corrected chronopotentiometric potential vs time plots measured for Ru@Cu–TiO_2_/Cu and Pt/C–CPR benchmark. Comparison between the (g) mass activity, (h) TOF and (i) HER activity of our Ru@Cu–TiO_2_/Cu and PGM-based electrocatalysts for the HER in 1 M KOH/NaOH reported in the literature, as listed in Table S2.

The electrocatalytic activities of the investigated electrodes toward the alkaline HER were evaluated in 1 M NaOH electrolyte in a three-electrode cell configuration. As shown in Figure 3a,b, Ru@Cu–TiO_2_/Cu displayed a low overpotential of 16 mV (52 mV) to reach the current density of −10 mA/cm^2^ (−100 mA/cm^2^). This overpotential was lower than those of commercial Raney Ni electrode used as is in AELs (Figure S18) and homemade Pt/C (100 μg_Pt_/cm^2^) on either CM or carbon paper (CPR), with the latter showing an overpotential of 22 mV (95 mV). TiO_2_/Cu (or its derivative of Cu–TiO_2_/Cu, which underwent Ru deposition procedure without Ru precursor participation) exhibited inferior catalytic performance, requiring 248 mV (239 mV) overpotential to reach −100 mA/cm^2^, clearly indicating that the incorporation of Ru nanocrystals is crucial to accelerate the overall HER kinetics (additional data and discussions on the effect of different levels of *iR* correction on the performance of Ru@Cu–TiO_2_/Cu and Pt/C–CPR electrodes are shown in Figures S19 and S20). Interestingly, a “Pt@Cu–TiO_2_/Cu” electrode was additionally produced by replacing the Ru precursor with a Pt precursor (Na_2_PtCl_6_·6H_2_O) during the PGM electrodeposition step. The resulting Pt-based electrode cannot compete with the Ru@Cu–TiO_2_/Cu electrode for the HER in 1 M NaOH, further demonstrating the notable catalytic role of Ru in the alkaline HER process (Figure S21). To reveal the role of TiO_2_ of Ru@Cu–TiO_2_/Cu electrode in alkaline HER catalysis, structure-simplified Ru–Cu-based electrodes with and without the participation of TiO_2_ were prepared and tested, in which the results demonstrate that TiO_2_ could additionally promote the catalytic performance (Figure S22). According to electrochemical impedance spectroscopy measurements on Ru@Cu–TiO_2_/Cu at various overpotentials in 1 M NaOH (Figure S23), the HER proceeds through a combination of Volmer-Heyrovsky and Volmer–Tafel processes. To evaluate the HER performance of the electrodes under high current densities, we analyzed the trend of the Δη/Δlog|*j*| ratio (Figure 3c), where η is the overpotential and *j* is the current density, over the measured current density range separated into several steps.^51^ The Δη/Δlog|*j*| ratio of Pt/C–CPR increased from 34 to 154 mV/dec as the current density range increased from 5–10 mA/cm^2^ to 100–150 mA/cm^2^, while the Ru@Cu–TiO_2_/Cu maintained a ratio as small as 78 mV/dec even in the 150–250 mA/cm^2^ range. This data indicates that Ru@Cu–TiO_2_/Cu retains fast kinetics for the HER even at high current densities (on the order of 100 mA/cm^2^).

To gain a deeper understanding on the electrocatalytic performance of electrodes, the specific activity of electrodes was computed by dividing the (geometric) current density by their corresponding electrochemically active surface area (ECSA) (calculated considering all the material components including nonactive species, see Methods and Figures S24 and S25 in Supporting Information for additional discussion). As shown in Figure S24, although the Ru@Cu–TiO_2_/Cu electrode showcases a lower ECSA-normalized activity at lower working potentials (specific current density) compared to the Pt/C–CPR electrode, it demonstrates the potential for superior performance at higher working potentials. Besides, the analysis of the Tafel plots based on the geometric and ECSA-normalized current densities evidenced that the Ru@Cu–TiO_2_/Cu exhibits the fastest HER kinetics among the investigated electrodes (see discussion in Figures S26 and Note S1). By considering the electrode mass activity (Figure 3d, calculated by normalizing the geometric current density to mass loading of PGMs, i.e., Ru or Pt), the Ru@Cu–TiO_2_/Cu significantly outperforms the Pt/C–CPR benchmark (at 100 mV HER overpotential: 7.33 A/mg_Ru_ vs 1.08 A/mg_Pt_) (Figure 3e). Importantly, at 100 mV HER overpotential, the price activity of Ru@Cu–TiO_2_/Cu (calculated as the electrode current normalized to the cost of PGMs) was found to be 1065.4 A/US$_Ru_, which is much higher than that of the Pt/C–CPR benchmark (31.4 A/US$_Pt_) and commercial Ni electrode (2.27 A/US$_Ni_) (Figure 3e, see calculations in the Supporting Information). Moreover, Ru@Cu–TiO_2_/Cu showed durable HER activity for over 250 h (@-200 mA/cm^2^) and 160 h (@-500 mA/cm^2^) (Figure 3f), without exhibiting significant morphological (Figure S27) and compositional (Figure S28) modifications. This may be due to the highly conductive Cu NRs skeleton, which is resistant to detachment under strong H_2_ evolution conditions compared to binder-involving nanostructured catalysts.^11^ In contrast, the HER overpotential of Pt/C–CPR benchmark increased significantly (>100 mV) within 12 h (Figure 3f). The Faradaic efficiency of the HER measured on Ru@Cu–TiO_2_/Cu operating at −20 mA/cm^2^ was found to be ∼100% by gas chromatography (Figure S29). Overall, these results evidenced that Ru@Cu–TiO_2_/Cu achieved superior performances compared to those of the conventional Pt/C benchmark, while relying on a low PGM loading (i.e., ∼52 μg_Ru_/cm^2^ for Ru@Cu–TiO_2_/Cu vs 100 μg_Pt_/cm^2^ for Pt/C). Noteworthy, our Ru@Cu–TiO_2_/Cu also outperforms most of the HER catalysts based on PGMs reported in literature (Figure 3g and Table S2). To better compare the catalytic activities of the electrodes, the turnover frequency (TOF) of the HER is shown in Figure 3h. Importantly, although it makes more sense to consider both the Ru and its surface Cu species for a more comprehensive analysis, accurately singling out these Cu atoms from all the other “inactive” Cu species, such as those present in the NR and Cu substrate, presents a formidable challenge. Consequently, we have determined the TOF by considering all the Ru atoms as active sites, recognizing that this approach unavoidably leads to an underestimation of the TOF value. Specifically, at a 100 mV HER overpotential, our Ru@Cu–TiO_2_/Cu exhibits a TOF of 3.85 s^–1^, which is much higher than those reported for previous PGM-based electrocatalysts (Table S3). Although catalysts based on single atoms can exhibit TOFs comparable or even higher than that of Ru@Cu–TiO_2_/Cu,^52^ the latter features obvious advantages in terms of both synthesis easiness and catalytic activity toward the HER under high current densities (≥100 mA/cm^2^), which are fundamental aspects to consider for practical AELs (Figure 3i and Table S2). In addition, three repeated syntheses of Ru@Cu–TiO_2_/Cu led to a similar catalytic performance, demonstrating the reproducibility of the electrode fabrication process (Figure S30a). The electrode was also easily scaled up (e.g., up to 4 cm^2^) with negligible performance decay (Figure S30b). Moreover, our synthetic protocol is flexible to changes in the metal source for sputter coating (Figure S31).

Ru@Cu–TiO_2_/Cu was also evaluated under AEL-simulating operating conditions in a three-electrode-configuration, that is, highly alkaline media (6 M NaOH, equals ∼20 wt % NaOH) and temperatures up to 80 °C. The electrode exhibited a stable HER-overpotential at a current density of −500 mA/cm^2^ (Figure S32), without any degradation of its morphology (Figure S33). As expected, the mass transport/diffusion in such conditions is accelerated compared to those recorded at ambient temperature and low-concentration aqueous electrolytes (Figure S32c). Such effect improves the overall activity of the electrodes compared to those measured at ambient temperature in 1 M NaOH, whose limited conductivity (∼10 S/m) would also increase the ohmic resistance associated with diaphragms in practical AELs, as targeted hereafter in this work.

### Operando XAS Characterization

**Figure 4:**
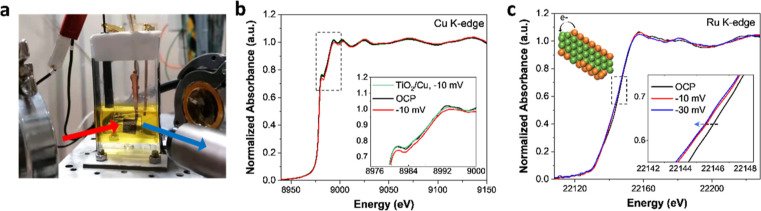
(a) Setup of the operando XAS measurements. The red-line indicates the incident X-ray, while the blue-line indicates the fluorescence X-ray. (b) Operando XANES spectra of the Cu K-edge recorded on Ru@Cu–TiO_2_/Cu and TiO_2_/Cu at different applied potentials, i.e., the OCP and −10 mV (vs RHE). (c) Operando XANES spectra of the Ru K-edge recorded on Ru@Cu–TiO_2_/Cu at different potentials, i.e., the OCP, −10 and −30 mV (vs RHE). Inset in (c) depicts the charge transfers from Cu to Ru under HER conditions. All the XANES spectra were smoothed using the adjacent-averaging method (points of window: 8). The raw data are also reported in Figure S35.

To reveal the origin of the high catalytic activity of Ru@Cu–TiO_2_/Cu, operando XAS spectra were recorded in a homemade electrochemical cell (Figure 4a) by probing the electrode during the HER. The working electrode was tested under open-circuit potential (OCP), −10, and/or −30 mV (vs RHE). Such low working potentials were used purposefully to avoid massive H_2_ evolution, which may lead to a noisy signal. Figure 4b displays the operando Cu K-edge spectra of Ru@Cu–TiO_2_/Cu working at the OCP and −10 mV (vs RHE). The spectrum recorded for a control sample of TiO_2_/Cu working at −10 mV (vs RHE) is also shown for comparison. As displayed in Figure 4b, no clear shift of the XANES spectra could be observed on the Cu K-edge for both Ru@Cu–TiO_2_/Cu and TiO_2_/Cu electrodes by increasing the working potential from the OCP to −10 mV (vs RHE). Nevertheless, the analysis of FT-EXAFS spectra for Ru@Cu–TiO_2_/Cu (Figure S35c) indicates that structural changes are occurring in the sample, for instance, on bond length or coordination number, of the Cu–Cu/Ru shell under −10 mV (vs RHE), compared to the case at the OCP, while no obvious change was observed for the TiO_2_/Cu under −10 mV (vs RHE). This may suggest that the applied potentials promote the interaction between Cu and Ru within Ru@Cu–TiO_2_/Cu, consistent with the study of Wu et al..^30^ Hence, the nonobservable shift on its Cu K-edge spectra could be due to the fact that the content of Cu species interacting with Ru is very limited compared to the bulk of Cu in the NR core. However, the XANES spectra recorded on Ru@Cu–TiO_2_/Cu at the Ru K-edge shift toward lower energies as the applied potentials increases from OCP to −30 mV (vs RHE), indicating a slight decrease of the Ru valence state under HER working conditions (Figure 4c). Since the ex situ XPS characterization revealed an electron transfer from Cu to Ru (Figure S17b,c), the Cu-to-Ru electron transfer could be further facilitated under HER working conditions (inset in Figure 4c). Ti species within Ru@Cu–TiO_2_/Cu are not detectable due to the low content, light-element feature, and the spectral noise caused by H_2_ bubbling.

### First-Principles Simulations

Density functional theory (DFT) simulations were performed to gain insights into the structure and HER activity of Ru@Cu–TiO_2_/Cu. We studied the energetics of the Volmer step and hydrogen binding energy (HBE). The Volmer step is the reaction through which hydrogen is adsorbed, that is, in alkaline media^53^
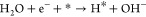
1where * represents a surface site and H* a hydrogen atom adsorbed to the surface. This reaction can be viewed as occurring through three steps, namely, water adsorption, water dissociation, and hydroxyl desorption
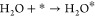
2
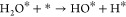
3
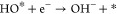
4we label the corresponding reaction energies as Δ*E*_H_2_O_^ads^, Δ*E*_H_2_O_^diss^, and Δ*E*_OH^–^_^desorb^, respectively. For the dissociation, reaction 3, a relevant role may be played by its kinetic barrier. However, based on the Brønsted–Evans–Polanyi principle,^54^ which was shown to apply also to heterogeneous catalysis^55^ and to the HER,^14^ Δ*E*_H_2_O_^diss^ is linearly correlated to the kinetic barrier and it is thus adopted here as an indicator of the water dissociation rate. Finally, HBE, measured by the energy change (Δ*E*_H_2__^desorb^) of the reaction
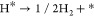
5represents the barrier for the final HER step. It is a key quantity for measuring the ability of a catalyst to desorb H_2_, regardless of the mechanism involved (i.e., Heyrovsky or Tafel).^56^ We exploit thermodynamical considerations and tabulated experimental data to calculate the energy change of reaction 4 and the free energy changes (Δ*G*) associated with reactions 1 and 5 in an efficient and accurate manner; see Experimental Section and Note S3 of the Supporting Information.

**Figure 5:**
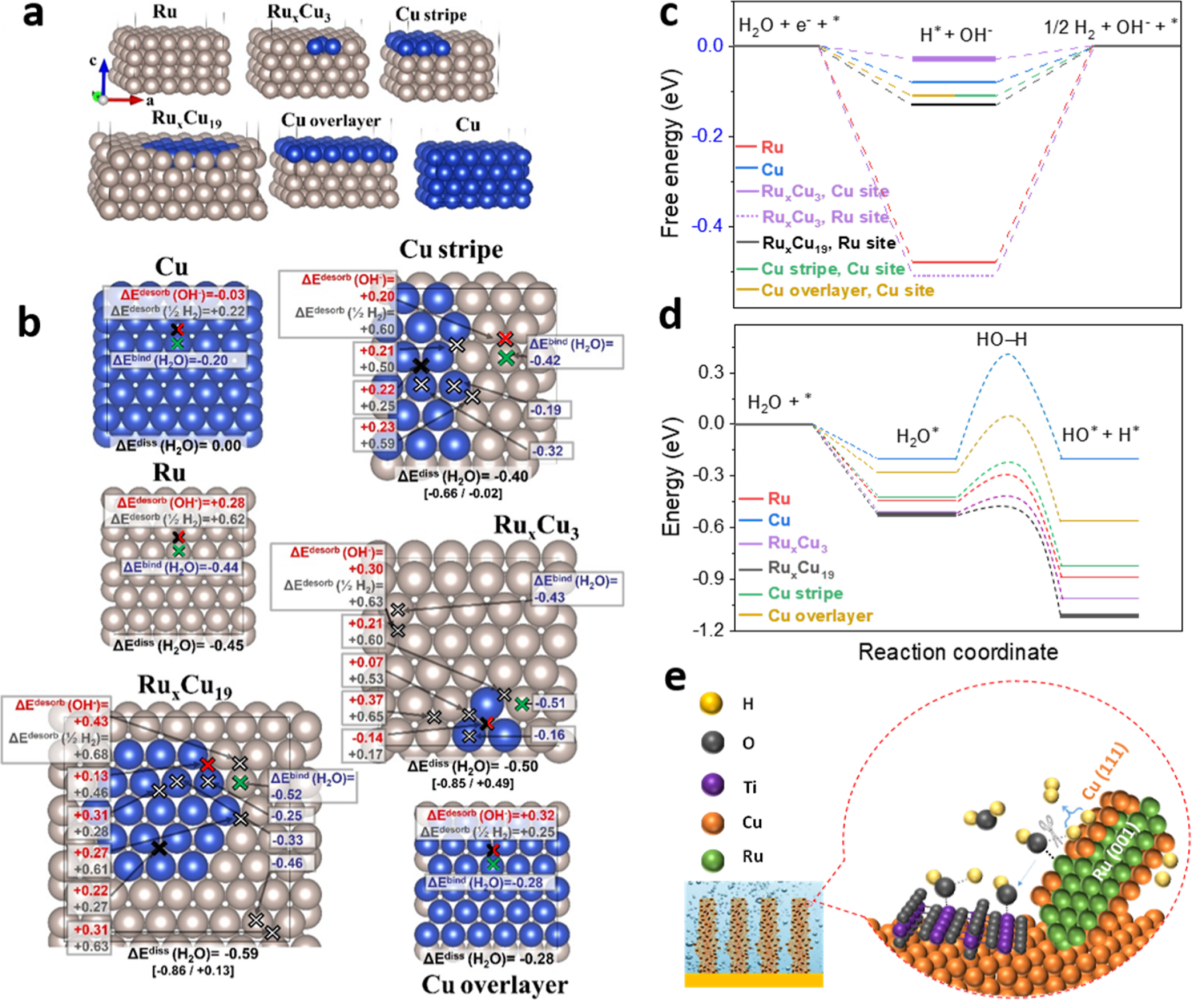
(a) Systems considered in first-principles simulations with their corresponding labels. (b) Reaction energies (eqs 2–5 of the main text) on the most representative surface sites. Note that only the most favorable types of adsorption sites were considered, namely, hollow-fcc for H and OH, and top for H_2_O. The black and red crosses indicate, respectively, the optimal sites for H and OH desorption, while green crosses label the most stable sites for H_2_O binding. White crosses mark all the other the considered adsorption/desorption sites. The values for the water dissociation energy (reaction 3 of the main text) are reported below each surface. The main value of Δ*E*^diss^(H_2_O) is obtained by considering the most stable adsorption sites for H_2_O, H, and OH for each surface, with H and OH adsorbed at a large distance from each other. Instead, the range in square brackets reported right below represents the minimum and maximum values for water dissociation energy obtained considering all of the investigated adsorption sites. (c) Calculated free energy diagram for the Volmer step and hydrogen desorption (see the Experimental Section for details). (d) Calculated energy for water adsorption and H_2_O-dissociation, with a qualitative representation of the kinetic barrier based on the Brønsted–Evans–Polanyi principle (see the discussion in the main text). The energy value of each item shown in diagrams (c,d) is calculated considering the optimal adsorption/desorption sites from (b). Steps marked in bold indicate the most favorable reaction path. (e) Schematic showing the mechanism of HER on Ru@Cu–TiO_2_/Cu.

Our analysis focused on the Cu/Ru part of our catalyst, as TiO_2_ has been widely studied and is known to dissociate water efficiently.^38,39^ Being immiscible,^44–46^ Cu and Ru form a nanoheterostructure, with a nonuniform distribution at the atomic level and possible segregation. Our simulations show that the Cu migration from the bulk of the sample to the surface of Ru is energetically favorable (Note S3.2), in line with the lower surface energy of Cu compared to that of Ru,^40^ and with the STEM–EDS elemental mapping shown in Figures 1g–l. Instead, the formation of Ru bulk or surface islands within Cu matrices is strongly endothermic (Note S3.2), hence unlikely to take place appreciably. Given this complex structural scenario, our catalyst was modeled by considering, beside the pure elements, Cu-substituted Ru surfaces ranging from three-atom nanoislands to a complete Cu overlayer (Figure 5a and S36).

Pure Cu and Ru display two different behaviors toward the HER (Figure 5b). Ru spontaneously dissociates water,^57^ while its Δ*E*_OH^–^_^desorb^ and Δ*E*_H_2__^desorb^ are strongly positive. On the contrary, Cu has a sluggish water dissociation step, but it desorbs HO* spontaneously and H_2_ with a small barrier.

The presence of Ru (Cu) atoms around Cu (Ru) adsorption sites has a moderate impact on Δ*E*_H_2__^desorb^ (Figure 5b,c), while it significantly changes Δ*E*_H_2_O_^ads^, Δ*E*_H_2_O_^diss^, and Δ*E*_OH^–^_^desorb^ (Figure 5b). In general, the reaction energies have a nonlinear trend with the size of Cu coverage. For example, Δ*E*_H_2__^desorb^ on Cu sites decreases when going from pure Cu to small Cu islands of Ru–Cu and then increases with the size of the Cu island (Figure 5b,d). Simulations on model systems (Note S4.3) show that the Ru matrix influences the reaction energies on the Cu surface atoms through strain and electronic effects, both being significant in magnitude. A small Cu to Ru charge transfer is observed from atomic charges (Table S5), in agreement with experimental findings (Figure 4c). Overall, our simulations outline a picture in which different catalytic species act synergistically in performing the overall HER. Ru surface effectively dissociates water, and this process is accelerated in the presence of a surface Cu cluster nearby (Figure 5d). The desorption of HO*, which is crucial to avoid the catalyst poisoning,^14^ and H_2_ formation occurs on pure Cu and on finely dispersed Cu clusters. In general, the formation of Cu–Ru interfaces creates a range of intermediate adsorption/desorption energies (Figure 5b) that may be beneficial for the catalytic performances.^58^ TiO_2_ can further promote the water dissociation reaction or, possibly, acts as a reservoir for HO*, thereby freeing HO* from Ru sites that can further act as water dissociation centers (Figure 5e).

### Evaluation of Practical H_2_ Production in AEL

**Figure 6:**
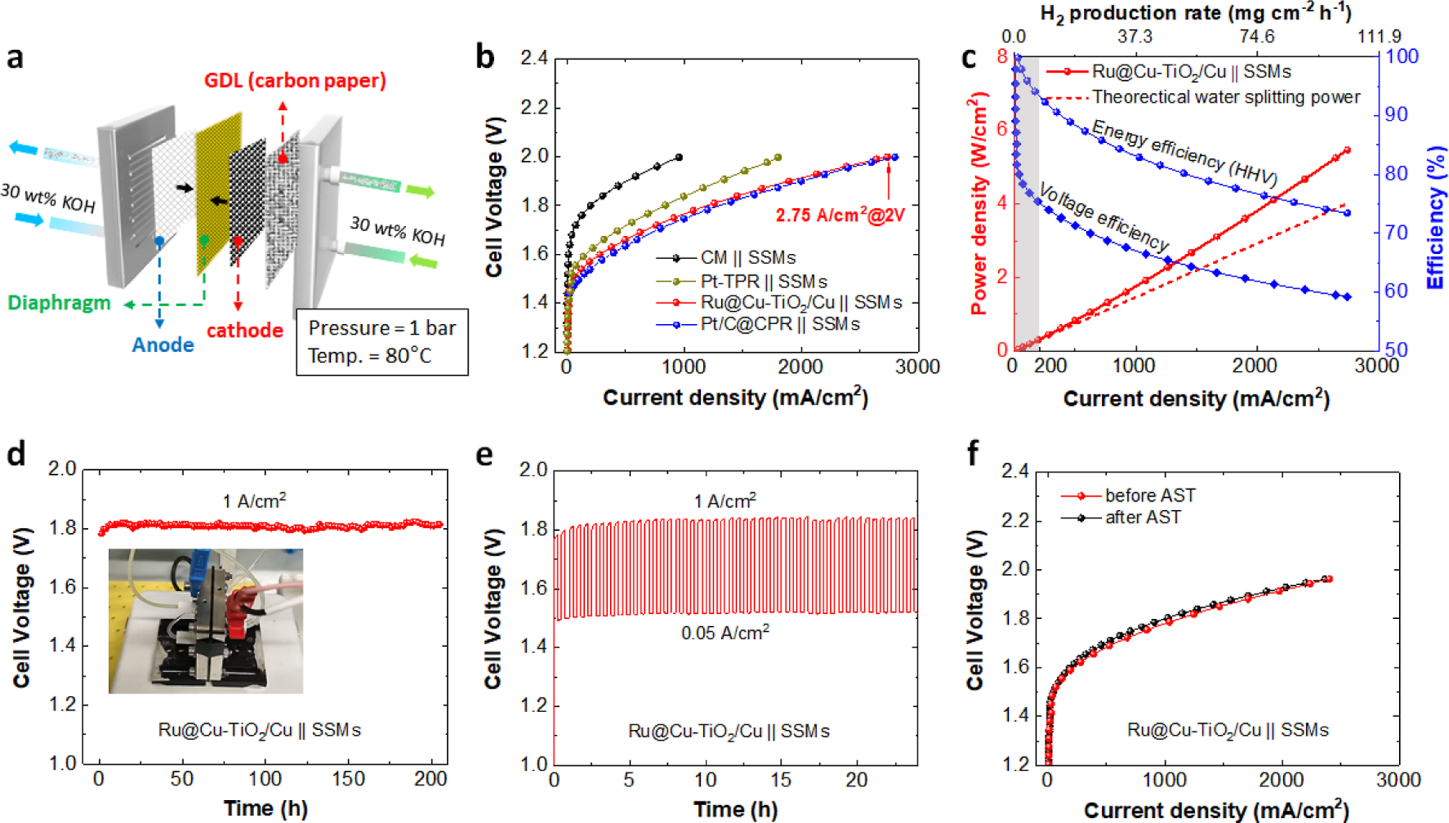
(a) Sketch of the AEL configuration. (b) Polarization curves measured for AELs based on Zirfon Perl UTP 220 diaphragm, SSMs as the anode, and different cathodes: Ru@Cu–TiO_2_/Cu, Pt-PTR, Pt/C–CPR, and CM. A CPR GDL was used as extra GDL at the cathode side. (c) Power, H_2_ production rate, energy efficiency (based on the H_2_ HHV), and voltage efficiency as a function of the current density for Ru@Cu–TiO_2_/Cu∥SSMs AEL. The theoretical power for water splitting is based on the thermoneutral voltage at 80 °C and 1 bar. Gray shading indicates an operating region corresponding to poorly practical current densities (<0.20 A/cm^2^). (d) Stability measured for the Ru@Cu–TiO_2_/Cu∥SSMs AEL operating continuously at 1 A/cm^2^ for over 200 h. Inset depicts the setup of AEL configuration. (e) 24 h-AST of the Ru@Cu–TiO_2_/Cu∥SSMs AEL. (f) Polarization curves measured on Ru@Cu–TiO_2_/Cu∥SSMs by AEL before and after the AST.

The optimized Ru@Cu–TiO_2_/Cu was validated as a cathode into atmospheric-pressure AELs based on an anode made of stainless-steel (stacked SSMs), an inexpensive and robust catalyst for the OER,^9,59^ at 80 °C and using 30 wt % KOH as the electrolyte (Figure 6a). Different AELs based on the investigated cathodes, namely, CM, commercially platinized titanium paper (Pt-TPR), and Pt/C–CPR (*m*_Pt_ = 75 μg/cm^2^), were also evaluated for comparison. Hereafter, AELs are named cathode∥anode. Zirfon PERL UTP 220 was selected as the diaphragm separator due to its excellent ionic conductivity areal resistance ∼0.079 Ω cm^2^ in 30 wt % KOH and 80 °C, as shown in (Figure S37) and low hydrogen crossover (anodic hydrogen content typically <2%, or even <0.2% at operating current density ≥500 mA/cm^2^).^60^ The polarization curves measured for the produced AELs indicate that the AEL based on Ru@Cu–TiO_2_/Cu cathode outperforms those based on Pt-TPR and CM cathodes, and is comparable with that built on Pt/C–CPR. The Ru@Cu–TiO_2_/Cu∥SSMs AEL required cell voltages of 1.66 and 1.77 V to reach current densities of 0.5 A/cm^2^ and 1.0 A/cm^2^, respectively (Figure 6b). These values correspond to energy efficiencies of 88.5 and 83.0%, respectively (based on the H_2_ higher heating value -HHV-, Figure 6c) or voltage efficiencies of 71.3 and 66.9%. Our AEL reached a current density as high as 2.75 A/cm^2^ at a cell voltage of 2.0 V. Meanwhile, the developed AELs could offer the robustness of traditional AELs, as established at MW-scale for over a century.^10^ Indeed, our AEL operated at 1.0 A/cm^2^ with no further performance decay after the initial 5 h for stabilization, namely, the cell voltage is stable at ca. 1.8 V for at least 200 h (Figure 6d). To the best of our knowledge, such performance is superior to that of previous reports on the AEL configuration. Although some electrolyzers based on AEM/PEM with higher performance have been reported previously, either their stability has not been evaluated or they suffered from rapid degradation of performance, that is, average voltage increase rate of several mV/h at constant current densities, in many cases even lower than 1 A/cm^2^ (see Table S7 and comments in its Note).

Noteworthy, although the overall 3D structure of the NR array has been retained for Ru@Cu–TiO_2_/Cu after the stability test (Figure S38), XPS analyses reveal an increase of Cu content at the electrode surface (Figure S28). Besides, the ICP–OES result demonstrates that most of the Ru remains on the electrode after stability operation, while HAADF-STEM (and EDS mapping) analyses indicate that the Cu is still grown on top of Ru, with the former species becoming concentrated compared to that in the fresh sample (Figure S39). The first-principles simulations we discussed above supported this phenomenon. In addition, the intermittent operation of the AEL was also evaluated through an in situ AST protocol, designed to evaluate the compatibility of our technology with renewable energy supply conditions (see details in Methods of the Supporting Information). As shown in Figure 6e, our AEL operated at a nearly stable cell voltage during consecutive 1.0 A/cm^2^ steps, demonstrating its reliability under intermittent operating conditions. Besides, no obvious decay of the performance was observed from the polarization curves after the AST (Figure 6f). Hence, the above results prove the stability of Ru@Cu–TiO_2_/Cu during practical AEL operation.

Interestingly, we observed that Ru@Cu–TiO_2_/Cu outperforms the Pt/C–CPR in a three-electrode cell setup, but they also permit the reach of similar performance to Pt/C–CPR while reducing the PGM mass loading by 50%. Prospectively, further studies could be oriented toward the identification of gas diffusion layers (GDLs) that are specific for our nanostructured cathodes, aiming at minimizing the contact resistance at the electrode/bipolar plate interface, while optimizing the H_2_ gas collection to reduce the polarization losses associated with the formation of gas bubbles at the electrode/electrolyte interface.

### Techno-Economic Analysis

A preliminary techno-economic analysis (TEA) has been carried out to evaluate the levelized cost of the hydrogen (LCOH) produced by an ideal 1 MW (net power)-scale AEL implementing the single cell technology outlined in this work, while assuming the unit price of electricity is US$20/MW h.^61^ In particular, the impact of the operative current density and cell voltage has been reported hereafter, while the detailed discussion of the TEA, including CAPital EXpenditure (CAPEX) and OPerational EXpenditure (OPEX) breakdowns for the most profitable operative conditions, is available in the Supporting Information.

**Figure 7:**
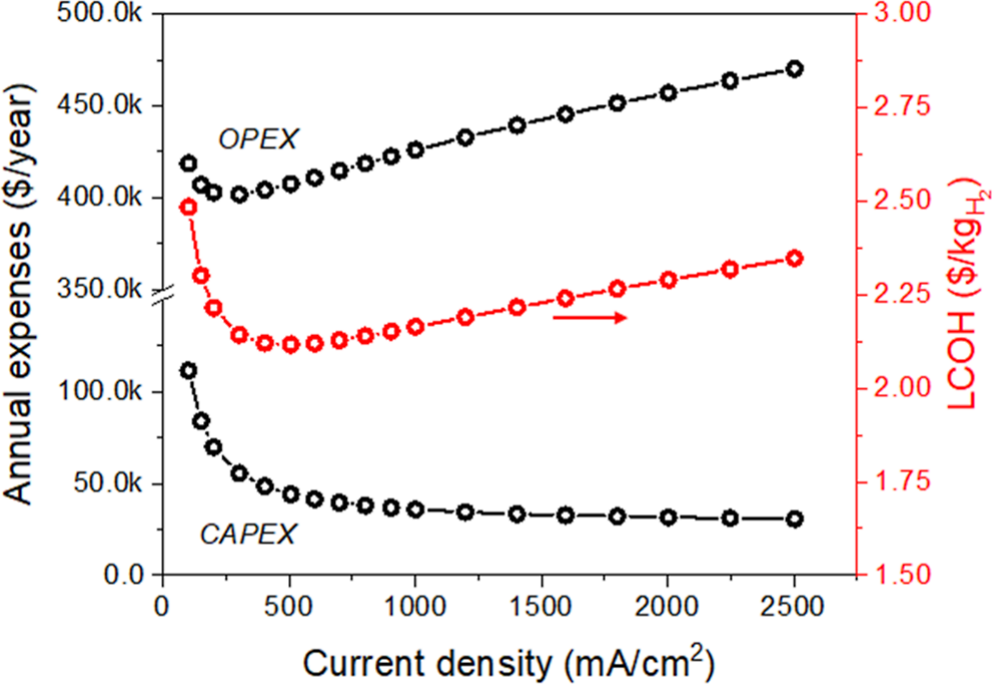
CAPEX, OPEX, and LCOH were obtained as a function of the operative current density. All data reported in the present figures have been calculated using H_2_ HHV.

As expected, CAPEX (Figure 7 and S40) associated with the deployment of the plant decreases exponentially when moving to high current densities in virtue of the diminished number of cells required to achieve the target 1 MW net power (Figure 7). On the contrary, the OPEX displays a more complex trend (Figure 7), which reflects the multiple dependences of its constituting entries. With the increase in the operative current density (and the related flattening of the CAPEX curve), the OPEX becomes the major contributor to the LCOH, a typical effect related to electrolyzers upscaling.^61^ In particular, the actual electrolysis (i.e., the electric energy consumption, OPEX_Electricity_) progressively takes the largest OPEX shares (annexed Excel Spreadsheets), emphasizing the importance of the electrochemical performance in determining the LCOH.

The dependence of the LCOH on the operative current density reflects the combination of CAPEX and the OPEX trends (Figure 7). Consequently, LCOH reaches the minimum value when the AEL is operated at 500 mA/cm^2^ (@ 1.66 V single cell voltage), achieving a value as low as US$2.12/kg_H_2__. Noticeably, the LCOH is lower than US$2.50/kg_H_2__ throughout the whole range of current density under investigation (Figure 7, annexed Excel Spreadsheet), evidencing that the Ru-based cathodes reported herein are promising catalytic systems for massive H_2_ production. Additional details on TEAs, including LCOH dependence on AEL plant lifetime and CAPEX and OPEX breakdown of 1 MW-scale AEL plants, are reported in Figures S41–S44.

## Conclusions

We have reported a cost-effective fabrication of a Cu mesh-supported nanostructured HER electrocatalyst composed of vertical NRs made of Cu decorated with amorphous TiO_2_ nanoparticles and Ru–Cu nanoheterostructures. The resulting Ru@Cu–TiO_2_/Cu electrode exhibits an excellent catalytic performance toward the HER in alkaline media, sustaining an industrial-level current density of −200 mA/cm^2^ for more than 250 h, and −500 mA/cm^2^ for over 160 h, outperforming Pt/C benchmarks. Based on experimental investigations and theoretical calculations, the excellent activity of Ru@Cu–TiO_2_/Cu toward the HER in alkaline media was ascribed to: (1) the very robust porous 3D structure of the Cu NRs array providing not only a high electrical conductivity and a large electrochemically active surface area, but also withstanding even a strong H_2_ bubble releasing; (2) the presence of Ru nanocrystals acting as efficient water dissociation centers, whose interaction with surface Cu clusters further accelerates the Volmer step; (3) the surface Cu clusters interacting with Ru, providing close-to-zero Gibbs free energy of the hydrogen adsorption–desorption. Ru@Cu–TiO_2_/Cu was then validated as the cathode in a lab-scale AEL, reaching 1.0 A/cm^2^ at a low cell voltage of 1.77 V, corresponding to an energy efficiency of 83.0% (based on the H_2_ HHV) and voltage efficiency of 66.9%. Such AEL stably operated under continuous (1 A/cm^2^ for over 200 h) and intermittent (i.e., accelerated stress test) modes in 30 wt % KOH at 80 °C. These performances led to an estimated overall H_2_ production cost of only US$2.12/kg_H_2__ (1 MW AEL plant with 30 year-lifetime), almost meeting the worldwide targets for the cost of green hydrogen set by the US^62^ and EU^63^ (US$2–2.5/kg_H_2__). Our work depicts that hierarchical electrode architectures, composed of multiple catalytic species working in tandem for different HER steps, could be suitable for an efficient industrial generation of green H_2_.

## Experimental Section

### Preparation of 3D Structured Ru@Cu–TiO_2_/Cu NRs Array Grown on CM Surface

The preparation of Ru@Cu–TiO_2_/Cu NRs grown on CM included the following three steps:

#### Synthesis of the Skeleton made of 3D Cu(OH)_2_ NRs on CM

A piece of precleaned CM (2 cm × 4 cm) was immersed in a solution mixture (30 mL) of 0.1 M ammonium persulfate and 2 M sodium hydroxide for 30 min. In this process, CM was directly used as the Cu precursor, and Cu(OH)_2_ NRs spontaneously grew on the surface of CM. The obtained Cu(OH)_2_ NR electrode was washed with Milli-Q water and dried by using an N_2_-gun stream.

#### Sputtering of Cu and Ti Layers onto the Surface of Cu(OH)_2_ NRs

The obtained Cu(OH)_2_ NRs electrode was then placed in a sputter coater (Q150T ES PLUS) to first deposit a Cu layer (film thickness monitor -FTM- = 60 nm, tooling factor = 3.4) and then a Ti layer (FTM = 30 nm, tooling factor = 3.4) onto the surface of Cu(OH)_2_ NRs. The resulting electrode, named the Ti@Cu@Cu(OH)_2_ NR, was partially oxidized upon air exposure, forming surface layers of copper oxides and titanium oxides, leading to the TiO_2_@CuO@Cu(OH)_2_ NR electrode. When the thickness of layer coatings was indicated, the produced electrode was defined as *y*TiO_2_@*x*CuO@Cu(OH)_2_ NRs, where *x* and *y* indicate the thicknesses, expressed in nm, of sputtered Cu (CuO) and Ti (TiO_2_), respectively.

#### In Situ Deposition of Ru Nanocrystals to Produce the Ru@Cu–*y*TiO_2_/*x*Cu (Target Eectrode)

The obtained *y*TiO_2_@*x*CuO@Cu(OH)_2_ NR electrode (Figure S1a) was cut into the desired size (typical working area: 1 cm^2^) and immersed in a 1 M NaOH solution (25 mL). Subsequently, a negative current density of −5 mA/cm^2^ was applied to a three-electrode cell configuration. To do so, the Cu(OH)_2_ skeleton was slowly reduced, transforming the oxidized Cu layer into metallic Cu obtaining the TiO_2_/Cu electrode. Figure S1b,c show the CP plot, until the electrode potential became stable, indicating the end of the electrochemical reduction protocol. Afterward, Ru nanocrystals were electrodeposited onto the TiO_2_/Cu surface by adding 400 μL of K_2_RuCl_6_ aqueous solution (1 mg/mL) and by applying a negative potential of −0.2 V (vs RHE). The optimized electrode prepared following above-mentioned steps, namely, Ru@Cu–30TiO_2_/60Cu, was simply denoted as Ru@Cu–TiO_2_/Cu for clarity. Otherwise, the thickness of the coating layer was indicated as Ru@Cu–*y*TiO_2_/*x*Cu.

### First-Principles Simulations and Evaluation of the Reaction (Free) Energies

DFT simulations were performed through the Vienna ab initio simulation package (VASP)^64^ adopting the Perdew–Burke–Ernzerhof functional.^65^ More details are reported in Note S2. The energies of reactions 2, 3, and 5 were calculated as the energy difference between products and reactants, see Note S2. For reaction 4, we instead calculate the energy of the reaction
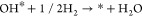
6rather than that of reaction 4 of the main text. In fact, what we demonstrate in Note S3.1 that, at the ideal electrochemical potential adopted for alkaline HER, the free energy of eq 6 is fully equivalent to that of eq 4. The adoption of eq 4, however, overcomes the complexity of accounting for the electron potential and the solvation energy of the OH^–^ ion. Concerning the evaluation of free energies of Figure 5c, we calculated the free energy of hydrogen desorption (eq 5) through the approach of Norskov et al.,^66^ corrected by the entropy of adsorbed hydrogen (see Note S2).

*Note: Many other details, for example, preparation of control electrodes, instrumental characterizations, simulation methodology, TEA, and so forth, can be found in the Supporting Information*.

## Supporting Information Available

Experimental, characterization, and simulation details; SEM, SEM–EDS, HR-STEM, STEM–EDS, and XPS analyses; electrochemical measurements; DFT models of Ru–Cu nanoheterostructures; ECSA analysis; H_upd_ analysis; Tafel slope analysis; specific activity analysis; Faraday efficiency analysis using gas-chromatography; upscaling synthesis; analysis on different counter electrodes; additional operando XAS spectra; additional analysis on AEL; and details on TEA (PDF)
TEA for our proposed AEL with a fixed net power (XLSX)
