# Supplementary material for: Ru–Cu Nanoheterostructures for Efficient Hydrogen Evolution Reaction in Alkaline Water Electrolyzers
Source: J Am Chem Soc. 2023 Sep 25;145(39):21419–31. doi: 10.1021/jacs.3c06726 (PMC10557145; doi:10.1021/jacs.3c06726)
Supplement: Supplementary file 1 — ja3c06726_si_001.pdf [file ja3c06726_si_001.pdf]

Supporting Information

for

Ru-Cu nanoheterostructures for efficient hydrogen evolution reaction in alkaline water electrolyzers

Yong Zuo[1]*, Sebastiano Bellani[2]*, Gabriele Saleh[1], Michele Ferri[1], Dipak V. Shinde[1§], Marilena Isabella Zappia[2], Joka Buha[1], Rosaria Brescia[3], Mirko Prato[4], Roberta Pascazio[1,5], Abinaya Annamalai[1], Danilo Oliveira de Souza[6], Luca De Trizio[1], Ivan Infante[1,7,8], Francesco Bonaccorso[2,9], Liberato Manna[1]*

[1] Nanochemistry Department, Istituto Italiano di Tecnologia, Via Morego 30, 16163 Genova, Italy

[2] BeDimensional S.p.A., Via Lungotorrente Secca, 30R, 16163 Genova, Italy

[3] Electron Microscopy Facility, Istituto Italiano di Tecnologia, Via Morego 30, 16163 Genova, Italy

[4] Materials Characterization Facility, Istituto Italiano di Tecnologia, Via Morego 30, 16163 Genova, Italy

[5] Department of Chemistry and Industrial Chemistry, Università degli Studi di Genova, Via Dodecaneso 31, 16146 Genova, Italy

[6] ELETTRA Sincrotrone Trieste S.C.p.A., S.S. 14 Km 163.5, 34149 Trieste, Italy

[7] BCMaterials, Basque Center for Materials, Applications, and Nanostructures, UPV/EHU Science Park, Leioa 48940, Spain

[8] Ikerbasque, Basque Foundation for Science, Bilbao 48009, Spain

[9] Graphene Labs, Istituto Italiano di Tecnologia, Via Morego 30, 16163 Genova, Italy

[§] Present address: National Physical Laboratory, Hampton Road, Teddington, TW11 0LW, UK

Table of Contents

Methods 3

Chemicals 3

Preparation of 3D structured Ru@Cu-TiO_2/Cu NRs array grown on CM surface 3

Preparation of control electrodes based on the commercial Pt/C 4

Characterization of electrode materials 4

Electrochemical tests of the electrodes 4

X-ray absorption spectroscopy measurements 6

First-principles simulations 7

Electrochemical tests of the AEL 7

Techno-economic analysis 8

Figure S1-S44

Discussion on Ru-Cu miscibility and possible alloying 23

Note S1. Discussion on Tafel slope below 30 mV/dec 37

Table S1. Mass loadings of Ti in Ru-yTiO_2/60Cu, as determined by ICP-OES measurements. 45

Table S2. Comparison between the HER activities of various PGM-based catalysts in 1 M KOH/NaOH reported in recent literature. 46

Table S3. Comparison between the TOF of various PGM-based catalysts in 1 M KOH/NaOH reported in recent literature. 49

First-principles simulations 50

Note S2: Methods for density functional theory simulations 50

Note S3: Thermodynamic considerations 51

Table S4. Substitution energies on various Cu-Ru slab as calculated by DFT simulations. 53

Note S4: Additional simulation results 54

Table S5. Atomic charges in three representative systems 54

Table S6. Adsorption energies on Cu, Ru, Cu-overlayer model (see main text) 55

Evaluation of AEL 56

Table S7. Comparison between the water splitting performances of our AELs and those of electrolyzers (including PEM and AEM ones) reported in recent literature. 59

Calculation of mass and price activity 65

Estimation of operating cost for H_2 production in our AELs 66

Techno-economic analysis (TEA) 67

Table S8. CAPEX-related parameters that have been set/retrieved from different sources throughout the TEA. 71

Table S9. OPEX-related parameters assumed in the TEA. 72

Table S10. Financial parameters assumed in the TEA. 72

Table S11. Electrochemical and process-related parameters assumed in the TEA. 72

References 76

Methods

Chemicals

Ammonium persulfate (98%), sodium hydroxide (NaOH) (98%), potassium hexachlororuthenate(IV) (K_2RuCl_6) (99.95%) were purchased from Sigma-Aldrich. CM (60 mesh, 0.19 mm thickness) and SSM (80 mesh, 0.18 mm thickness, Type 316) were purchased from Fisher Scientific. Mesh substrates were cleaned with isopropanol/ethanol (1:1, v/v), distilled water, and HCl solution (1 M), and dried using a N_2 stream. CPR (AvCarb MGL280), used as GDL in the AEL cathodes, were purchased from FuelCell Store. Zirfon Perl UTP 220, used as diaphragm in the AELs, was purchased from Agfa. Pt-TPR was purchased from FuelCell Store.

Preparation of 3D structured Ru@Cu-TiO_2/Cu NRs array grown on CM surface

As shown in **Figure 1a**, the preparation of Ru@Cu-TiO_2/Cu NRs grown on CM included the following three steps:

a. Synthesis of the skeleton made of 3D $Cu(OH)_2$ NRs on CM: a piece of pre-cleaned CM (2 cm×4 cm) was immersed in a solution mixture (30 mL) of 0.1 M ammonium persulfate and 2 M sodium hydroxide for 30 min. In this process, CM was directly used as the Cu precursor, and $Cu(OH)_2$ NRs spontaneously grew on the surface of CM. The obtained $Cu(OH)_2$ NRs electrode was washed with Milli-Q water and dried using an N_2-gun stream.

b. Sputtering of Cu and Ti layers onto the surface of $Cu(OH)_2$ NRs: the obtained $Cu(OH)_2$ NRs electrode was then placed in a sputter coater (Q150T ES PLUS) to first deposit a Cu layer (film thickness monitor -FTM-= 60 nm, tooling factor= 3.4) and then a Ti layer (FTM= 30 nm, tooling factor= 3.4) onto the surface of $Cu(OH)_2$ NRs. The resulting electrode, named Ti@Cu@$Cu(OH)_2$ NR, was partially oxidized upon air exposure, forming surface layers of copper oxides and titanium oxides, leading to the TiO_2@CuO@$Cu(OH)_2$ NRs electrode. When the thickness of layer coatings was indicated, the produced electrode was defined as yTiO_2@xCuO@$Cu(OH)_2$ NRs, where x and y indicate the thicknesses, expressed in nm, of sputtered Cu (CuO) and Ti (TiO_2), respectively.

c. In-situ deposition Ru nanocrystals to produce the Ru@Cu-yTiO_2/xCu (target electrode): the obtained yTiO_2@xCuO@$Cu(OH)_2$ NRs electrode (**Figure S1a)** was cut into the desired size (typical working area: 1 cm^2) and immersed in a 1 M NaOH solution (25 mL). Subsequently, a negative current density of -5mA/cm^2 was applied on a three-electrode cell configuration, using an Ag/AgCl (Sat. KCl) as the reference electrode and a winded Pt wire as the counter electrode (*Note: Although for the evaluation of HER catalysts the use of carbon rod is recommended instead of Pt counter electrode (due to the possible deposition of dissolved Pt onto the working electrode[1]), the carbon oxidation leads to the release of carbon ashes during Ru deposition and performance evaluation at high current density,[2] as indicated in **Figure S34**. Besides, we found that our cathode synthesized using Pt as counter electrode has negligible Pt deposition, as no Pt signal was detected in the produced catalyst by XPS analysis. Check more details in **Figure S34***). Thus, the $Cu(OH)_2$ skeleton was slowly reduced, transforming the oxidized Cu layer into metallic Cu obtaining the TiO_2/Cu electrode. **Figures S1b,c** shows the CP plot, until the electrode potential became stable, indicating the end of the electrochemical reduction protocol. Afterwards, Ru nanocrystals were electrodeposited onto the TiO_2/Cu surface by adding 400 μL of K_2RuCl_6 aqueous solution (1 mg/mL) and by applying a negative potential of -0.2 V (*vs.* RHE). As shown in **Figure S1d**, the chronoamperometric (CA) plot increased upon the Ru precursor addition, indicating the initialization of the Ru deposition (and the evolution of H_2). The fabrication of the electrode (**Figure S1e**) was completed once the CA plot became stable.

Notably, by optimizing the Ru precursor concentration from 2 to 16 μg/mL, we reduced the electrodeposition time from 18 h to 3 h, while increasing the utilization of Ru from its precursor from ca. 30 % to over 60 %, without decreasing the HER performance of the electrode (**Figure S9**).

The optimized electrode prepared following abovementioned steps, namely Ru@Cu-30TiO_2/60Cu, was simply denoted as Ru@Cu-TiO_2/Cu for clarity. Otherwise, the thickness of coating layer was indicated as Ru@Cu-yTiO_2/xCu.

Preparation of control electrodes based on the commercial Pt/C

2 mg of commercial 20 wt% Pt/C (platinum on graphitized carbon, Sigma-Aldrich) was added into a glass vial containing water/isopropanol (0.2/0.18 ml), and then 20 μl of Nafion (5 wt% Nafion 117 containing solution, Sigma-Aldrich) was added into the dispersion. The mixture was sonicated for at least 30 min to obtain a homogeneous ink. 100 μL of obtained ink was then drop-casted onto the substrate of either CM (Pt/C-CM) or CPR (Pt/C-CPR) (corresponding to a Pt loading of 100 μg/cm^2). Before depositing Pt/C, the CPR was treated to make it hydrophilic. More in detail, a Milli-Q water-rinsed CPR was immersed in 3M H_2SO_4 overnight. Such acid-treated CPR was then rinsed with Milli-Q water, and then dried into a glass petri dish loaded on a hot-plate at 60 °C. After Pt/C deposition, the prepared electrodes were dried in air before measurements.

Characterization of electrode materials

XRD measurements were carried out on a PANalytical Empyrean using Cu Kα radiation. SEM images were acquired on a JEOL JSM-6490LA microscope operated at an acceleration voltage of 5-10 kV, while EDS measurements were performed at 20 kV. XPS measurements were performed on a Kratos Axis UltraDLD spectrometer at 20 mA and 15 kV using a monochromatic Al Kα source, while high-resolution analyses were carried out at pass energy of 10 eV. The binding energy scale was referenced to the C 1s peak at 284.8 eV. The spectra were analyzed using CasaXPS software (version 2.3.17). TEM images were obtained with a JEOL JEM-1011 microscope operated at 100 kV. The TEM samples were prepared by detaching the catalyst from the CM substrates through a gentle sonication, obtaining catalyst dispersions in ethanol. Such dispersions were then drop-cast onto ultrathin holey C-coated Cu grids. HAADF-STEM and HRTEM imaging were carried out on an image-Cs-corrected JEOL JEM-2200FS TEM operated at 200 kV and using a direct electron detection camera (Gatan K2 Summit) and a Bruker silicon-drift detector (XFlash 5060). Further HAADF HRSTEM and EDS analyses were carried out on a probe- and image-Cs-corrected ThermoFisher Spectra 300 S/TEM microscope equipped with X-FEG source, ThermoFisher monochromator, a direct electron detection camera (Gatan K3) and Dual-X EDS system. For current observations the microscope was operated at 300kV. The HRTEM and HRSTEM samples were prepared by drop-casting the catalyst dispersions onto holey C-coated Au or Ni grids. ICP-OES measurements were carried out on a ThermoFisher iCAP 6500 Thermo spectrometer. Depending on the requirements, the samples were prepared by dissolving a piece of catalyst electrode (of known size around 0.5 cm^2) in 2.5 mL aqua regia (HCl/HNO_3 3:1, v/v) overnight for digestion. The obtained dispersions were then diluted to 25 mL with Milli-Q water, and ~10 mL of solution was filtered through a 0.45 μm Nylon filter for measurement. The ICP measurements were affected by a systematic error of *ca.* 5 %.

Electrochemical tests of the electrodes

The electrocatalytic activity of the electrodes was evaluated on a conventional three-electrode cell configuration using an Ivium-n-Stat potentiostat. A double-junction Ag/AgCl (Sat. KCl) and a winded Pt wire were used as the reference electrode and the counter electrode, respectively. Note: carbon

rod was not used as counter electrode due to the carbon oxidation leading to the release of carbon ash under high working current density. In addition, several investigations have been carried out to exclude the possible influence of Pt deposition onto the electrode surface (**Figure S34**). A 1 M NaOH solution was used as the electrolyte solution. Potentiodynamic LSV measurements were performed at a scan rate of 2 mV/s and were shown after iR-correction (*i* is the measured working current and R is the series resistance). "Uncompensated resistance (R_u)" was measured through EIS measurements of the cathode at -0.1 V (*vs.* RHE), using a frequency range of 0.1 Hz-100 kHz. A 100 % iR compensation was considered in our work (see detailed discussion in **Figure S20**). The CP and CA plots were displayed without iR-correction. The Tafel slope was used as a metric to assess the HER kinetics of the electrodes. Such a parameter was estimated from the linear portion of the Tafel plot (overpotential *vs.* log(|current density|) curve).[3] Besides, galvanostatic polarization curves were also acquired on electrodes of Ru@Cu-TiO_2/Cu NRs through multistep CP protocol to accurately determine the Tafel slope of the electrodes. The durability of the working electrodes was evaluated through CP measurements. The measured potentials were converted to the RHE scale according to the following Nernst equation:

$$E_{RHE} = E_{obs} + E^{o}_{Ag/AgCl} + 0.0591 \times pH = E_{obs} + 1.02\ V$$

The part of ($E^{o}_{Ag/AgCl}$ + 0.0591×pH) was experimentally measured according to the calibration process described previously.[4] The experimentally measured value of ~1.02 V was close to the theoretical one computed by the Nernst equation: 1.024 V. EIS spectra of Ru@Cu-TiO_2/Cu were acquired at various potentials (50, 0, -10, -25, -50, and -75 mV *vs.* RHE) in a frequency range of 0.01Hz-10 kHz to analyse the HER catalytic kinetics. The EIS spectra of Pt/C made from its corresponding ink deposited onto the glassy carbon electrode (Φ = 3 mm), measured in 1 M H_2SO_4 and 1 M NaOH, were also acquired for comparison.

The **TOF of the Ru@Cu-TiO_2/Cu** was calculated according to:

$$\mathrm{TOF} = \frac{\text{hydrogen molecule number}}{\text{number of active sites}},$$

in which

$$\text{hydrogen molecules number} = \left(\frac{\mathrm{j}}{1000} \times \mathrm{N_A}\right) / (\mathrm{F} \times \mathrm{n}) = 3.12 \times 10^{15}\ |\mathrm{j}|\ \frac{\mathrm{H_2/s}}{\mathrm{cm^2}}\,\text{per}\,\frac{\mathrm{mA}}{\mathrm{cm^2}}$$

being j the current density expressed in mA/cm^2, N_A the Avogadro constant (6.022×10^{23}/mol), F the Faraday constant (96485 C/mol) and n the number of electrons transferred to generate one molecule of the H_2 (*i.e.*, n = 2)

Number of Ru sites (assuming all the electrodeposited Ru to be active)

$$= \left(\frac{\text{mass loading of Ru per geometric area determined by ICP}}{\text{Ru molar weight}}\right) \times \mathrm{N_A}$$

$$= \frac{52\ \times 10^{-6}\ \mathrm{g/cm^2}}{101.1\ \mathrm{g/mol}} \left(\frac{6.022 \times 10^{23}}{1\ \mathrm{mol}}\right) = 3.097\ \times 10^{17}\ (\text{Ru sites})\ \text{per cm}^2$$

Therefore,

$$\mathrm{TOF} = \frac{3.12\ \times 10^{15}}{3.097\ \times 10^{17}}\left|\mathrm{j}\right| = \frac{1.0074\ \left|\mathrm{j}\right|}{\mathrm{s}}\mathrm{per}\frac{\mathrm{mA}}{\mathrm{cm}^2}$$

The ECSA of the electrodes was estimated by performing CV measurements in a non-Faradaic potential window (0.05 V to 0.11 V, *vs.* RHE) at varied scan rates: 4, 8, 12, 16, and 20 mV/s. The electrode double layer capacitance (C_{dl}) was considered equal to the slope of obtained linear fit by plotting the Δj versus the scan rate (ν), with Δj being set as the current density between the cathodic and anodic sweeps at the certain potential of 0.08 V (*vs.* RHE). Then the electrode ECSA was considered proportional to its C_{dl}, in accordance with the following equation:

$$\mathrm{ECSA} = C_{dl}/C_s,$$

where C_s indicates the specific electrochemical double-layer capacitance of an theoretically smooth surface, assumed to be 0.04 mF/cm^2 as reported previously.[5] Note: As discussed in the cited work, the C_s values vary for various materials and for the same material based on testing under alkaline and acidic conditions. Hence, it is difficult to accurately determine the capacitance of a specific catalyst that consists of various species.

The **Faradaic efficiency** of the HER of the cathode was analyzed by measuring the amount of evolved H_2 through a gas chromatograph (SRI instruments), equipped with a HayeSep D porous polymer column, thermal conductivity detector, and flame ionization detector. Ultra-pure N_2 gas (99.999 %), used as the carrier gas, was bubbled inside the cathode side of a well-sealed H-cell, during which the cathode is operating, separated from anode by a Fumasep FAA-3-PK membrane, at a constant current density of -20 mA/cm^2 (CP mode), to continuously monitor the Faradaic efficiency.

The Faradaic efficiency for the HER of the cathode was calculated as:

$$\text{Faradaic efficiency} = \frac{\mathrm{n} \times \mathrm{F} \times \mathrm{C} \times \mathrm{G} \times \mathrm{P}}{\mathrm{R} \times \mathrm{T} \times \mathrm{i}}$$

where n indicates the number of electrons transferred to generate one H_2 molecule (n = 2) , F indicates the Faraday constant (96485 C/mol), C indicates the measured concentration of the product by gas chromatography (in ppm), G indicates the gas flow rate (mL/min), P indicates the working pressure (1.01×10^5 Pa), R indicates the universal gas constant (8.314 J·mol^{-1} K^{-1}), T indicates the room temperature (293.15 K), and i indicates the working current.

X-ray absorption spectroscopy measurements

XAFS measurements were carried out at the 2.4 GeV XAS beamline in the fluorescence mode at Elettra-Sincrotrone Trieste (Italy).[6] A double-crystal Si (111) monochromators was used to monochromatize the beam for the measurements at the Ti K-edge (4,966 eV), Cu K-edge (8,979 eV), and Ru K-edge (22,117 eV). A silicon drift detector was used to analyses the fluorescence intensity. The corresponding benchmark was also measured immediately before/after each sample measurement. For *operando* XAS measurements, the as-prepared electrode was taken as working electrode mounted on the three-electrode cell configuration connected to the Ivium-n-Stat potentiostat, while a homemade polypropylene-type cell containing 1 M NaOH electrolyte was used and mounted at an angle of *ca.* 45° with respect to the incident beam and fluorescence detectors (see in **Figure 4a**). During the *operando* experiments, different potentials, *i.e.*, OCP, -10 mV, and -30 mV (*vs.* RHE), were applied to the working electrode to run the CA measurements. Eight consecutive scans on

Ru K-edge were performed for each step. All the XAS data were processed using the ATHENA program.[7] The Fourier transform (FT) of the EXAFS signals was carried out in the interval 2.5 $Å^{-1} \leq k \leq 10$ $Å^{-1}$. Suitable treatment (Adjacent-averaging method. Points of window: 8) to smooth XAS data was conducted when necessary, and the raw data after normalization was provided in the **Figure S35**.

First-principles simulations

DFT simulations were performed through the Vienna Ab initio Simulation Package (VASP).[8] The Perdew-Burke-Ernzerhof (PBE)[9] exchange correlation functional was used within the framework of the projector augmented-wave (PAW) method.[10] More details are reported in **Note S2**.

The energy of the reactions (2)(5) were respectively calculated through the following equations:

$\Delta E_2 = E_{slab\text{-}H2O} - E_{H2O}$,

$\Delta E_3 = E_{slab\text{-}H} + E_{slab\text{-}OH} - E_{slab\text{-}H2O}$,

$\Delta E_4 = E_{slab\text{-}OH} + ½ E_{H2} - E_{slab} - E_{H2O}$,

$\Delta E_5 = ½ E_{H2} + E_{slab} - E_{slab\text{-}H}$,

in which E_{slab} is the energy of a metal slab (Cu, Ru, or Cu-Ru surface alloy), while $E_{slab\text{-}"fragm"}$ ("fragm"=H, OH or H_2O) is the energy of a metal slab with the "fragm" molecule or fragment adsorbed onto it. Only the most energetically favourable types of adsorption sites were considered, namely hollow-fcc sites for H and OH and atop for water. E_{H2O} and E_{H2} are the energies of isolated H_2O and H_2, respectively. Note that the reaction energy ΔE_4 corresponds to the following reaction:

$OH^* + ½ H_2 \rightarrow * + H_2O$,

rather than to reaction (4) in the main text. However, in **Note S3.2** we demonstrate that, at the electric potential used for HER, the (free) energy change of that reaction is equivalent to that of reaction (4). Note that instead of the Gibbs free energy, whose changes along reactions determine chemical equilibria, we considered only the electronic energy (*i.e.,* the internal energy without vibrational contributions). The latter is the dominant term in the free energy, although other small yet non-negligible contribution exists, mainly the change in solvation and vibrational free energies upon adsorption. However, these two factors are independent of the catalyst adopted, hence they are not relevant for our (mostly qualitative) comparison of reaction energies among Cu-Ru alloys.

Electrochemical tests of the AEL

The AELs were assembled into a zero-gap single electrolysis cell (Dioxide Materials), including corrosion resistant Ni-based anodic and cathodic flow field (bipolar) plates, o-ring seals, and Teflon gasketing. CPR was used as GDL at the cathode side, while no GDL was introduced at the anode side. The cathode was used as-synthesized, while the anode consisted of 5-stacked SSMs. CM, Pt-TPR (used as received), and Pt/C-CPR were also tested as cathodes for benchmark purposes. The Pt/C-CPR cathode was prepared through spray coating of inks of Pt/C in water:isopropanol (3:1 v/v), which were produced with a Pt/C concentration of 1 mg/mL and adding an amount of Nafion dispersion (10 wt%, FuelCell Store) to get a weight content of the ionomer binder of 25 wt% in the final catalyst coatings. The amount of sprayed inks was adjusted to get a Pt mass loading of ~75 $\mu g/cm^2$. Zirfon Perl UTP 220 (*ca.* 220 μm) was used as diaphragm. The cell components were compressed during installation operation to realize a (quasi) zero-gap assembly. The assembled AELs were then connected to a peristaltic pump (Masterflex L/S Series), with a 30 wt% KOH solution continuously supplying the anodic and cathodic half-cells at a flow rate of 30 mL/min per cm^2 of electrode area, at a temperature of 80 °C

(controlled with a proportional-integral-derivative controller) under atmospheric pressure (1 bar). A VMP3 Biologic potentiostat/galvanostat, equipped with an external high current (20 A) booster channel, was used to provide the electrolysis power. LSV, CP, and EIS measurements were carried out with the same potentiostat/galvanostat. Polarization curves were acquired through CP sequences. The cell voltage was monitored over 2 min of each galvanostatic step, until it stabilized to get the data for each point of the polarization curve. The stability of the AEL under continuous operation was assessed through CP measurement at 1 A/cm^2. For the polarization curve, the AEL station operated with separate electrolyte cycles, avoiding mixing of the anodic and cathodic electrolyte cycles of traditional AEL electrolysis, a practice recommended in previous reports.[11] This AEL operation management can limit the anodic hydrogen contamination, guaranteeing a safe operation without requiring extra measures (*e.g.*, gas separating unit) to reduce the crossover or the hydrogen content within the anodic half-cell.[11] Combined cycles were instead using for stability measurement at 1 A/cm^2 current density, far from the unsafe conditions typically occurring at low current density operation.[12] Similar to tests reported for other types of electrolyzers (*e.g.,* PEM electrolyzers),[13] the AST protocol involves cycling between 0.05 A/cm^2 and 1.0 A/cm^2, with each galvanostatic step maintained for 15 min for an overall test duration of 24 h. The energy efficiency of the AELs was calculated as below:

$$\text{energy efficiency} = \frac{E_{\text{output}}}{E_{\text{input}}} = \frac{M_{H2} \times \text{HHV}}{E_{\text{input}}}$$

In the expression above, M_{H2} is the hydrogen gas weight, HHV is the higher heating value of H_2 (141.7 kJ/g H_2), and E_{input} is the electric power consumed to produce the hydrogen. Notably, although this efficiency metric based in HHV are commonly used in literature, the E_{input} neglects some energy contribution input to the electrolyzer, such as and thermal energy input and energy consumption of water peristaltic pumps, leading to energy efficiency even higher than 100% (especially when working at low current densities), which doesn't make sense.[14] To be accurate, voltage efficiency was also used as reliable metric, and calculated as:

$$\text{voltage efficiency} = \frac{\text{Thermodynamic voltage (V)}}{\text{Operating voltage (V)}}$$

where thermodynamic voltage is the ideal voltage to split liquid water under operating conditions (*i.e.*, 80 °C, 1 bar), and could be computed approximately using the following equation:[14]

$$\text{Thermodynamic voltage (V)} = 1.4736 - 0.8212 \times 10^{-3} \times T \text{ (T is expressed in Kelvin)}$$

At 80 °C (and 1 bar pressure), the thermodynamic voltage is 1.184 V.

Calculation details on H_2 production, and its related operation cost are reported on page 66.

Techno-economic analysis

A preliminary TEA was carried out to estimate the CAPEX, the OPEX and the resulting LCOH of 1 MW-scale (net power) AEL plants, ideally up-scaled from our lab-scale results and technology, considering different operative conditions (*i.e.,* different current/voltage settings). All the calculations were performed assuming a complete performance retention from lab-scale tests to plant and a direct and linear proportionality between single cell manufacturing costs and plant scale. The boundaries of the TEA were set at the outlet of the AEL, *i.e.*, hydrogen stocking and transportation costs were not considered.

The unitary cost (US$ per cm^2) of the diaphragm/electrode package (DEP) was calculated considering both the price of the raw materials/components of electrodes and the associate manufacturing costs (**Table S8**).

Starting from data provided by IRENA [15] and reports on currently operating large-scale AEL plants[16] (**Table S9**), the CAPEX of a generic MW-scale AEL was retrieved. Such breakdown was used to compute the total CAPEX of the ideal 1 MW-scale AEL plants based on our DEP technology. The annual CAPEX was then calculated from the total CAPEX considering its depreciation through a capital recovery factor (CRF) (**Table S10**). OPEX-related calculations have been carried out starting from the electrochemical data collected on our lab-scale single cell AEL. In addition to the electrical energy fed to the electrolyzer, other operative expenses, namely process water consumption, labour, maintenance, and other ancillary costs, were considered, (**Table S11**).

The amount of H_2 produced in a year (kg_{H2}/year) by the ideal AEL plants was calculated using the Faraday's law:

$$\text{annual } H_2 \text{ production} = \frac{I \times t \times FE \times MM_{H2}}{n \times F}$$

where I is the total current delivered by the plant in one year, t is the time, FE is the Faradaic efficiency, MM_{H2} is the molecular mass of hydrogen (g/mol), *n* is the number of electrons transferred for each generated H_2 molecule (mol_{e-}/mol_{H2}) and F is the Faraday's constant (C/mol_{e-}^{1}). Note that conversion factors are not displayed in the equation for the sake of clarity.
Finally, the hydrogen production cost was computed as:

$$H_2 \text{ production cost } \left(\text{US\$}/\text{kg}_{H_2}\right) = \frac{\text{Annual CAPEX } + \text{Annual OPEX}}{\text{Annual } H_2 \text{ production}}$$

being the CAPEX equally spreads all over the plant lifetime and the OPEX is fixed (same productivity every year, with the same efficiency), basically the annual cost of H_2 production is the same of the LCOH as no fluctuations of H_2 cost are contemplated by our TEA. According to worldwide averaged data available for large-scale AEL plants,[15,16] which indicate that Balance of Plant power consumption matches that of the actual electrolytic process for plant scales ≥ 1 MW, the annual OPEX comprises a doubling factor for $OPEX_{Electricity}$.

Further details on the assumptions made and parameters set/retrieved from literature throughout the TEA are available in the section of "**Techno-economic analysis**", which also features the Excel spreadsheet used to carry out the analysis.

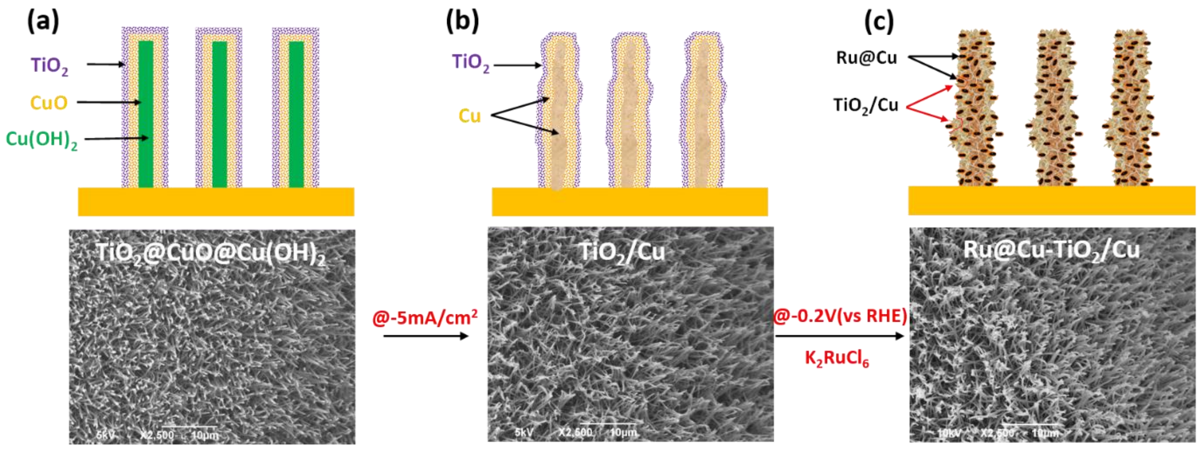

Figure S1. Fabrication procedure of Ru@Cu-TiO_2/Cu. **a**, Sketch and SEM image of the produced $Cu(OH)_2$ NRs on the CM substrate after sputtering Cu and Ti layers consecutively; **b**, sketch and SEM image of reduced TiO_2@CuO@$Cu(OH)_2$ NRs on the CM substrate (TiO_2/Cu); **c**, sketch and SEM image of the target Ru@Cu-TiO_2/Cu. Deposition conditions for Ru: 1 cm^2 electrode, 25 mL electrolyte, 400 μg K_2RuCl_6.

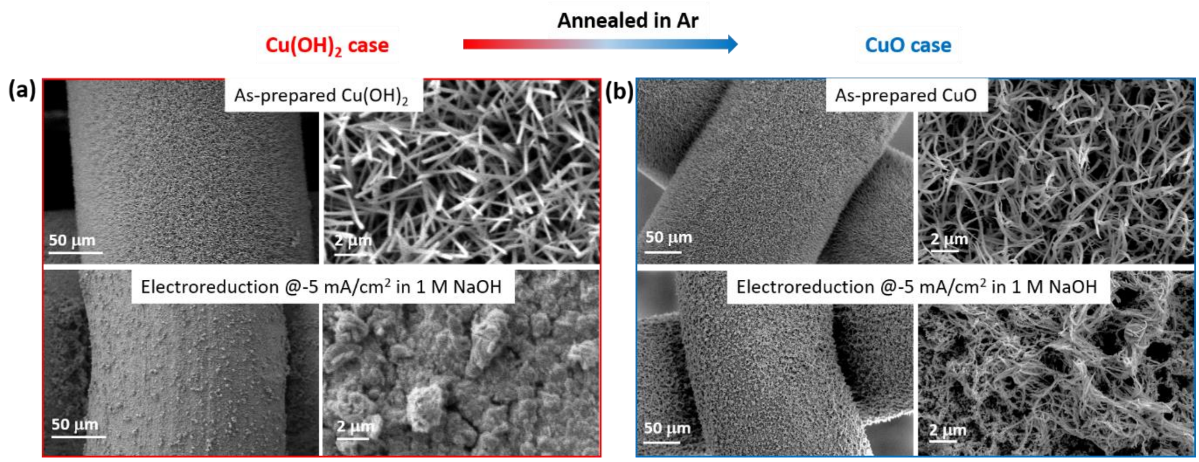

Figure S2. Electroreduction treatment performed on **a,** bare $Cu(OH)_2$ and **b,** its derived CuO nanowire arrays grown on the CM surface, as analyzed through SEM imaging.

The as-prepared $Cu(OH)_2$ NRs array grown on the surface of CM was directly subjected to electroreduction treatment. As shown in **Figure S2**, the 3D-structured NRs were detached from CM as a result of the treatment. This could be due to the difference between the crystal structures of $Cu(OH)_2$ ($Cmc2_1$, a=2.9471, b=10.593, c=5.2564 Å)[17] and reduced Cu (Fm-3m, a=b=c=3.6147 Å),[18] leading to the NRs cracking. Although the destruction of NRs was partially avoided by initializing the electroreduction procedure on CuO obtained from $Cu(OH)_2$ through annealing, the NRs still collapsed leading to interconnected structures, losing the desired vertical alignment of NRs skeleton. This, in turn, decreased the NRs surface area and electrolyte accessibility to the surface of the NRs.

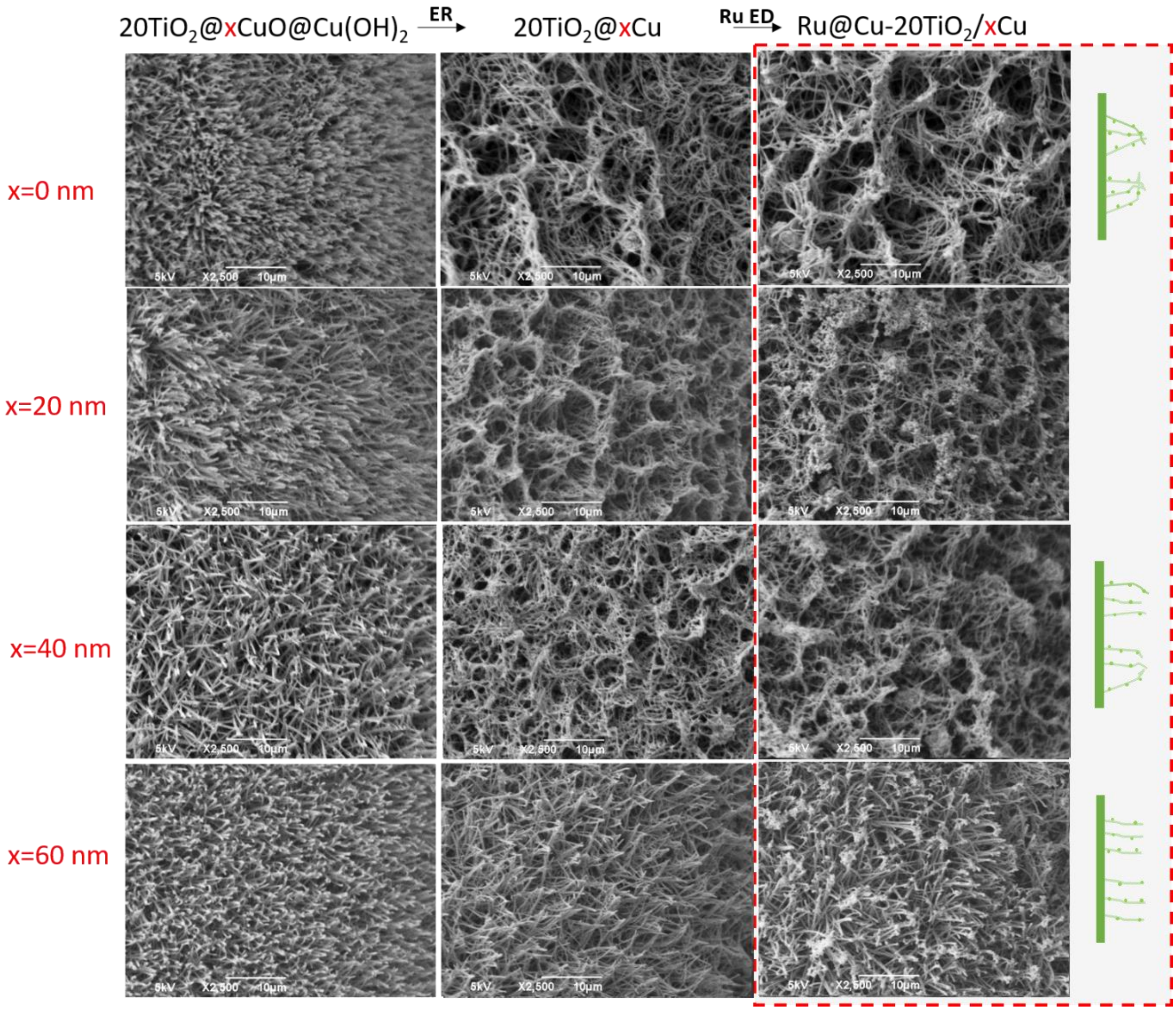

Figure S3. SEM images of investigated electrodes coated with sputtered Cu layers with different thicknesses: 0, 20, 40, and 60 nm. The thickness of sputtered Ti (TiO_2) layer was fixed to 20 nm. The images included in the red-dashed rectangle indicate the final products.

After depositing a thin layer of Ti (*i.e.,* 20 nm), the NRs were retained on the surface of CM during electroreduction treatment and the following electrodeposition of Ru. Nevertheless, the obtained NRs array still lost its vertical orientation. As discussed in the main text, we found that the NRs vertical alignment can be retained by depositing a Cu layer on the surface of $Cu(OH)_2$ NRs. As shown in **Figure S3**, when fixing the thickness of the sputtered Ti layer on the surface to 20 nm, increasing gradually the thickness of sputtered Cu improved the vertical orientation of the resulting Ru@Cu-20TiO_2/xCu. This means that sputtered Cu layer serves as a "protective" layer that maintains the vertical NRs alignment during the electro-synthesis procedure.

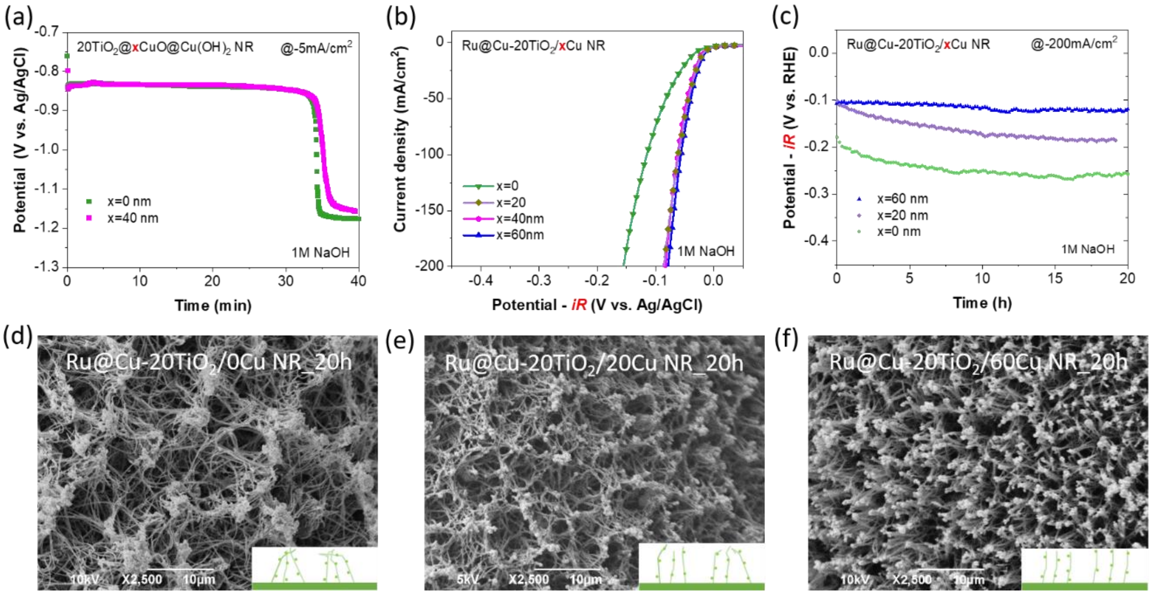

Figure S4. a-c, Performance evaluation of the investigated electrodes coated with sputtered Cu (CuO) layers with different thicknesses; **d-f**, the corresponding SEM images of electrodes after stability test at -200 mA/cm^2 for 20 h. The inset schematics illustrate the orientation of NRs on the CM surface.

Although the vertical orientation of Ru@Cu-20TiO_2/xCu increased with the thickness of the additional Cu (CuO) layer (see discussion in **Figure S3**), the produced electrodes showed similar performances towards the HER in 1 M NaOH for all the Cu layer thicknesses, *e.g.,* Ru@Cu-20TiO_2/0Cu *vs.* Ru@Cu-20TiO_2/60Cu (**Figure S4b**). Nevertheless, the latter distinctly demonstrates a better stability performance towards the HER (**Figure S4b)**, compared to the former case. Hence, 60 nm was selected as the optimal thickness for the sputtered Cu layer in the following study.

Interestingly, although a thin layer (*i.e.,* 20 nm) of Cu coating didn't alleviate the collapse of NRs during the electrosynthesis procedure (**Figure S3)**, the Ru@Cu-20TiO_2/20Cu demonstrated superior HER performance compared to the one obtained without Cu layer coating (Ru@Cu-20TiO_2/0Cu) (**Figure S4b**). We speculate that the sputtered Cu layer, being highly porous CuO (see **Figure 2c** in the main text, and **Figure S11d**), can facilitate the subsequent formation of Ru-Cu nanoheterostructures during the Ru electrodeposition (its presence has been proven in main text, section: ***Synthesis and characterization of the electrode catalysts***), accelerating the overall HER kinetics.

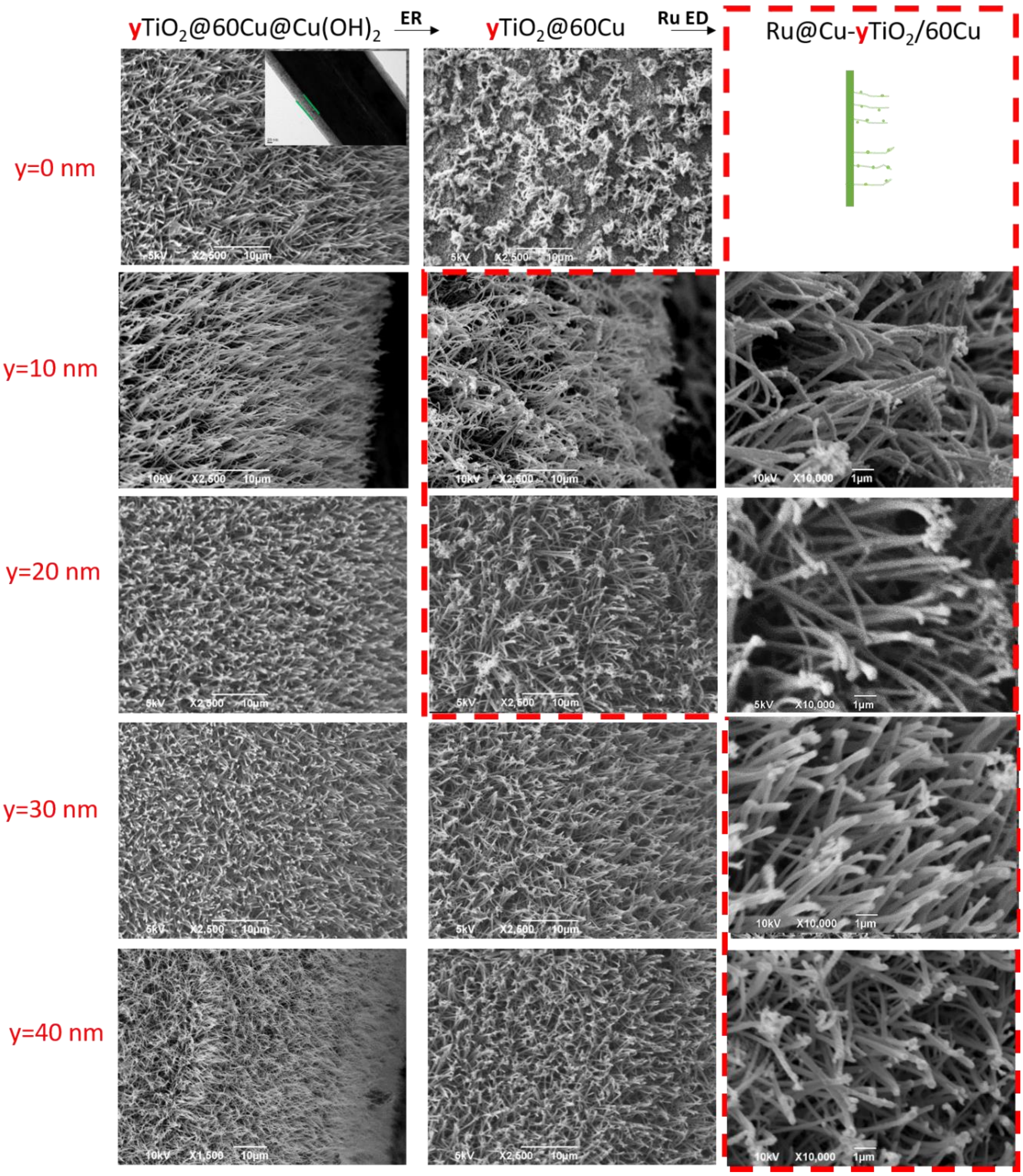

Figure S5. SEM images of the investigated electrodes coated with sputtered Ti (TiO_2) layers with different thicknesses: 0, 10, 20, 30, and 40 nm. The thickness of sputtered Cu (CuO) layer is fixed to 60 nm. The images included in the red-dashed region indicate the final products.

As mentioned above, the optimal thickness of sputtered Cu layer was found to be 60 nm, which was then used to optimize the thickness of the sputtered Ti (TiO_2) layer. As shown in **Figure S5**, without the incorporation of a TiO_2 layer, the 60Cu@$Cu(OH)_2$ NRs array was completely destroyed after the electroreduction step. This is consistent with previous observations (see **Figure S2)**, where the bare $Cu(OH)_2$ and CuO NRs were unstable during the same electroreduction treatment. Interestingly, the incorporation of 10 nm sputtered TiO_2 on the surface of 60Cu@$Cu(OH)_2$ NRs could already maintain the vertical orientation of NR arrays during the entire electrosynthesis process, while the thickest TiO_2 layer did not have an obvious effect on the structure/alignment of the NRs in the final electrode.

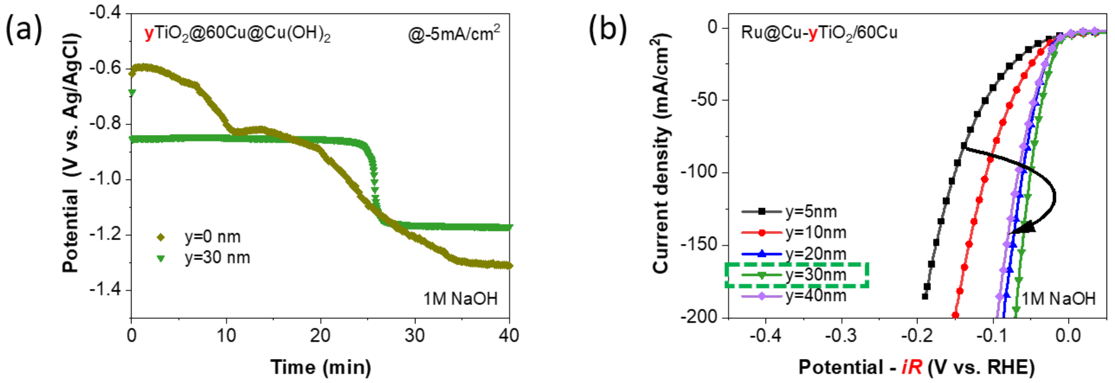

Figure S6. a, Electroreduction plot of yTiO_2@60Cu@$Cu(OH)_2$ NRs electrode using chronopotentiometric technique. **b**, LSV curves measured for the Ru-yTiO_2/60Cu in 1 M NaOH. The thickness of the TiO_2 layer (y) was varied from 0 to 40 nm.

It has been previously proposed that TiO_2 facilitates the water dissociation,[19] improving the overall electrode activity for alkaline HER. Nevertheless, TiO_2 is an electrical insulator and, thus, the optimal amount of TiO_2 must be identified.

As demonstrated in **Figure S6a**, no clear potential plateau was observed on the electroreduction plot of 60Cu@$Cu(OH)_2$ NRs. This behavior could be ascribed to persisting detachment of NRs during their electroreduction, leading to uncontrolled process. In contrast, the 30TiO_2@60Cu@$Cu(OH)_2$ NRs displayed two obvious plateaus, associated to the reduction of the initial $Cu(OH)_2$ and the final Cu phases, respectively. Therefore, the presence of TiO_2 on the surface is crucial to maintain the structure of NRs during our electrosynthesis procedure (see also **Figure S5**). The thickness of sputtered Ti (TiO_2) layer did not affect the structure of the investigated electrodes. However, the thickness of TiO_2 layer significantly influenced the HER performance of the electrodes. The optimal TiO_2 layer thickness was found to be 30 nm (**Figure S6b)**, corresponding to *ca.* 31 μg/cm^2 Ti, as determined through ICP analysis (**Table S1**). The optimized electrodes, namely Ru@Cu-30TiO_2/60Cu, simply labelled as Ru@Cu-TiO_2/Cu, delivered -200 mA/cm^2 current density at an overpotential as low as 74 mV.

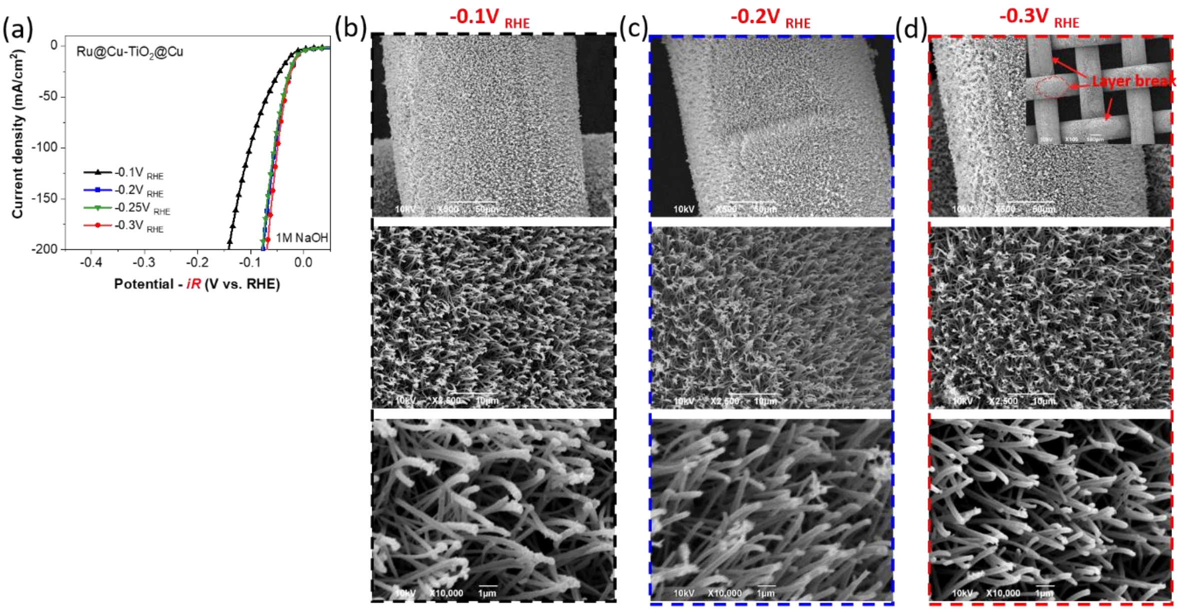

Figure S7. a, LSV curves with iR-correction and **b-d**, the corresponding SEM images measured for Ru@Cu-TiO_2/Cu produced by electrodepositing Ru nanocrystals under different potentials: -0.1 V, -0.2 V, -0.25 V, and -0.3 V (*vs.* RHE).

The electrodeposition of Ru nanocrystals was carried out under different potentials (-0.1 V, -0.2 V, -0.25 V, and -0.3 V *vs.* RHE, CA protocol). The HER activity of the resulting Ru@Cu-TiO_2/Cu was evaluated by recording their LSV curves (**Figure S7a**), concluding that -0.2 V (*vs.* RHE) is the optimal electrodeposition potential. By decreasing the potential to -0.3 V (RHE), the HER activity of resulting electrode slightly increased compared to the other ones, but the catalyst layer started exhibiting cracks in some areas (inset in **Figure S7d**).

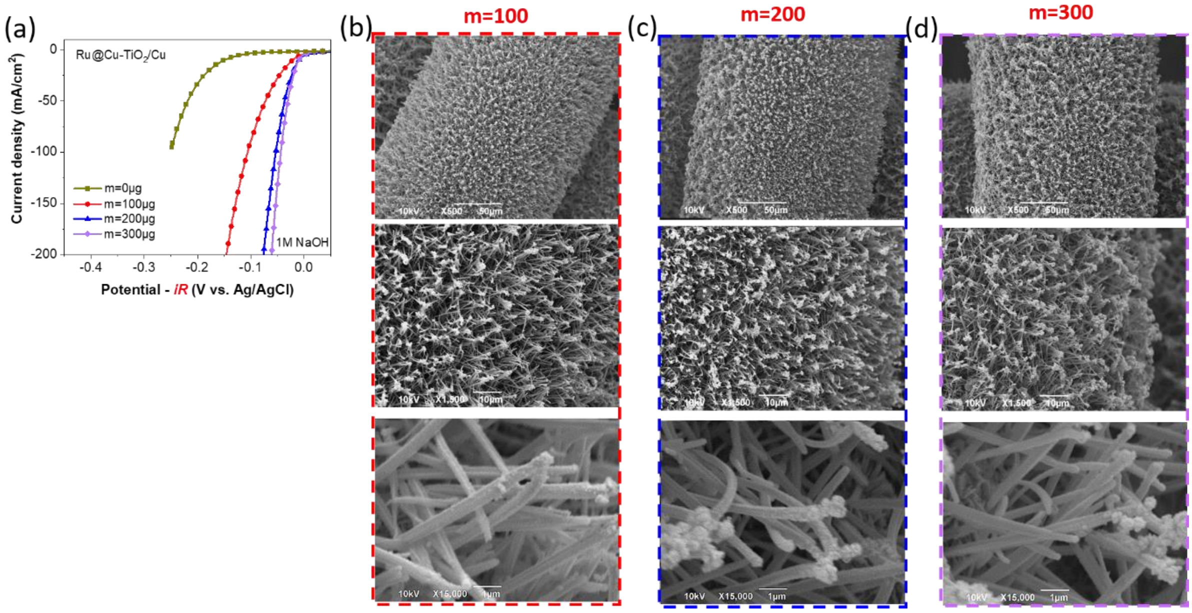

Figure S8. a, LSV curves with iR-correction and **b-d**, the corresponding SEM images measured for Ru@Cu-TiO_2/Cu produced by electrodepositing Ru nanocrystals under different Ru precursor dosage: 100, 200, and 300 μg. Ru nanocrystals deposition condition: 0.25 cm^2 electrode area, 50 mL electrolyte volume, -0.2 V (*vs.* RHE).

The HER performance of Ru@Cu-TiO_2/Cu increased by increasing the amount of Ru precursor (K_2RuCl_6, 25.7 % Ru content) in the electroreduction bath from 100 μg to 200 μg (for the fabrication of 0.25 cm^2 electrodes). However, no obvious activity enhancement was observed by increasing further the amount of Ru precursor to 300 μg, indicating that 200 μg is the optimal dosage of Ru precursor for the Ru electrodeposition step (**Figure S8**).

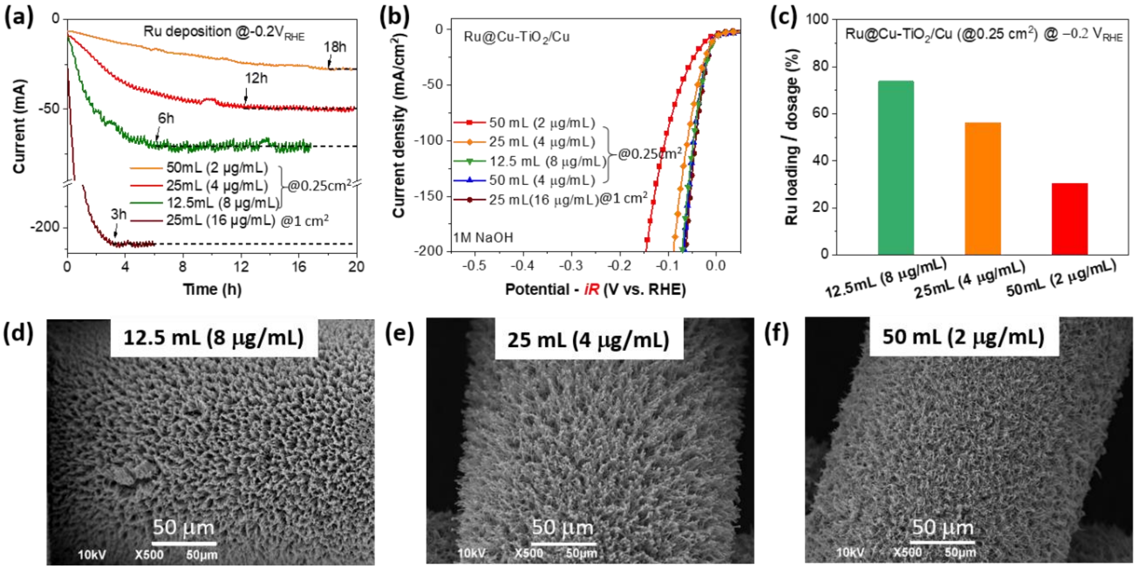

Figure S9. a, CA plots acquired during Ru deposition on Cu-TiO_2/Cu. The stable current plateau indicates the completion of Ru electrodeposition step; **b**, LSV curves with iR-correction measured for Ru@Cu-TiO_2/Cu produced using different concentrations of Ru precursor in different volumes of 1 M NaOH electrochemical bath; **c**, Utilization percent of Ru precursor when fixing its absolute dosage to 100 μg, increasing its concentration from 2 to 8 μg/mL. Electrode size: 0.25cm^2; **d-f**, SEM images of the investigated electrodes, as listed in (c).

Apart from the absolute amount of Ru precursor, the electrodeposition procedure for Ru nanocrystals in 1 M NaOH was also optimized by properly adjusting the concentration of the Ru precursor in the electrochemical bath. For 0.25 cm^2 electrode, increasing the Ru precursor (K_2RuCl_6) concentration from 2 μg/mL (50 mL electrolyte) to 8 μg/mL (12.5 mL electrolyte) shortened the deposition duration of Ru nanocrystals from 18 h to 6 h (**Figure S9a**). Due to the limitation of the three-electrode cell configuration, it was not possible to decrease further the electrolyte volume to increase further the Ru concentration. Nevertheless, such limitation was eliminated by increasing the target size of electrode from 0.25 cm^2 to 1 cm^2, without increasing the electrolyte volume by the same scale. Thus, the electrodeposition of Ru nanocrystals was completed within only 3 h when proceeded in electrolyte containing 16 μg/mL Ru precursor (**Figure S9a**). Besides, although the same amount of Ru precursor (100 μg K_2RuCl_6) were added, the electrode produced in the presence of higher concentration of Ru precursor displayed significantly higher activity towards H_2 evolution, *e.g.,* 50 mL of 2 μg /mL *vs.* 12.5 mL of 8 μg /mL (**Figure S9b**). Higher Ru precursor dosage was required to produce electrode with similar HER activity if a lower concentration or Ru precursor was applied. *e.g.,* 50 mL of 4 μg /mL *vs.* 12.5 mL of 8 μg /mL (**Figure S9b**).

To understand the reason behind the "*higher Ru concentration used for Ru deposition leads to a better performance of the electrode*", the mass loading of Ru within Ru@Cu-TiO_2/Cu was measured using ICP technique. As shown in **Figure S9c**, the highest concentration of Ru precursor resulted in the highest utilization of the Ru precursor (*i.e.,* higher Ru loading), therefore, the corresponding electrode displayed the best performance. **Figures S9d-f** demonstrated that the concentration of Ru precursor did not affect the structure of produced electrode.

In view of an industrial manufacturing of Ru@Cu-TiO_2/Cu cathodes, the optimization of the above-demonstrated protocol focusing at increasing the concentration of Ru precursor is beneficial in terms of both production rate and cost.

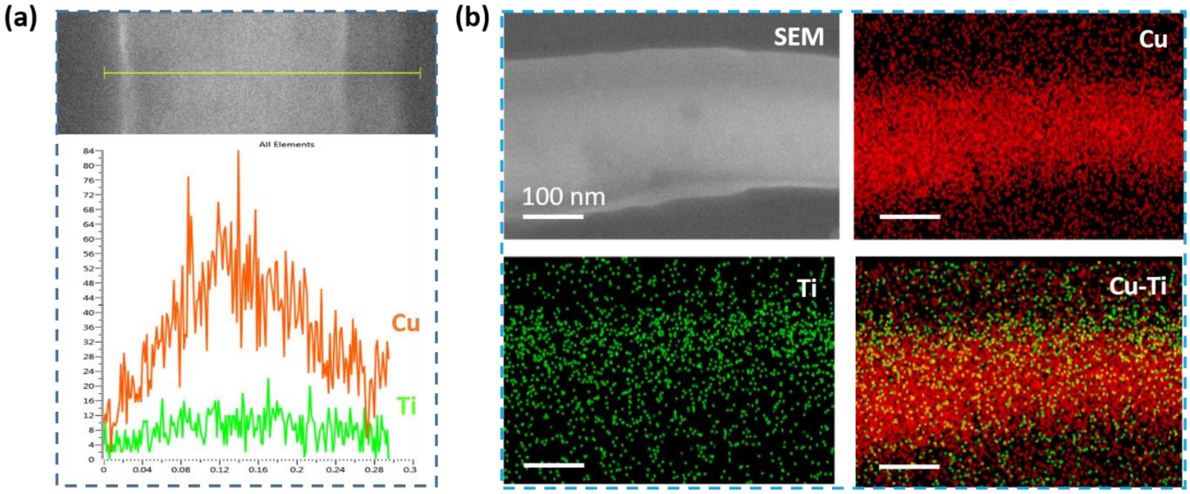

Figure S10. SEM-EDS data recorded from a $30TiO_2@60CuO@Cu(OH)_2$ NR. **a**, EDS line-scan across a single NR; **b**, SEM images and the corresponding EDS maps for Ci, Ti and their superposition, acquired on a single NR. The mapping indicates that Ti is mainly located on the surface of the NR.

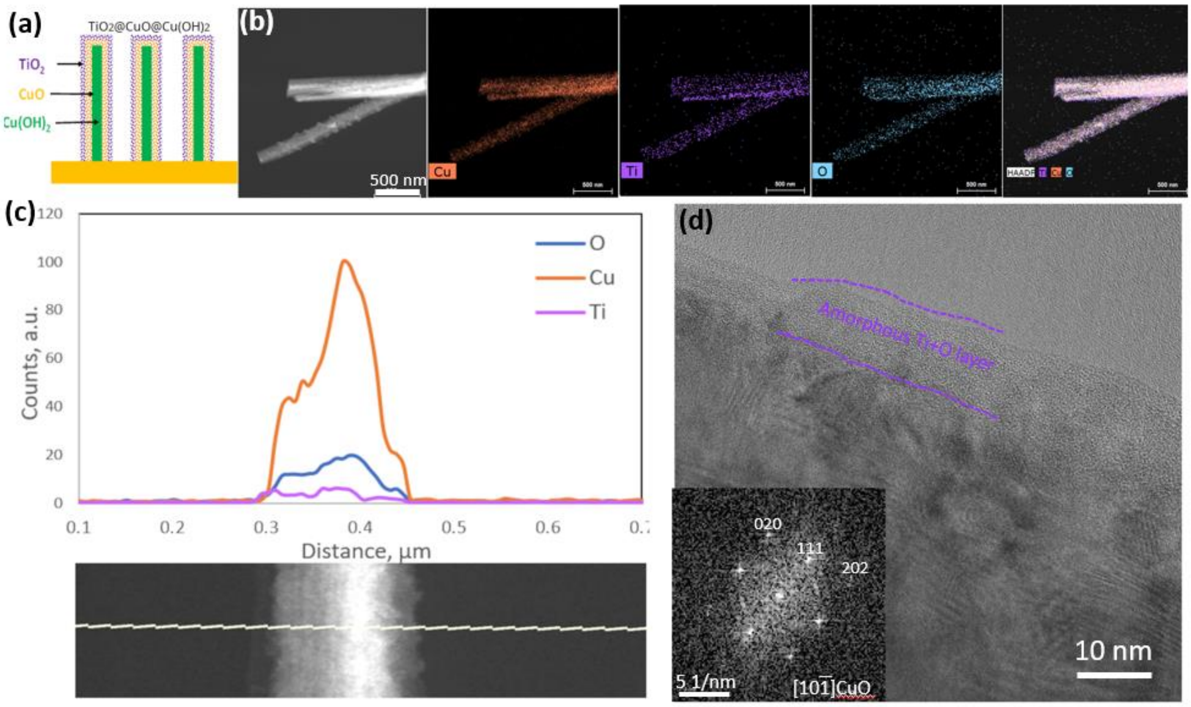

Figure S11. TEM characterization of $30TiO_2@60CuO@Cu(OH)_2$ NRs collected from the CM substrate. **a**, Scheme of the sample along with **b**, HAADF STEM image and the corresponding Cu, Ti and O EDS elemental maps. **c**, An EDS line scan profile across one of the NRs indicating segregation of Ti on the NR surface and strong Cu and O signals coming from the interior of the NR. **d**, HRTEM image from the surface region of one of the NRs with an amorphous layer exposed on the surface, corresponding to Ti -and O-rich layer identified by EDS, and a crystalline core enriched in Cu and O. The structural characterization of subsurface region confirmed the presence of polycrystalline CuO (PDF card 01-089-2531). The FFT of HRTEM image of one of these crystals (inset in **d**) indicates its respective crystallographic orientation.

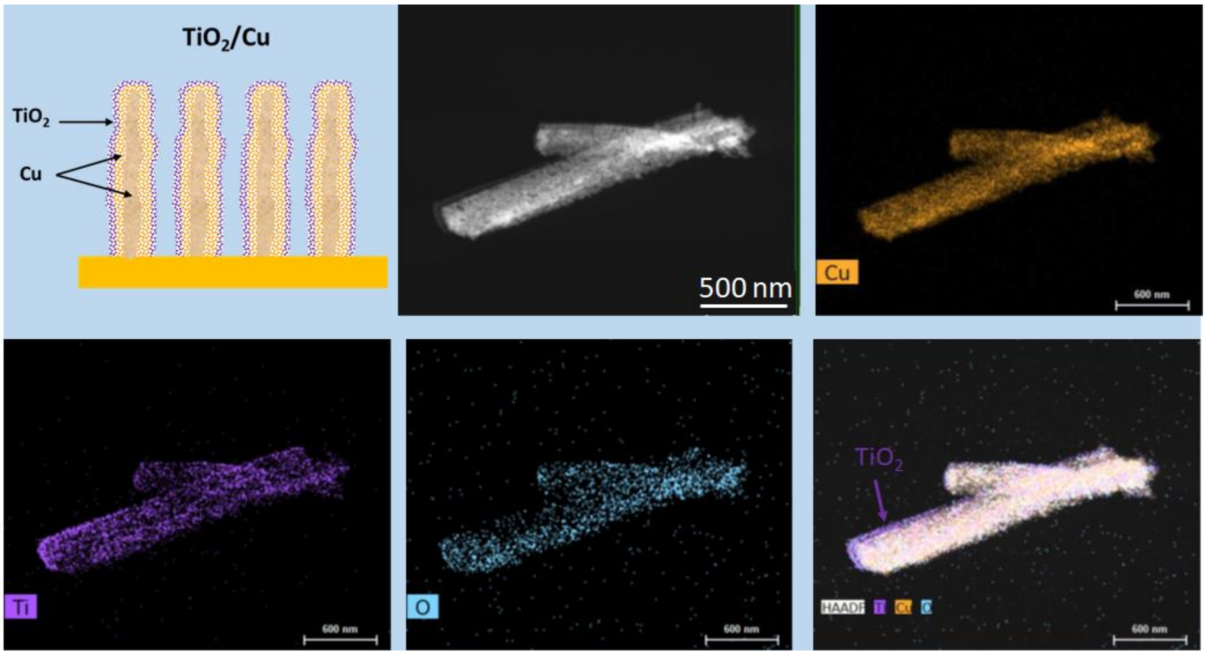

Figure S12. Sketch of the TiO_2/Cu (upper left corner) along with the HAADF STEM image and the corresponding Cu, Ti and O EDS elemental maps from a representative TiO_2/Cu NR collected from the CM substrate.

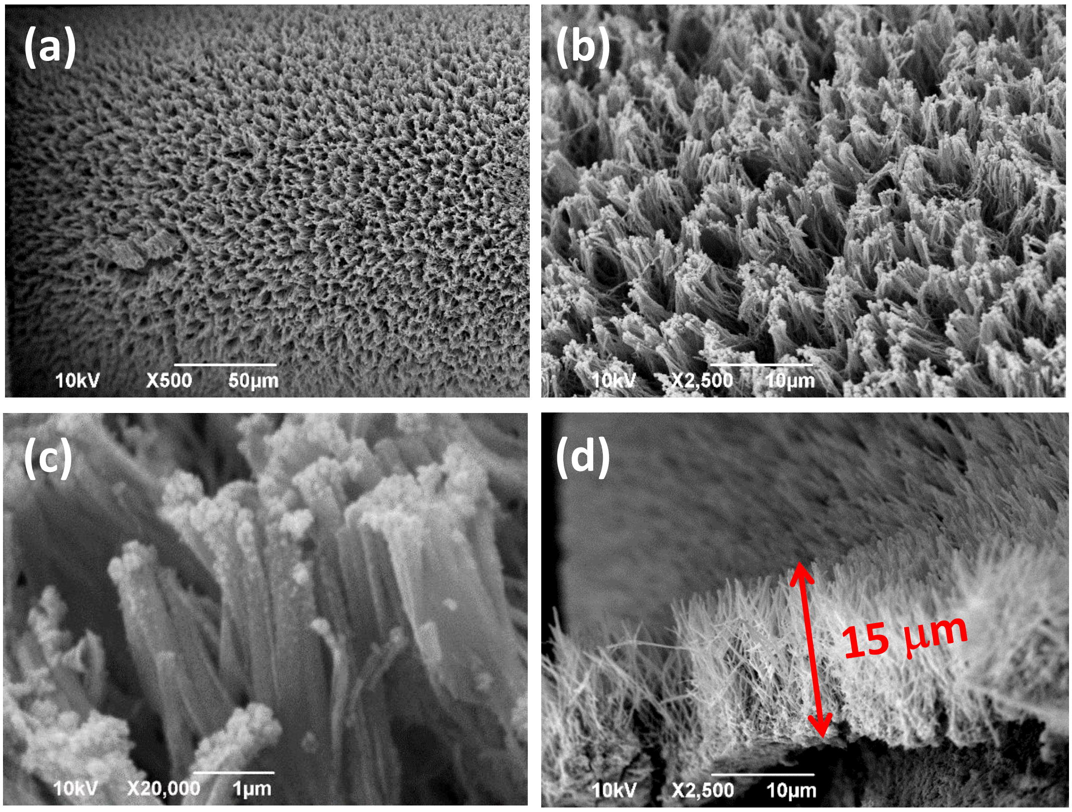

Figure S13. a-c, SEM images of representative NRs in Ru@Cu-TiO_2/Cu, at increasing magnification. **d**, SEM image of tilted Ru@Cu-TiO_2/Cu, showing the thickness of the catalyst layer (*ca*. 15 μm).

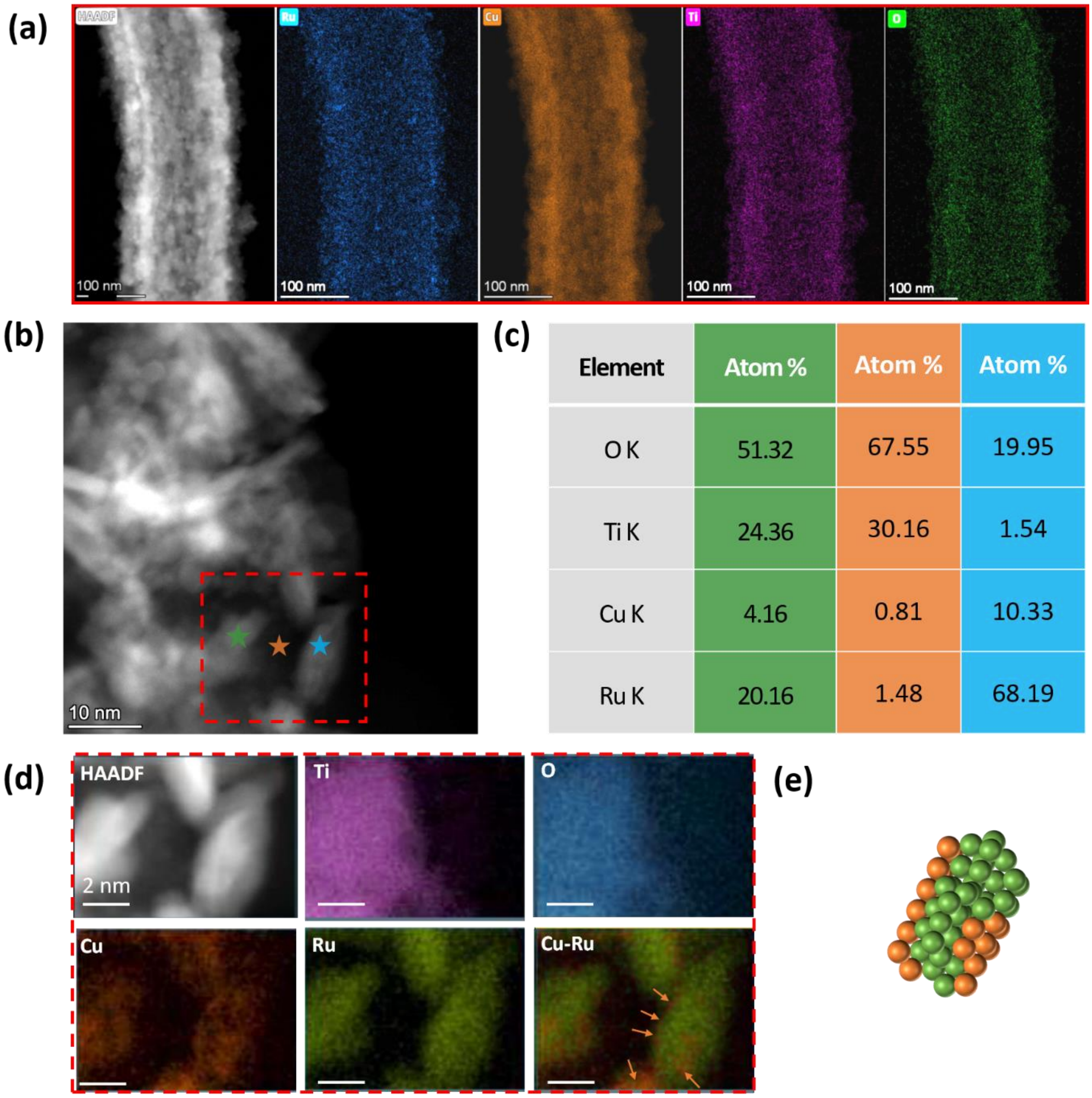

Element	Atom %	Atom %	Atom %
O K	51.32	67.55	19.95
Ti K	24.36	30.16	1.54
Cu K	4.16	0.81	10.33
Ru K	20.16	1.48	68.19

Figure S14. Additional TEM characterization of the Ru@Cu-TiO_2/Cu. **a**, HAADF STEM image and the corresponding EDS maps recorded on a segment of a single NR, revealing the porous nature of the NR and the distribution of all the elements within the NR. **b**, HAADF STEM image of the surface region of one of the NRs with the composition of nanocrystals marked by stars as determined by EDS given in table **c**. **d** Detailed Ti, O, Cu and Ru EDS elemental maps from the area for which the composition is given in table **c** indicating that elongated features, known to be side-oriented Ru nanocrystals, are covered with very thin Cu clusters and similar low-dimensional Cu structures. **e**, a sketch shows the hypothetical arrangement of Cu (orange) and Ru (green) atoms.

(a)

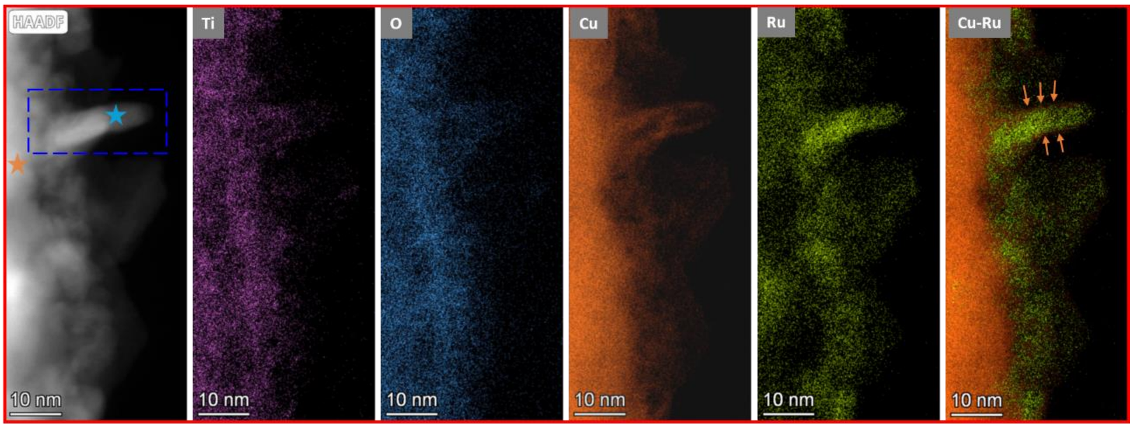

(b)

Element	Atom %	Atom %
O K	15.41	11.08
Ti K	3.43	0.75
**Cu K**	**71.07**	**21.46**
**Ru K**	**10.09**	**66.71**

Figure S15. a, Additional TEM characterization on a single NR in Ru@Cu-TiO_2/Cu, collected from the CM substrate. HAADF TEM image and corresponding EDS maps recorded on one side of a single NR. **b**, Quantitative EDS data obtained from areas indicated by stars in **a**, corresponding to Ru nanocrystal (blue star) protruding from Cu-based core of the NR (orange star).

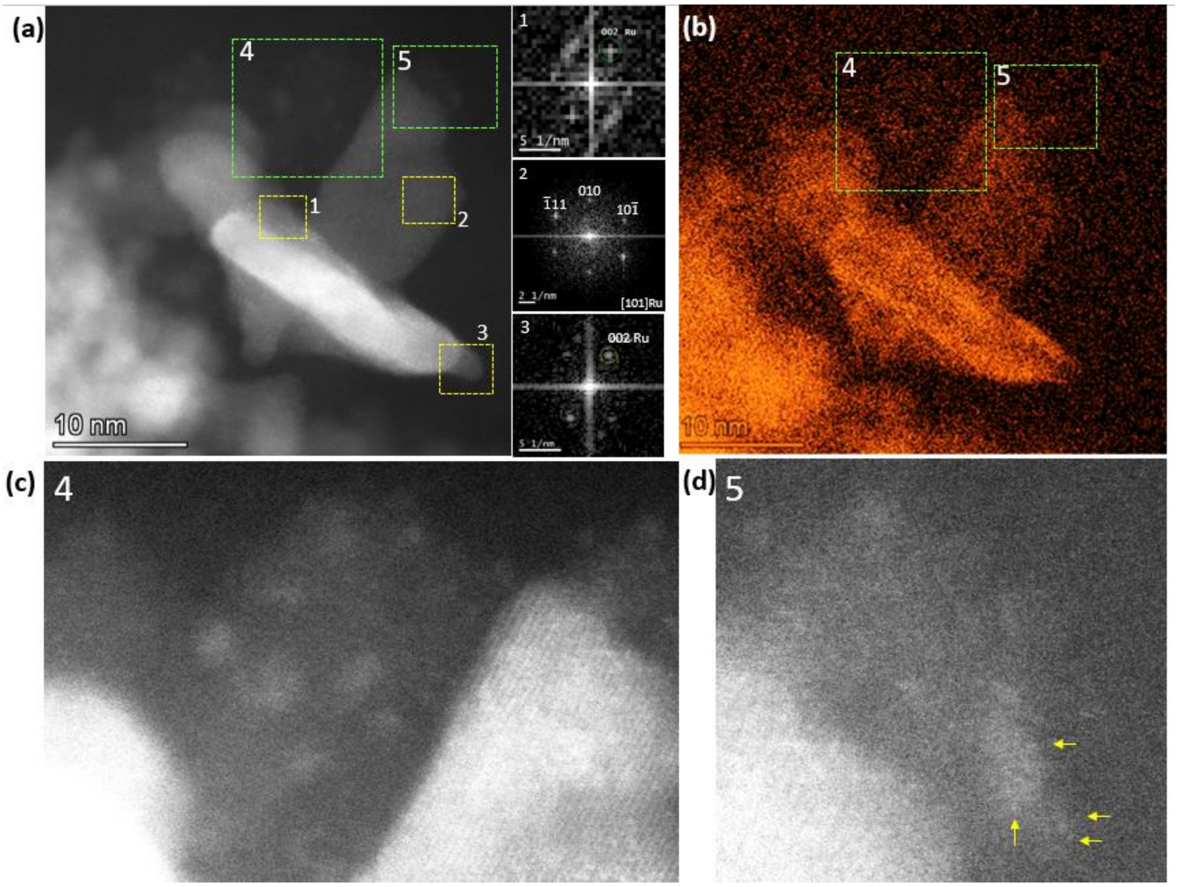

Figure S16. HRSTEM and EDS characterization of the surface of one NR of the Ru@Cu-TiO_2/Cu. **a**, HAADF HRSTEM image, presented also in main text in **Figure 1**, with the FFTs corresponding to areas marked on image as 1 to 3, which confirm the structure of the side-oriented nanocrystals to be that of the crystalline Ru. Rectangles marked 4 and 5 outline areas on the Ru nanocrystals and surrounding TiO_2 that exhibit additional high-contrast features corresponding to atomic clusters of 1 nm or less in size, confirmed to be rich in Cu only based on Cu EDS map in **b**. The same areas are shown enlarged in **c** and **d** with some of the individual atoms being arrowed in **d**. The high contrast of these clusters in HAADF STEM as compared to surrounding oxide and C support, comparable to high contrast of the Ru nanocrystals, also confirms that these are atoms with higher atomic weight and EDS maps indicate that these are Cu atoms.

Discussion on Ru-Cu miscibility and possible alloying

Considering the numerous misconceptions on Ru-Cu alloying that might be found in literature, especially when it comes to nanoparticles, here we try to shed light on the literature claims and, by extension, on the nature of our catalysts.

Literature review on Ru-Cu alloys

First and foremost, it has to be acknowledged that Ru and Cu do not share the same crystal structure, with Ru exhibiting a hexagonal close-packed (*hcp*) structure in the bulk, whereas Cu has a face-centered cubic (*fcc*) structure.[20] It is therefore counterintuitive to envisage alloying between the two elements. Indeed, there is no solid phase miscibility between Ru and Cu according to the ASM (American Society for Metals) Handbook of phase diagrams[21,22] and other reference metallurgy books[23]. Several papers in the literature agree with this statement, reporting: "*The observed lack of metallurgical interactions between Cu and Ru corroborates well with the reported Cu/Ru binary phase diagram according to which the solid solubility of Cu in Ru is negligible up to 900°C*".[24]

Nonetheless, there are also articles that claim a successful alloying of Ru-Cu, mainly at the nanoscale and most likely under kinetic control. It is important to point out that the authors of those papers applied specific synthetic techniques to overcome the Ru-Cu immiscibility. For example, the controlled dealloying of the ternary $Ru_3Cu_{22}Mn_{75}$ alloy[25] or the use of specific precursors like $Ru(acac)_3$,[20] explicitly for "*its ability to form fcc structured Ru NPs*", previously reported by Kusada et al..[26] Therefore, in the absence of such specific precautions, Ru and Cu are envisaged to remain immiscible under 900°C.

Moving to the techniques employed for assessing the formation of Ru-Cu alloys, the main claims reported by the above-mentioned papers rely primarily on XRD and XAS results. Consistently with their effort to obtain face-centered cubic Ru, Huang et al. reported XRD patterns that support alloying (Figure 2 in Ref. 20) as diffraction peaks are consistent with those fcc-Ru NPs and bulk Cu.

On the other hand, Wu et al. used XAS (and specifically EXAFS) to investigate the chemical neighboring of Cu on their Ru-Cu samples. According to the FT k^3-edge weighted c(k)-function of the EXAFS spectra for the Cu K-edge they reported (Figure 1g in Ref. 27), Ru-Cu alloys present a peculiar Ru-Cu distance of *ca*. 2.28 Å. However, it is worth noticing that the reference Cu foil they analyzed in comparison exhibits a peak at ca. 2.22 Å; the authors indeed prudently indexed the peak at 2.28 Å of Ru-Cu alloys as indicative of the Cu-Cu/Ru distance.

Other groups have used EXAFS to prove Ru-Cu alloying. For example, Chen et al. reported the FT k^3-edge weighted c(k)-function of the EXAFS spectra for the Ru K-edge, again discussing shifts of less than 0.1 Å on the bond distance (Figure 3d in Ref. 28). Even in this case, the authors carefully indexed the peak of the alloy as "Ru-Cu or Ru-Ru" distance.

A more convincing use of EXAFS data has been made by Huang et al., who reported for their Ru-Cu a double peak on the FT k^2 -weighted χ(k)-function of the EXAFS spectra for Ru K-edge (Figure 2d in Ref. 29). This is in stark contrast with the single peak they obtained on a reference Ru foil and therefore supports the presence of Ru-Cu bonds.

Despite the effort of these three different research groups, the difference between the distance of the Ru-Ru bond and that of the alleged Ru-Cu remain < 0.1 Å. With these premises, it is not possible to ascertain the presence of Ru-Cu bonding by EXAFS.

Ru-Cu nanoheterostructures reported in this work

The XRD patterns, reported below in **Figure S17a**, do not exhibit any Ru (nor Ti) typical peaks, most likely because of the limited mass loading and/or the small particles size (see comments under the above-mentioned figure).

Regarding instead EXAFS data, we observed two peaks in the FT spectra of Ru k-edge (**Figure 2c** in the main text). Both of them can be easily assigned to Ru-O (*ca*. 1.5 Å) and Ru-Ru (*ca*. 2.4 Å) bond lengths, as extensively reported in the literature.[30] Noticeably, these contributions are also consistent with those same papers that speculated on the formation of a Ru-Cu alloy.[27–29,31]

To conclude, in the light of: (*i*) the metallurgical evidences of Ru-Cu immiscibility; (*ii*) the absence of any precaution taken to prepare Ru-Cu alloying in our syntheses; (*iii*) the unclear possibility to effectively assess the achievement of Ru-Cu bonding by sophisticated techniques such as EXAFS; (*iv*) the absence, in both of our XRD patterns and FT-EXAFS spectra, of any peak or feature ascribable to Ru-Cu bonding, we confirm that there is no any substantial evidence of Ru-Cu alloying in our samples.

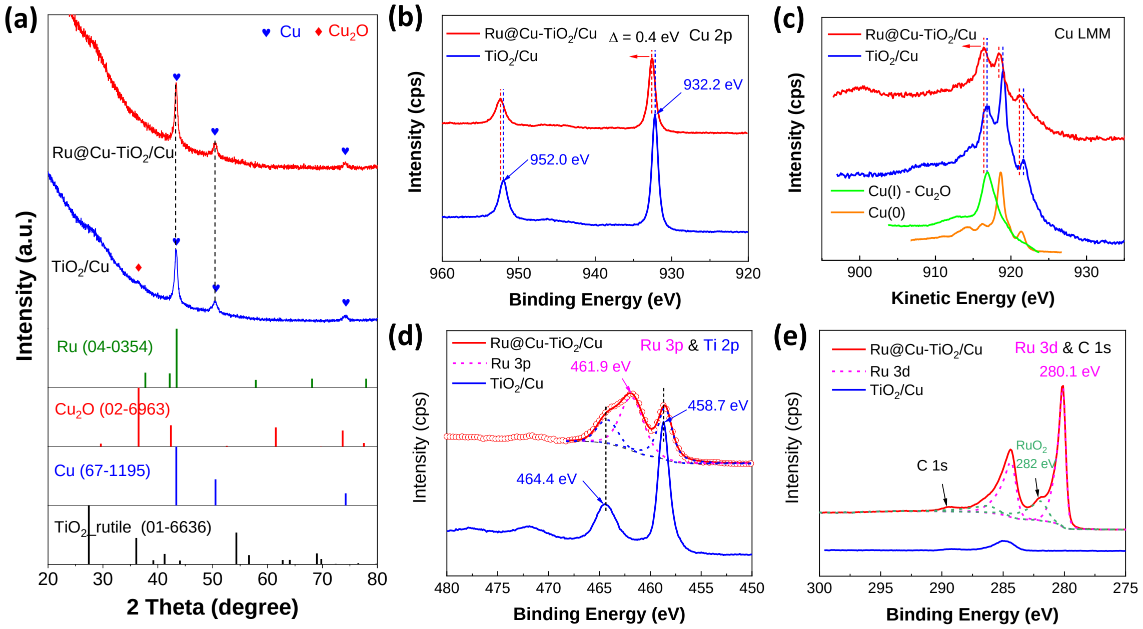

Figure S17. **a**, XRD pattern of the powders of Ru@Cu-TiO_2/Cu, as collected from the as-prepared electrode. The XRD pattern of TiO_2/Cu powder is also shown for comparison, together with the standard XRD patterns of TiO_2 (rutile), Cu, Cu_2O and Ru. XPS analysis of the as-prepared Ru@Cu-TiO_2/Cu and TiO_2/Cu electrodes acquired over the energy ranges for **b**, Cu 2p, **c**, Cu LMM, **d**, Ru 3p (overlapping with Ti 2p), and **e**, Ru 3d (overlapping with C 1s).

X-ray diffraction analysis was performed on TiO_2/Cu before and after the Ru electrodeposition. As shown in **Figure S17a**, the XRD patterns of both electrodes are consistent with the presence of metallic Cu and Cu_2O (originated by the oxidation of the electrode upon air exposure), while TiO_2 and Ru were not detected, most likely due to the poor crystallinity of the former and/or the negligible amount of both species. In fact, inductively coupled plasma optical emission spectroscopy (ICP-OES) analysis revealed that the Ru amount in the Ru@Cu-TiO_2/Cu was ~52 $\mu g/cm^2$, while the Ti amount was ~31 $\mu g/cm^2$. X-ray photoelectron spectroscopy (XPS) measurements were carried out to determine the oxidation state and chemical environment of electrode surface species. The Cu 2p signals (**Figure S17b**) of the TiO_2/Cu electrode indicate that Cu is mainly present in the 0 oxidation state, while the Cu LMM spectrum (**Figure S17c**) indicates a minor presence of Cu(I) species along with Cu(0). Cu(I) species are likely formed upon air exposure, in agreement with the XRD data. Notably, after the electrodeposition of Ru, the Cu 2p peaks shift to higher binding energies, while the Cu LMM peaks shifted to lower ones, indicating an increase in the charge state of Cu, ascribable to electrons transfer from Cu to Ru.[25,29] The Ti 2p peak (**Figure S17d**), located at 458.7 eV, reveals that Ti is in the +4 oxidation state.[32] Notably, no evident peak shift is observed on Ti 2p after the Ru deposition, excluding relevant interactions between TiO_2 and Ru. Both the Ru 3p (**Figure S17d**) and Ru 3d signals indicate that the oxidation state of Ru is mainly 0 with a minor presence of RuO_2 species (**Figure S17e**).

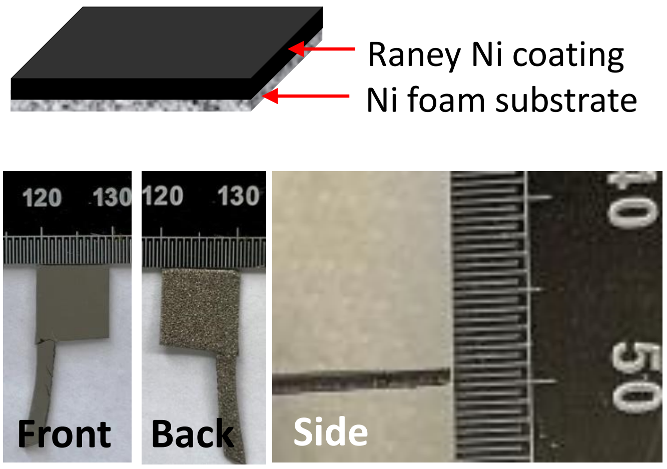

Figure S18. Sketch and photos of the investigated commercial Raney Ni electrode purchased from Fuel Cell Store.

We tested a Raney Ni electrode used in a commercially available lab-scale AEL prototype supplied by Fuel Cell Store.[33] An "L" shaped electrode with the working area of 1 cm^2 was cut for our test (**Figure S18**). The measurement conditions were the same as the Ru@Cu-TiO_2/Cu case. The mass loading of the Raney Ni is 150 mg/cm^2 based on our measurements of the weight of the catalysts layer broken away from the Ni foam substrate.

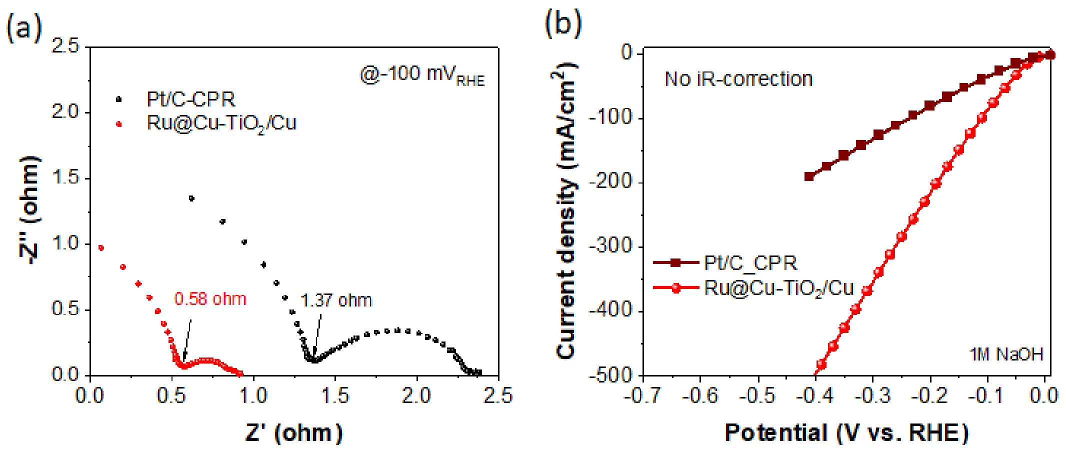

Figure S19. a, EIS plots of indicated electrodes. **b**, Non-iR corrected LSV curves measured for Ru@Cu-TiO_2/Cu and Pt/C-CPR (100 $\mu g_{Pt}/cm^2$). Electrode geometric area = 1 cm^2.

As shown in **Figure S19a**, the "uncompensated resistance (R_u)" of Ru@Cu-TiO_2/Cu and Pt/C-CPR electrodes (whose performances are depicted in **Figure 3a** of the manuscript), determined hin the high frequency region, is 0.58 ohm and 1.37 ohm, respectively.

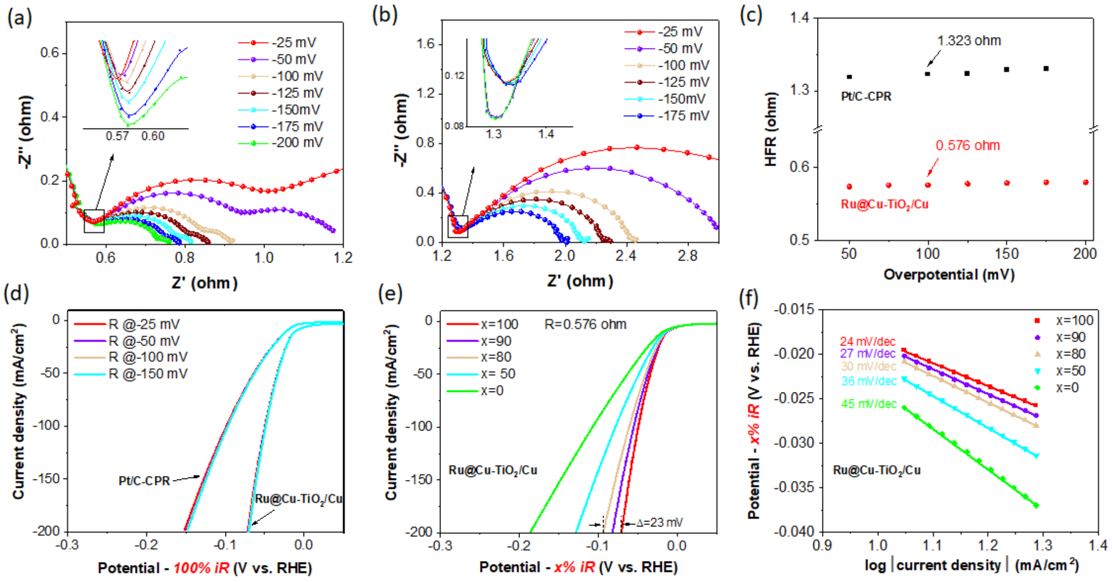

Figure S20. EIS Nyquist plots measured for **a**, Ru@Cu-TiO_2/Cu and **b**, Pt/C-CPR electrode. **c**, The lowest impedance observed in the corresponding Nyquist plot in the high frequency region. The real part of such impendences are referred to uncompensated resistances (R_u). **d**, LSV plots of Ru@Cu-TiO_2/Cu and Pt/C-CPR electrodes after 100% iR compensation. The R_u is obtained from their corresponding Nyquist plot recorded at different working potentials: -25 mV, -50 mV, -100 mV and -150 mV (vs. RHE). **e**, LSV plots of Ru@Cu-TiO_2/Cu electrode after applying different levels of iR compensation. **f**, Tafel plots derived from their corresponding LSV plots shown in (e).

We have conducted EIS measurements on newly prepared Ru@Cu-TiO_2/Cu and Pt/C-CPR electrodes at different working potentials.

As depicted in **Figure S20a,c**, the R_u value for the Ru@Cu-TiO_2/Cu electrode exhibits only a marginal increase (<2 %) as the applied potential is increased from -25 mV_{RHE} (resulting in a R_u of 0.570 ohm) to -200 mV_{RHE} (leading to a R_u of 0.580 ohm). Similarly, the influence of working potential on the Rs value of the Pt/C-CPR electrode is displayed in **Figure S20b**, with a comparably negligible impact observed (**Figure S20c**). Indeed, **Figure S20d** provides evidence that using the R_s determined at different working potentials would result in almost identical LSV plots (after 100% iR-correction), demonstrating the validity of our EIS protocol at -0.1 V (vs. RHE) in the present context.

Regarding the approach used for ohmic resistance (iR) correction, we initially considered a 100% iR-correction in our first submission. However, it is important to acknowledge that the percentage of iR compensation remains an undefined practice within the research community, leading to variations in its adoption. For instance, different studies have employed various levels of iR-correction, such as 100 %,[34–36] 90 %,[37,38] and 80 % [39,40] iR-correction.

Recognizing the challenge of determining an optimal level of iR compensation and the lack of a standardized approach, we have included additional information in our manuscript. Specifically, we have presented data with different levels/percentages of iR compensation for readers to assess the activity of the Ru@Cu-TiO_2/Cu electrode as depicted in this work (see **Figure S20e**). This transparent approach allows readers to consider the potential impact of iR correction on the observed results and draw their own informed conclusions. Nevertheless, we support that 100% iR compensation should be adopted, if no over-compensation is observed, to “clean” the performance decay from the ohmic feature of the system.

As displayed in **Figure S20e**, the LSV plots of the Ru@Cu-TiO_2/Cu electrode exhibit minimal variation in overpotentials required to achieve -200 mA/cm^2 when transitioning from 100% iR compensation to 80%. Remarkably, even with the adjustment in iR compensation, the Ru@Cu-TiO_2/Cu electrode continues to demonstrate a low overpotential of 95 mV, underscoring its notable HER activity. This outcome demonstrates the robustness and effectiveness of our electrode's performance in catalyzing the HER process.

Furthermore, the determination of the Tafel slope is a critical aspect of characterizing electrode performance. Typically, the Tafel slope is derived from the LSV plot, although alternative approaches such as steady-state galvanostatic strategies are also utilized.[41] However, the challenge lies in accurately determining the appropriate level of iR compensation for this analysis, which can impact the calculated Tafel slope.

Given the uncertainty surrounding the optimal degree of iR correction, we have taken a comprehensive approach by showcasing multiple Tafel slopes obtained from LSV plots with varying percentages of iR compensation, as requested by the Reviewer. **Figure S20f** illustrates the variations in Tafel slopes resulting from different iR compensation levels.

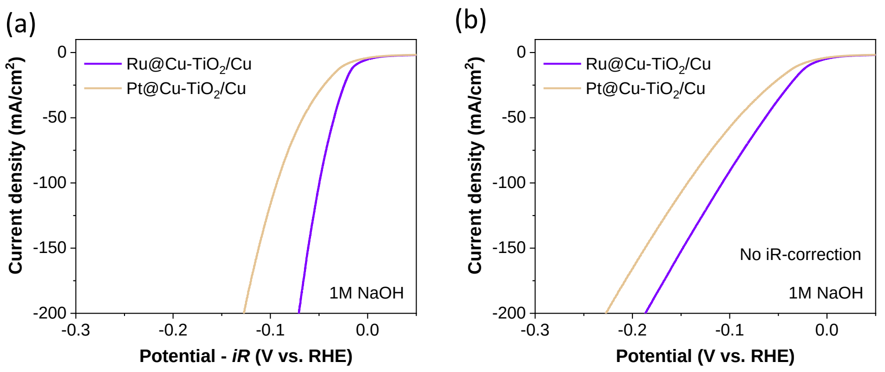

Figure S21. Comparison between **a**, iR-corrected and **b**, non iR-corrected LSV curves measured for Ru@Cu-TiO_2/Cu and Pt@Cu-TiO_2/Cu electrodes.

To fabricate the Pt@Cu-TiO_2/Cu electrode, we followed a same protocol to that of Ru@Cu-TiO_2/Cu synthesis, with the exception that the Ru precursor used in the latter case was replaced by a Pt precursor ($Na_2PtCl_6{\cdot}6H_2O$). Additionally, the Pt precursor solution, containing an equivalent amount of precious group metal (PGM) ions (100 μg_{Pt} for the preparation of 1 cm^2 electrode), was added during the step for electrodeposition of PGM.

We conducted an ICP-OES analysis to determine the mass loading of Pt on the Pt@Cu-TiO_2/Cu electrode. The results indicate that the PGM loading is approximately 54 $\mu g_{Pt}/cm^2$ in the produced Pt@Cu-TiO_2/Cu electrode, closely mirroring the mass loading of Ru in the Ru@Cu-TiO_2/Cu electrode (around 52 $\mu g_{Ru}/cm^2$). Consequently, we can discount the impact of varying PGM loads on performance, and the observed disparities in their performance can be attributed to their intrinsic activity in the context of alkaline HER.

As illustrated in **Figure S21**, it is evident that the Pt@Cu-TiO_2/Cu electrode falls short in comparison with the Ru@Cu-TiO_2/Cu electrode for HER in 1M NaOH. This further underlines the notable role of Ru in the alkaline HER process.

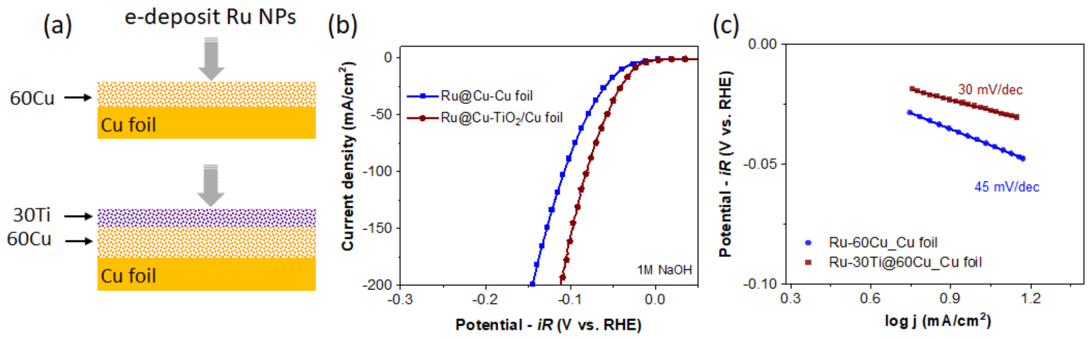

Figure S22. a, Brief scheme of the synthesis protocol of Ru@Cu grown on planar Cu foil with and without TiO_2 present. **b**, LSV plots of Ru@Cu-Cu foil and Ru@Cu-TiO_2/Cu foil electrodes. **c**, The corresponding Tafel plots of electrodes derived from their LSV plots shown in (b).

We have conducted supplementary experiments to investigate the effect of TiO_2 of Ru@Cu-TiO_2/Cu electrode on the HER process. As illustrated in **Figure S22a**, the 3D structured Ru@Cu-TiO_2/Cu and Ru@Cu-Cu (Ru@Cu grown on Cu nanorod, not accessible in our synthesis case) has been produced into planar structured electrodes to simplify our synthesis and investigation here, and in particular, to eliminate any potential morphological modifications arising from the participation of TiO_2, as mentioned above.

The results demonstrate that the TiO_2 layer indeed enhances the alkaline HER performance (**Figure S22b**) of the produced planar electrode, probably by additionally contributing to the water dissociation step (**Figure S22c**). The Tafel slope close to 120 mV/dec indicates that the Volmer step (related to water dissociation) is the rate-determining step, while a Tafel slope close to 39 mV/dec reflects that the Heyrovsky step is the rate-determining step.[42] In our measurements, the electrode with the participation of TiO_2 displays better performance than that without TiO_2 species. Hence, we demonstrate herein that the TiO_2 additionally contributes to the water dissociation step.

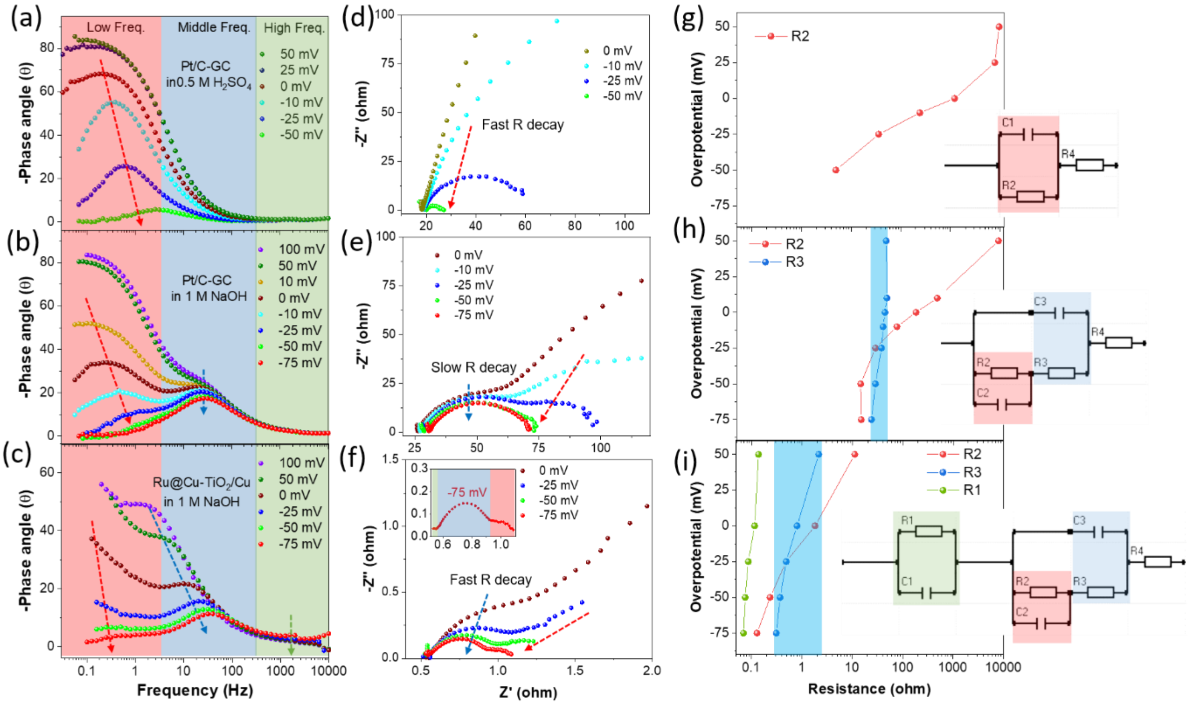

Figure S23. Investigation of HER kinetics of the investigated electrodes using EIS technique. **a-c**, Bode plots and **d-f**, corresponding Nyquist plots measured for Pt/C benchmark on glassy carbon (Pt/C-GC, in 0.5 M H_2SO_4 and 1 M NaOH), and Ru@Cu-TiO_2/Cu (in 1 M NaOH), respectively, at various overpotentials. **g-i**, Resistances (R1, R2 and R3) extracted from the corresponding equivalent electric circuit during HER operation as a function of the potential *vs.* RHE. The x-axes and y-axes have the same scale range to facilitate the resistance comparison. The inset in **g-i**, shows the equivalent electrical circuit used to fit EIS data measured for the electrode/electrolyte systems. The color shadings indicate the different frequency regions: red for low-frequency, blue for middle-frequency, and green for high-frequency.

The HER process in alkaline media proceeds through following steps:

[1] Volmer step ($H_2O + e^- \rightleftarrows H^* + OH^-$, in which H* refer to adsorbed hydrogen),

[2] Heyrovsky step ($H_2O + H^* + e^- \rightleftarrows H_2 + OH^-$), and/or Tafel step ($2H^* \rightleftarrows H_2$).

As can been seen in the above formulas, the Volmer and Heyrovsky steps involve one electron charge transfer, and thus could be detected by EIS technique. In contrast, the Tafel step cannot be displayed by EIS spectra as no electron transfer occurs. According to relevant literature, the Volmer step is associated with the low-frequency region. The middle-frequency region is correlated to the Heyrovsky step. The high-frequency region is related to the electron transfer within the catalyst layer.[43,44]

Figures S23a-c show the Bode plots and their corresponding Nyquist plots **(Figures S23d-f)** recorded on Pt/C benchmark, deposited on glassy carbon electrode of Φ = 3 mm (hereafter named Pt/C-GC), working in 0.5 M H_2SO_4, 1 M NaOH, and Ru@Cu-TiO_2/Cu working in 1 M NaOH. The Bode plots acquired on Pt/C-GC in 0.5 H_2SO_4 (**Figures S23a**) displayed only one phase angle peak in the low-frequency region (representing the Volmer step). The absence of phase angle peak in middle-frequency region (related to Heyrovsky step) implied the step [2] proceeds *via* a Tafel mechanism. Hence, the HER process occurred on Pt/C-GC in acidic media follows a Volmer-Tafel mechanism. Unlikely, the Bode plot of Pt/C-GC tested in 1 M NaOH (**Figure S23b**) show two phase angle peaks in the low-frequency and middle-frequency regions, supporting a Volmer-Heyrovsky mechanism for the HER. In addition, the phase angle peak related to Heyrovsky step in the middle-frequency remained

almost unchanged with increasing the potential, implying relatively sluggish kinetics, and excluding the occurrence of the Tafel process. In contrast, the electrode of Ru@Cu-TiO_2/Cu showed three phase angle peaks in the three frequency regions. The presence of the phase angle peaks in the low and middle-frequency regions indicate that the HER process follows the Volmer-Heyrovsky mechanism. Nevertheless, unlike the Bode profile shown in Pt/C-GC in 1 M NaOH, Ru@Cu-TiO_2/Cu (**Figure S23c**) displayed much faster decay of phase angle peak (and R3) related to Heyrovsky step in the middle-frequency region. This behavior suggests the involvement of Tafel process in step [2]. Therefore, the Ru@Cu-TiO_2/Cu electrode most likely follows a mixture mechanism of Volmer-Heyrovsky and Volmer-Tafel.

Based on the HER mechanisms and the number of phase angle peaks discussed above, suitable equivalent circuit models were used to simulate the Nyquist plots of the investigated electrodes (inset in **Figures S23g-i)**.[44] More specifically, the resistance of the Volmer step (R2), Heyrovsky step (R3) and electron transfer within the catalyst layer (R1), can be calculated as function of the HER overpotential. As shown in **Figures S23h,i**, compared to Pt/C, Ru@Cu-TiO_2/Cu demonstrated fast decreasing resistance for Heyrovsky step (R3) step within the region of tested potentials. This is a sign that the Tafel step occurs after the Volmer step, apart from Heyrovsky step. Hence, the HER on Ru@Cu-TiO_2/Cu surface proceeds a combination of Volmer-Heyrovsky and Volmer-Tafel process.

In addition, as corroborated by our theoretical calculations shown in the main text, surface Ru interacting with surface Cu clusters strongly promote the Volmer step. Interaction of Ru with Cu clusters also exhibits a close-to-zero Gibbs free energy of the hydrogen adsorption, leading to an efficient combined Heyrovsky/Tafel mechanism. Notably, the ultralow resistance for the electron transfer within the catalyst layer (R1, related to the high-frequency region) of Ru@Cu-TiO_2/Cu demonstrates its high electrical conductivity, which is attributed to the vertical alignment of the NRs based on Cu core.

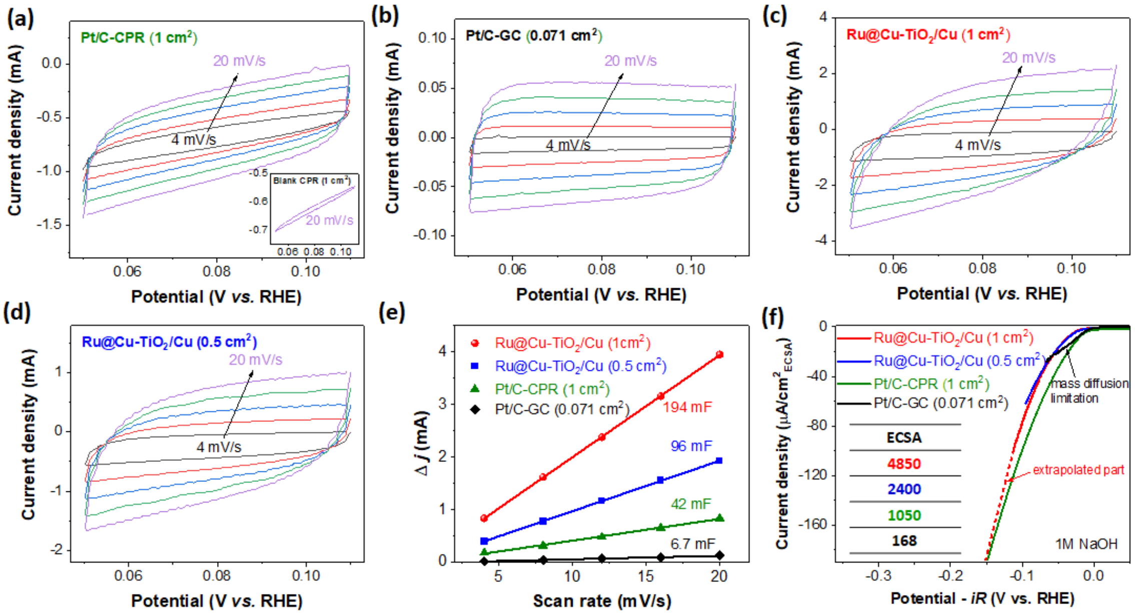

Figure S24. CV curves measured for **a**, Pt/C-CPR (1 cm^2); **b**, Pt/C-GC (0.071 cm^2); (c) Ru@Cu-TiO_2/Cu (1 cm^2); Ru@Cu-TiO_2/Cu (0.5 cm^2) at scan rates of 4, 12, 16, 18 and 20 mV/s. (e) Δ*j* extracted from the corresponding CV curves at 0.08 V (*vs*. RHE) as a function of the scan rates. The absolute double layer capacitance were indicated. (f) Comparison between the iR-corrected LSV curves measured for indicated electrode after ECSA normalization for the current.

Since the structure of Ru@Cu-TiO_2/Cu is complicated, to determine the electrocatalytic surface area (ECSA) of only the active specie (in our case is Cu at the Ru surface) using those strategies based on Cu UPD or H_{upd} become impossible. We therefore quantified the ECSA based on the C_{dl} of electrode catalyst. To do so, we are considering all the components that contribute to C_{dl} as active species (including C within Pt/C, and Cu, Ru, TiO_2 within Ru@Cu-TiO_2/Cu), and the specific activity of measured electrode catalyst is therefore underestimated.

Before assessing the performance and measuring the C_{dl}, both the freshly prepared Pt/C-CPR and Ru@Cu-TiO_2/Cu electrodes were stabilized using multiple CV activation steps. Scan rates below 20 mV/s were used to mitigate the possible impact of ohmic resistance on the measured current. Furthermore, we included testing of a blank CPR sample to discern its potential effects on the measurement of the Pt/C-CPR electrode.

As demonstrated in **Figure S24a**, the CV currents obtained from the Pt/C-CPR electrode remain below 0 when measured at scan rates below 20 mV/s. A similar phenomenon has been documented previously in the literature for self-supported HER electrocatalysts using the carbon substrate.[45,46] In these cases, CV currents below 0 are observed during measurements conducted at low scan rates. We hypothesize that this behavior may arise from a Faradaic reduction processes (e.g., reduction of O-functionalities in CPR) of the porous CPR substrate. Indeed, our testing of the blank CPR yielded a negative current response similar to the shift observed in Pt/C-CPR at the same scan rate of 20 mV/s. Importantly, this contribution from the substrate is not expected to significantly impact the determination of the C_{dl} of the catalyst, as the linear fit of Δj plotted against the scan rate (ν) would merely experience a slight overall shift.

To mitigate the influence of porous carbon on current recording at lower scan rates, we made an additional electrode by depositing the Pt/C dispersion onto the flat surface of glass carbon (Φ = 3 mm).

This resulting electrode is labeled as Pt/C-GC (0.071 cm^2). As depicted in **Figure S24b**, the CV currents exhibit a "normal" behavior, with the zero current point positioned between the cathodic and anodic currents. For the normalization of their LSV plots, ECSA data obtained from Pt/C deposited on both CPR (1 cm^2) and GC (0.071 cm^2) substrates were taken into consideration, as elaborated subsequently.

Shifting our focus to the CV plots obtained from Ru@Cu-TiO_2/Cu electrodes, the CV curves recorded for both the 1 cm^2 case (**Figure S24c**) and the 0.5 cm^2 case (**Figure S24d**) exhibit identical profiles, along with a linear relationship between current and scan rate (**Figure S24e**). Moreover, their absolute double-layer capacitance (C_{dl}) values (in mF, not mF/cm^2) are directly proportional to their respective sizes (194 mF for the 1 cm^2 electrode and 96 mF for the 0.5 cm^2 electrode, as shown in **Figure S24e**). Furthermore, the Pt/C-CPR (1 cm^2) and Pt/C-GC (0.071 cm^2) electrodes also manifest a linear correlation between CV current and scan rate.

The ECSA-normalized LSV plots were shown in **Figure S24f**. We can see that the two Ru@Cu-TiO_2/Cu electrodes with different sizes display almost overlapping LSV plots after ECSA normalization, further demonstrating a good proportionality between absolute current and size. Moreover, although the Ru@Cu-TiO_2/Cu electrode showcases a lower ECSA-normalized activity at lower working potentials (specific current) compared to Pt/C-CPR electrode, it demonstrates the potential for superior performance at higher working potentials, underscoring its heightened HER kinetics under these conditions.

Interestingly, the Pt/C-CPR (1 cm^2) and Pt/C-GC (0.071 cm^2) electrodes exhibit similar LSV profiles at low ECSA-normalized current. However, the latter experiences a notable decline in current at higher working potentials (current), likely due to the mass diffusion limitations. The analogous ECSA-normalized LSV plots for Pt/C-CPR (1 cm^2) and Pt/C-GC (0.071 cm^2), before reaching the mass diffusion limit of the latter, demonstrate that the substrate type doesn't affect the assessment of specific activity of the Pt/C catalyst.

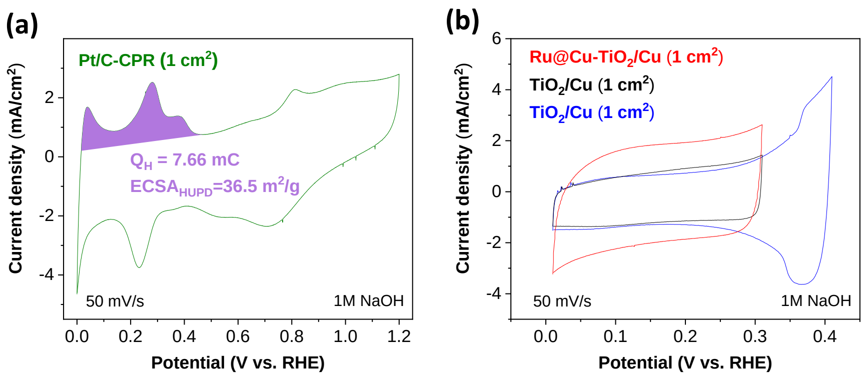

Figure S25. CV curve of **a**, Pt/C-CPR and **b**, Ru@Cu-TiO_2/Cu and TiO_2/Cu electrode. Measurements were carried out in 1 M NaOH (10th CV scan, potential scan rate = 50 mV/s).

Although it seemed impossible to determine only the ECSA of Ru within Ru@Cu-TiO_2/Cu electrode using a Hupd strategy, we still made some attempts. We started with Pt/C-CPR electrode.

For the H_{upd} measurements, we followed the procedure outlined in a previous.[47] The 1M NaOH electrolyte was purged with Ar prior to use, and the 10th CV curve (employed to stabilize the electrode) was subjected to analysis for ECSA calculation. The ECSA was determined by integrating the hydrogen adsorption region (Q_{Hupd}) after implementing a double-layer current correction. A theoretical charge of 210 μC/cm^2, accounting for the adsorption of a monolayer of hydrogen on the surface of polycrystalline Pt (Q_{mono}),[47] was taken into consideration. Thus:

$$ECSA_{Hupd} = Q_{Hupd} / (Q_{mono} \times m_{Pt})$$

We know that the mass loading of Pt (m_{pt}) on CPR (1 cm^2) is 100 μg, then the ECSA of Pt within Pt/C-CPR (1 cm^2) could be calculated as 36.5 m^2/g_{Pt} (**Figure S25a**). This value is very close to reported data on commercial Pt/C.[48]

As attempts, we then conducted H_{upd} measurements on the Ru@Cu-TiO_2/Cu (1 cm^2) electrode. Additionally, we obtained the H_{upd} curve for the TiO_2/Cu electrode to facilitate comparison. As illustrated in **Figure S25b**, integrating the effective area of Ru species from the H_{upd} curve recorded on the Ru@Cu-TiO_2/Cu electrode proves intricate, as no distinct peak is observable. This complexity may be attributed to the reason that hydrogen adsorption on Ru is both weak and sluggish.[49]

Besides, the CV profile of the H_{upd} response acquired on the Ru@Cu-TiO_2/Cu electrode is very similar to that of its TiO_2/Cu counterpart. The broader integration areas observed in the H_{upd} curve could likely originate predominantly from Cu. The enhanced area observed in the Ru@Cu-TiO_2/Cu electrode might be attributed to the greater porosity of its nanorods (predominantly composed of Cu), compared to those of the TiO_2/Cu electrode (as depicted in **Figure 1c** and **Figure 1d**).

Additionally, the oxidation of Cu within the electrode in the H_{upd} region of Ru may also contribute to the current, particularly becoming pronounced as the potential reaches 0.4 V (vs. RHE) (**Figure S25b**).

As a conclusion, it is not possible to reliably quantify the ECSA of Ru within the Ru@Cu-TiO_2/Cu electrode through H_{upd} technique.

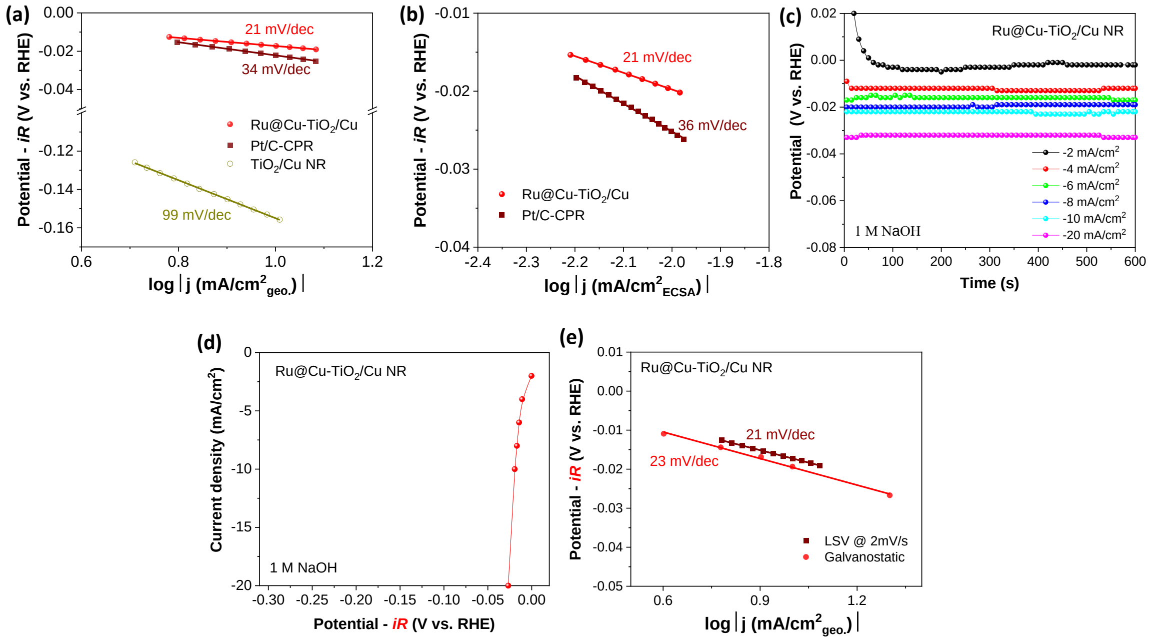

Figure S26. Tafel plots measured for the investigated electrodes: **a**, before and **b**, after ECSA normalization for the current density. **c**, Multiple CP plots measured for the Ru@Cu-TiO_2/Cu at different current densities of -2, -4, -6, -8, -10, and -20 mA/cm^2. **d** Galvanostatic polarization curve acquired from the data extracted from CP curves shown in **a**. **e** Comparison between Tafel slopes determined by Tafel plots acquired through potentiodynamic and galvanostatic protocols. The slope value determined as 21 mV/dec is based on the data extracted from its potentiodynamic LSV plot, while the value of 23 mV/dec was extracted from the galvanostatic polarization curve. The Tafel slope values extracted from the data acquired with the two different methodologies are comparable.

The analysis of the Tafel plots measured on different electrodes for the HER in 1 M NaOH was shown before and after normalizing the current density by its corresponding ECSA (see **Note S1** for more details). In both cases, the Ru@Cu-TiO_2/Cu exhibited a Tafel slope of 21 mV/dec, significantly lower than those of Pt/C-CPR and TiO_2/Cu, regardless of ECSA normalization, suggesting fast HER kinetics on our Ru@Cu-TiO_2/Cu (**Figure S26a,b**).[50] Notably, the Tafel slopes do not significantly change after normalizing the geometric current densities by ECSA. Nevertheless, we still recommend calculating the Tafel slope after ECSA normalization procedure (see **Note S1** for explanation).

To follow the best practices to perform Tafel analysis,[41] the steady-state galvanostatic technique was further carried out to acquire the polarization curves of Ru@Cu-TiO_2/Cu, leading to a Tafel slope of 23 mV/dec (**Figure S26c-e**), similar to that obtained for dynamic measurements. It is worth noting that the slight difference in Tafel slope between the 100 % iR compensation case presented here (24 mV/dec) and the result shown in **Figure S20f** (21 mV/dec) could arise from minor variations between different electrode synthesis batches.

Note S1. Discussion on Tafel slope below 30 mV/dec

In principle, the Tafel slope could be used as the metric to evaluate the HER kinetics. Such parameter can be estimated from the linear portion of the Tafel plot (overpotential *vs.* log(|current density|) curve) according to the Tafel equation:

$$\eta = b \times \log |j| + A,$$

in which the η indicates the overpotential referring to the standard reduction potential of the proton ($E^0_{H+/H2}$ = 0 V *vs.* RHE), *j* is current density, b is the Tafel slope and A is a constant. At low H* surface coverage, the Volmer reaction (*i.e.*, the hydrogen adsorption through water dissociation, $H_2O + e^- \rightleftarrows H^* + OH^-$) is the rate-determining step (RDS) of the HER, and a theoretical Tafel slope of 120 mV/dec is expected. Conversely, with H* surface coverage approaching unity (*i.e.*, when Volmer step is not the RDS), the Tafel slope decreases towards theoretical values of 40 or 30 mV/dec. These values depict specific HER kinetics, with Heyrovsky reaction *(i.e.*, H_2 formation through electron transfer from water to an adsorbed hydrogen intermediate, $H_2O + H^* + e^- \rightleftarrows H_2 + OH^-$) or Tafel reaction (i.e. H_2 formation through coupling of H* intermediates, $2H^* \rightleftarrows H_2$), respectively.[51] According to the theoretical underpinnings of charge transfer and considering the relatively simple and straightforward mechanisms typical of HER, HER Tafel slope should be in the range from 30 to 120 mV/dec.[52] Interestingly, we observed Tafel slopes lower than 30 mV/dec, as also previously reported.[53–57] Acknowledging the absence of physical meaning of Tafel slopes < 30 mV/dec for single electron transfers and being aware of the limitations of either our experimental data and Tafel's approach applicability, we nevertheless provide the following comments to rationalize the obtained results.

1. Potential dependency and ECSA normalization: effects on Tafel slope determination.

Tafel slope has been demonstrated to be potential- and, in turn, coverage-dependent.[58] For instance, bulk Pt disk electrodes in 0.5 M H_2SO_4 exhibit 36–68 mV/dec Tafel slopes at low overpotentials followed by 125 mV/dec slopes for increasing overpotentials.[58] Potential dependency might strongly affect the outcomes of Tafel analyses, as the potential window for the determination of Tafel slope could be varied for different catalysts and/or research groups.

We therefore suggest determining the Tafel slope in different specific current density ranges, as shown in the main text. However, it is important to stress out that an accurate Tafel analysis should consider ECSA-normalized current for the calculation of the Tafel slop.[59] Nevertheless, the accurate determination of ECSA is not straightforward. For example, in our case, the ECSA of Ru@Cu-TiO_2/Cu could be only estimated by the double layer capacitance method, a method non-specific for active sites determination, therefore introducing possible sources of error in our Tafel parameters estimation.[59,60]

2. Tafel approach applicability.

Although a flawed determination of ECSA might introduce errors in the determination of Tafel parameters, the normalization of the current by ECSA does not directly change the Tafel slope in our electrodes. Hence, the normalization strategy cannot explain why the Tafel slope is below 30 mV/dec in our or other reported cases.[53–57] In general, the Tafel analysis should be properly applied on single crystals, and this is why the fundamental study related to Tafel metric is always done on flat single crystals like Pt[61], and sometimes even focusing on specific facet, *e.g.*, Pt(111)[62]. However, practical catalysts, such as our Ru@Cu-TiO_2/Cu, generally contains multiple catalytic species, complicating the HER mechanism. For this reason, we believe that the Tafel metric should be used with caution in these situations.

For instance, if considering the Tafel metric obtained on our Ru@Cu-TiO_2/Cu, the low Tafel slope (21 mV/dec based on potentiodynamic LSV plot, and 23 mV/dec based on galvanostatic protocol shown in **Figure S26**) would suggest a HER mechanism following Volmer-Tafel process. However, if we check the EIS results shown in **Figure S23**, the Ru@Cu-TiO_2/Cu displayed a Heyrovsky step in the middle-frequency region. Therefore, the Ru@Cu-TiO_2/Cu most likely follows the mixture mechanisms of Volmer-Heyrovsky and Volmer-Tafel (see detailed discussion in **Figure S23**). Overall, in accordance with previous literature,[43,63] we suggest combining EIS results to Tafel slope data when studying the catalytic mechanism of the HER for complex catalysts.

3. iR correction

See detailed discussions in **Figure S24**.

4. New mechanism

Theoretically, simple electrochemical redox reactions can be kinetically described by the Butler-Volmer equation:[64]

$$j = j_0 \{\exp(-\alpha f\eta) - \exp[(1-\alpha)f\eta]\}$$

being α the transfer coefficient, f denotes F/RT (F: the Faraday's constant, R: the universal gas constant, T: the absolute temperature), and j_0 is the exchange current density. The transfer coefficient can be determined for an electrode reaction consisting of a single electron transfer step.[65] Currently, the most common approach in kinetic investigations of HER is to ascribe α a value of 0.5.[65] Based on this, the minimal theoretical Tafel slope for the conventional HER process is 30 mV/dec (if Tafel step is rate determine step[52]).

However, the Tafel step might vary if the total number of the electrons involved in the catalytic step or/and the transfer coefficient differs from one. As demonstrated by Antipin et al,[65] Tafel slopes may be as low as 20 mV/dec if three electrons are involved in the steps prior to the RDS, which is also consistent with Fletcher's calculation.[52]

For example, Zhu et al.[66] reported a type of rhodium/silicon nanowire (Rh/SiNW) towards HER in 0.5M H_2SO_4 media with a Tafel slope of 24 mV/dec. A novel mechanism was suggested by them for the catalyst.

Notably, since the HER is a simple process from a catalytic point of view, any suggestion of a new catalytic mechanism should be cautious and justified. Being out of the scope of this work, we do not further delve into this issue.

To conclude, for those readers interested in the theory and applications of microkinetic Tafel analysis, the authors invite to refer to dedicated literature.[52,64,65,67,68]

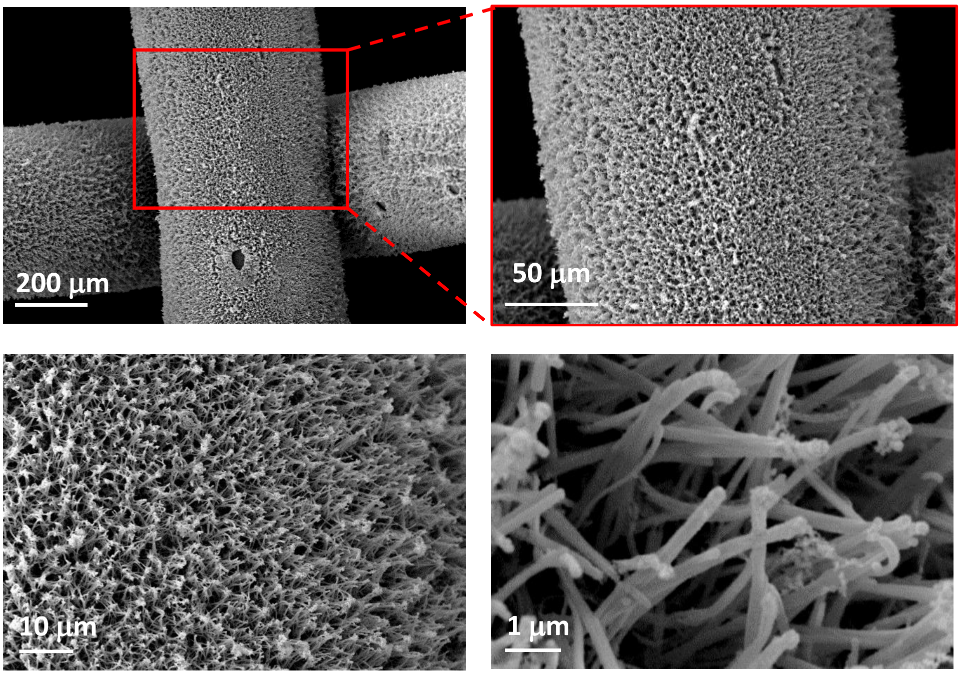

Figure S27. SEM images of the Ru@Cu-TiO_2/Cu acquired at different magnifications after stability test at -200 mA/cm^2 for 250 h in 1 M NaOH.

The excellent stability of our electrode can be ascribed to the intimate contact between Ru nanocrystals and TiO_2/Cu NRs, as well as the adhesion of TiO_2/Cu NRs to Cu substrate, which prevents the agglomeration of Ru nanocrystals and the detachment of the NRs during the HER. This was confirmed by the SEM images of Ru@Cu-TiO_2/Cu measured after the stability test (250 h at -200 mA/cm^2). As shown in **Figure S27**, the NRs were still well anchored to the CM and did not degrade after prolonged HER operation, demonstrating an excellent durability.

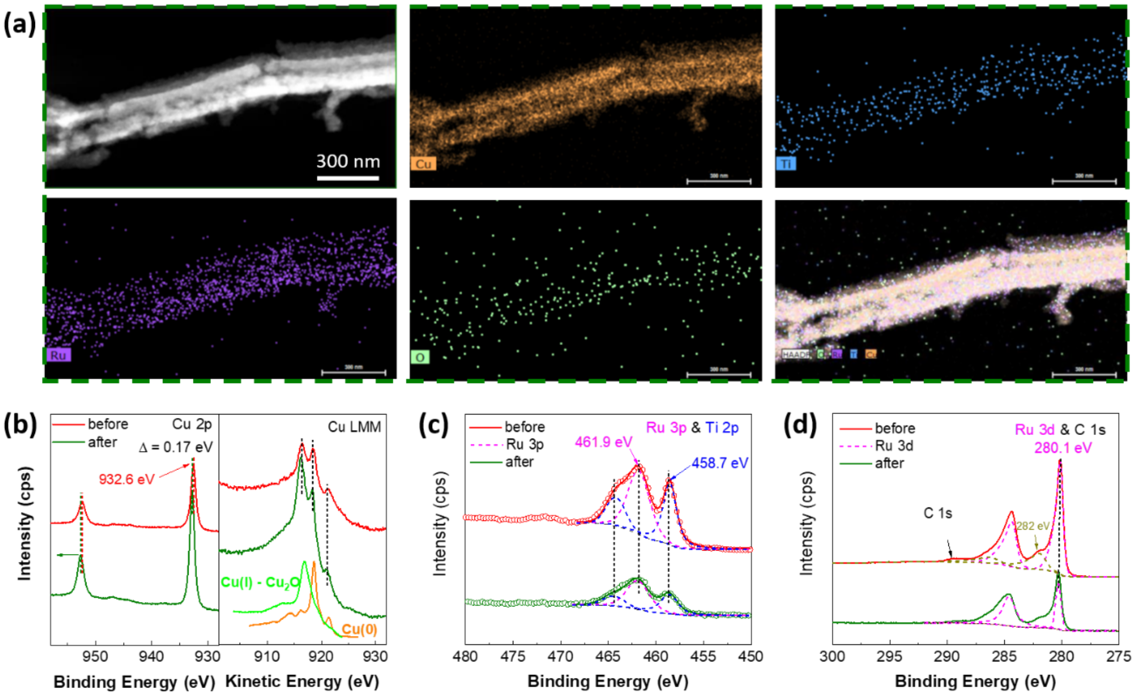

Figure S28. **a**, STEM-EDS characterization of the Ru@Cu-TiO_2/Cu after 160 h stability test at -500 mA/cm^2; XPS/AES analysis of the Ru@Cu-TiO_2/Cu before and after the stability test acquired over the energy ranges typical for **b**, Cu 2p, Cu LMM and references of Cu and Cu(I) of Cu_2O; **c**, Ru 3p (overlapping with Ti 2p); and **d**, Ru 3d (overlapping with C 1s). HER conditions: 1 M NaOH, 27 ^oC.

As shown in **Figure S28a**, the distribution of Ru, Cu, Ti, and O elements was homogeneous throughout the NRS after the stability test of the corresponding Ru@Cu-TiO_2/Cu for 160 h at -500 mA/cm^2. After stability test, the XPS analysis of Ru@Cu-TiO_2/Cu shows an increase of the intensity of the Cu 2p peak, together with a slightly shift towards higher binding energy (**Figures S28b-d)**. The former modification could be ascribed to the increase of Cu amount due to migration of Cu from inner core to the surface through the possible dynamic dissolution-redeposition of Cu in alkaline media under negative potentials.[69,70] Meanwhile, the slight shift in Cu 2p peak could be due to oxidation of some of the Cu to Cu_2O (see in Cu LMM spectra). Notably, although the peak intensity decreased, no obvious modification in the oxidation states and peak locations were observed for Ru and TiO_2 species of the Ru@Cu-TiO_2/Cu after the stability test.

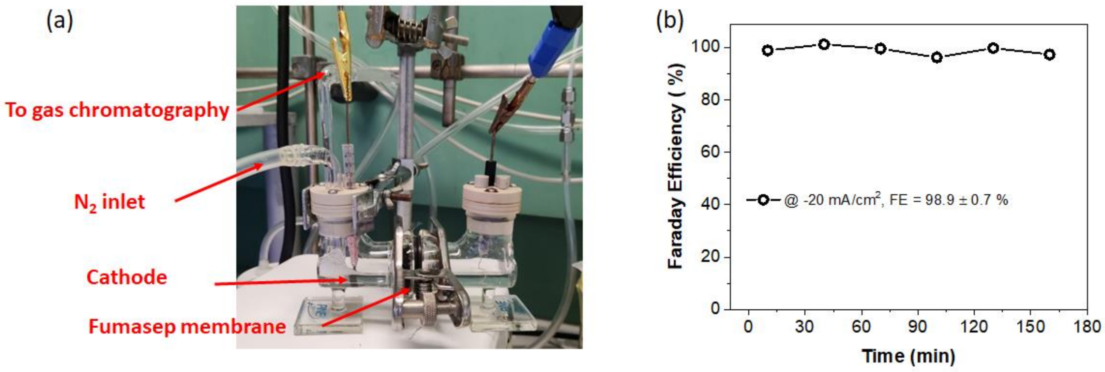

Figure S29. **a**, Photograph showing the H-cell setup used to analyze the Faradaic efficiency of the HER. **b**, Calculated Faradaic efficiency of Ru@Cu-TiO_2/Cu towards the HER *vs.* the time. The average Faradaic efficiency is 98.9 %.

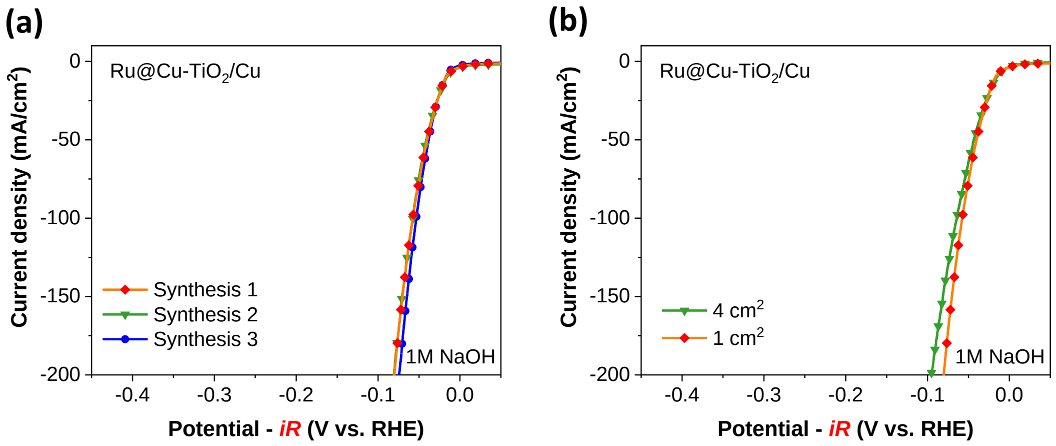

Figure S30. **a**, LSV curves with iR-correction measured for Ru@Cu-TiO_2/Cu from THREE synthesis batches produced using 400 µg Ru precursor in 25 mL bath volume. The data were acquired on electrode with an area of 1 cm^2. **b**, LSV curves with iR-correction measured for Ru@Cu-TiO_2/Cu with different (geometric) areas: 1 cm^2 *vs.* 4 cm^2. The upscaling of the synthesis marginally affects the geometrical performances of the electrode.

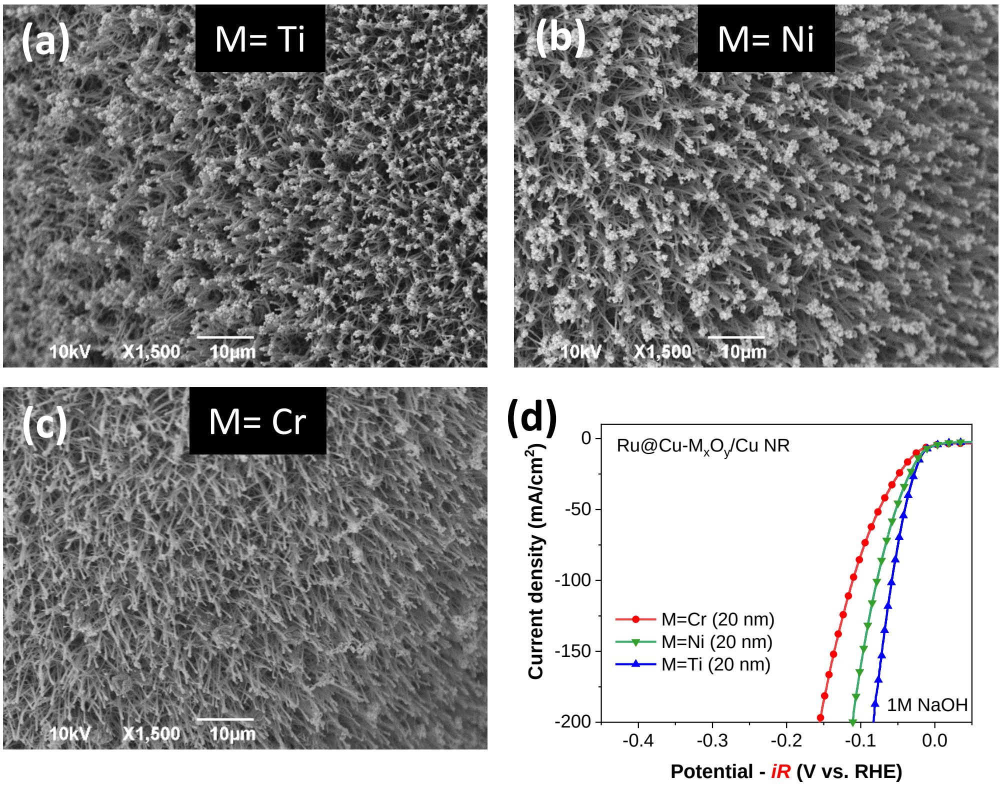

Figure S31. Comparison of **a-c**, morphology and **d**, HER performance of the electrodes prepared by replacing sputtered Ti layer with other metal source (Ni, Cr). The other steps of synthesis procedure were identical to those reported for the Ru@Cu-TiO_2/Cu.

The mass loading of Ru was similar in all cases: Cr case (*ca.* 52.8 µg/cm^2), Ni case (*ca.* 62.5 µg/cm^2), Ti case (*ca.* 55.4 µg/cm^2). All the three electrodes demonstrated excellent catalytici activity towards the alkaline HER. Although the use of Ti layer resulted in the most performant electrode, the use of Cr or Ni layers can be prospectively optimized following the procedures implemented for the Ti layer.

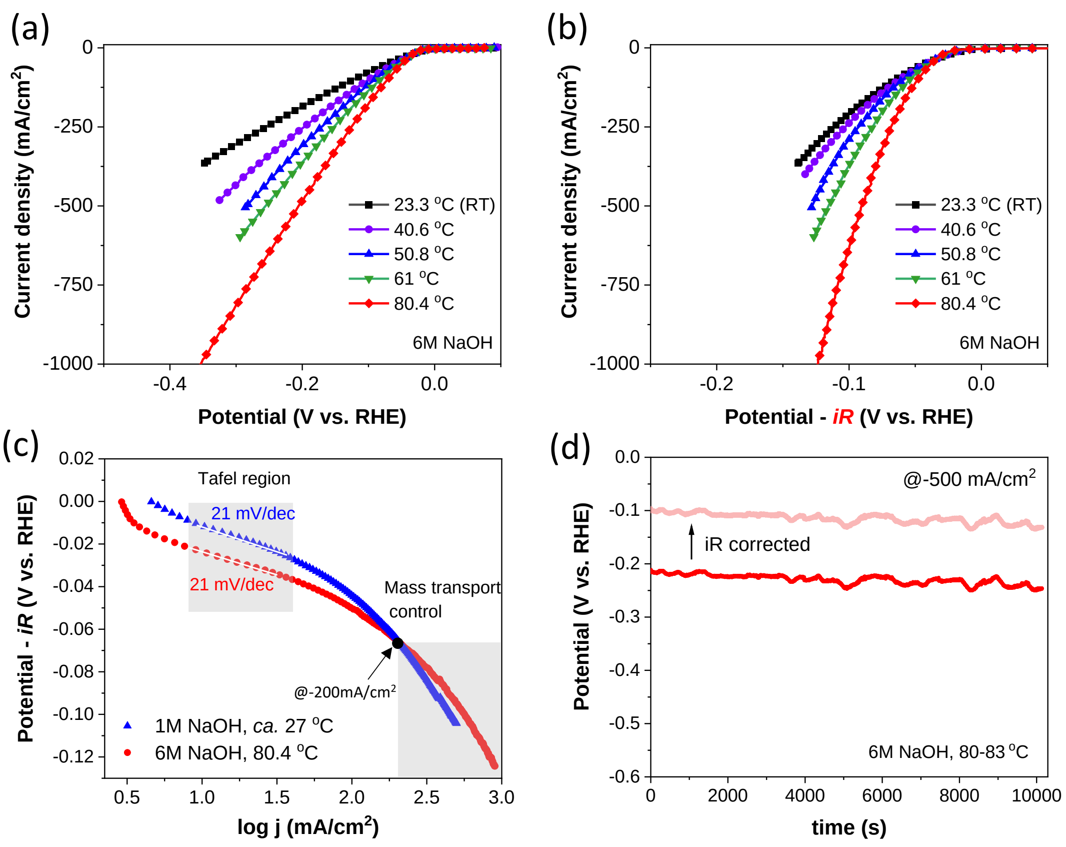

Figure S32. LSV curves measured for Ru@Cu-TiO_2/Cu at simulated AEL conditions (6 M NaOH and temperature up to 80 °C) **a**, before and **b**, after iR-correction. **c**, Comparison between the Tafel curves measured for Ru@Cu-TiO_2/Cu in 1 M NaOH at 27 °C and 6 M NaOH at 80 °C. **d**, CP measurements of Ru@Cu-TiO_2/Cu s operating at -500 mA/cm^2 in 6 M NaOH at 80 °C.

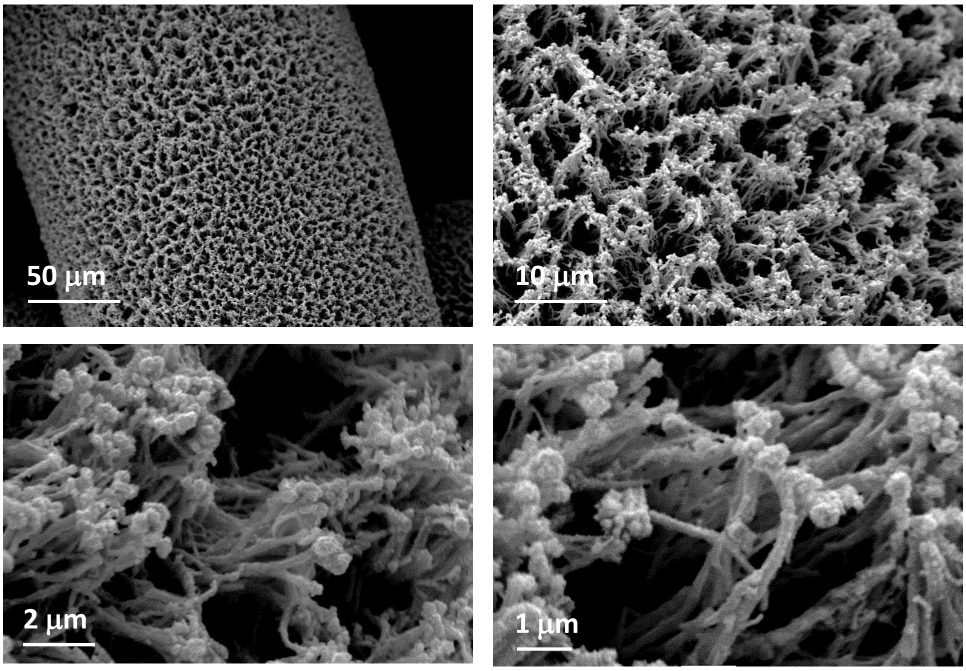

Figure S33. SEM images of the Ru@Cu-TiO_2/Cu acquired at different magnifications after stability test under -500 mA/cm^2 for 30 h. Conditions: 6 M NaOH, 80-83 ^oC.

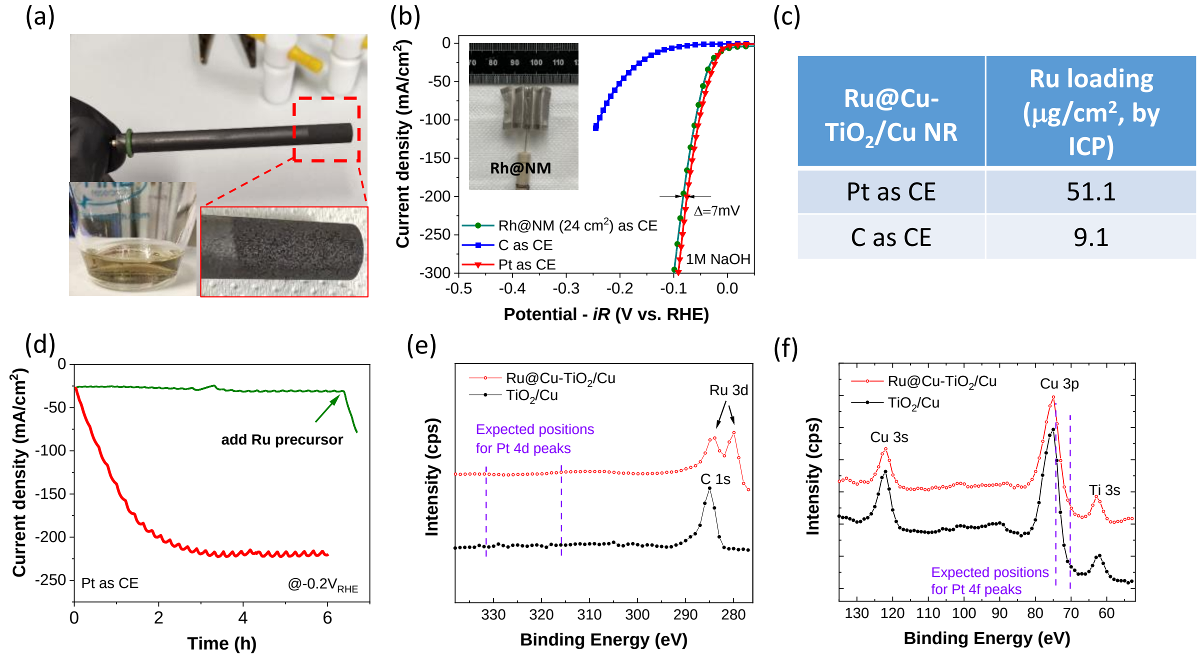

Figure S34. Effect of the counter electrode on electrosynthesis of the Ru@Cu-TiO_2/Cu. **a**, Photographs showing the instability of carbon rod counter electrode during the synthesis of our electrode. **b**, LSV curves of the electrodes synthesized and evaluated using counter electrode of: carbon rod and Pt. **c** Mass loading of the deposited Ru on the electrode produced using carbon rod and Pt as counter electrode. **d**, Comparison between CA plots measured for the electrodes prepared with and without Ru precursor addition into 1 M NaOH at the beginning. Winded Pt wire as counter electrode. **e**, Pt 4d and **f**, Pt 4f XPS spectra acquired on the investigated electrodes. No traces of Pt were found in the investigated Ru@Cu-TiO_2/Cu.

Being aware that a Pt counter electrode (CE) may lead to Pt contamination in our working electrode (WE), we have initially used a dense graphite carbon rod (from Redox.me company) as CE, following best practices.[71] Nevertheless, we observed severe carbon loss at the tip of the carbon rod immersed in the electrolyte (1 M NaOH) during the electrode preparation. The colorless electrolyte turned light brown and carbon ash aggregated at the bottom of the cell **(Figure S34a**). The LSV measurements demonstrated that the electrode produced using carbon rod as the counter electrode performed worse than the one produced using Pt CE (**Figure S34b**). Such differences are clearly attributed to the different Ru loading in the electrodes, being almost 5 times lower on the electrode produced with the carbon CE compared that produced using Pt (9.1 μg/cm^2 *vs.* 51.1 μg/cm^2, by ICP analysis) (**Figure S34c**). These results indicated that the carbon contamination can block the site for Ru deposition. Hence, **the carbon CE was not a suitable option for the synthesis of our electrode.** Actually, because of this, **several works focusing on alkaline HER under high-current density still used Pt as a suitable CE.**[72,73]

Notably, we did not observe any current increase before addition of Ru precursor to initialize the Ru deposition, demonstrating the use of Pt wire as CE doesn't result in the higher HER performance due to its possible deposition onto electrode surface (**Figure S34d)**. Moreover, the **absence of Pt 4d and 4f XPS spectra (Figures S34e,f) on the as-produced Ru@Cu-TiO_2/Cu excluded the presence of Pt** .

Moreover, we recently tried another type of CE: **a nickel foam (24 cm^2) with a deposition of thin Rh layer**. On the one hand, the Rh provides a more robust stability than Pt in terms of corrosion. On the other hand, the significantly larger CE surface (compared to WE) would avoid/minimize the corrosion of CE and could therefore become appropriate for HER study in alkaline media.[74] Using this CE, **the produced electrode indicates a similar performance to the one produced using Pt CE** (considering the small discrepancy on their performance could be within the error of experiments as shown in **Figure S30a**), supporting that the use of winded Pt wire as CE is not the reason for the good performance of synthesized electrode.

In short, we acknowledge that the use of Pt CE is not a perfect choice, and the selection of an appropriate CE for HER research under high operating current conditions remains an unsolved problem.[75]

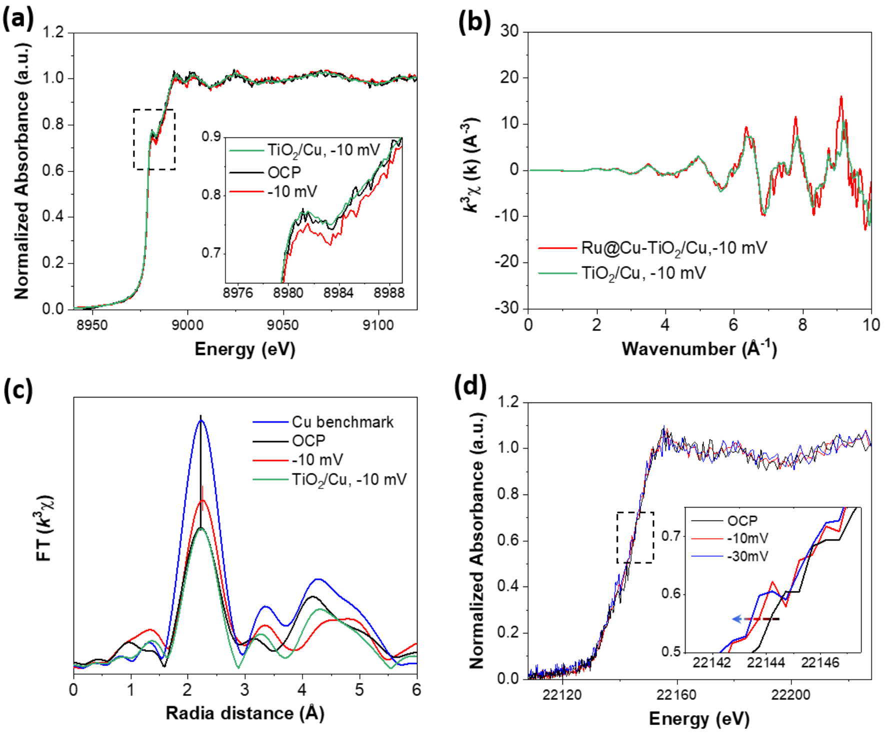

Figure S35. *Operando* XAS measurements. Normalized raw data of **a**, XANES spectra, and **b**, EXAFS $k^3\chi(k)$ signals acquired on Ru@Cu-TiO_2/Cu and TiO_2/Cu at Cu K-edge. **c**, Corresponding Fourier Transform EXAFS spectra real space for Cu K-edge (based on smoothed plot shown in **Figure 4b** in the main text) of tested electrodes. **d**, Raw data of *operando* XANES spectra acquired on the Ru@Cu-TiO_2/Cu at Ru K-edge.

Although eight consecutive scans on Ru K-edge were performed for each step (**Figure S35d)**, the merged XANES spectra were still not perfectly smooth, which could be due to the low content of Ru within the Ru@Cu-TiO_2/Cu electrode, and the perturbation from H_2 bubbles during data collection.

Table S1. Mass loadings of Ti in Ru-yTiO_2/60Cu, as determined by ICP-OES measurements.

Deposited Ti thickness as set (nm)	Real amount (μg/cm^2 by ICP)	Overpotential @-200mA/cm^2 (mV)
10	10.4	150
20	23.5	86
30	31.4	74
40	34.6	95

Table S2. Comparison between the HER activities of various PGM-based catalysts in 1 M KOH/NaOH reported in recent literature.

Sample	Electrode binder	PGM loading	Tafel slope (mV/dec)	η10 (mV) [a]	η50 (mV)	η100 (mV)	η200 (mV)	η500 (mV)	Mass activity (A/mg) [c]	Reference
**$Ru@Cu\text{-}TiO_2/Cu$**	**Self-supporting**	**52 μg/cm^2 (ICP)**	**23**	**16**	**38**	**52**	**74**	**115**	**7.33 @ 100 mV<br>3.39 @ 70 mV<br>0.58 @ 30 mV**	**This work**
$Nb_{0.23}RhO_x/GDY$	Self-supporting	0.3049 wt%	42	14	42	/	/	/	/	*Adv. Sci.* 9, 2104706 (2022)
Ru-Cu-2	Self-supporting	Ru dopants (0.7 wt%)	37	33	~70	/	/	/	3.96 @ 100 mV	*Nano Energy* 92, 106763 (2022)
Ru_1/D-NiFe LDH*	Self-supporting	1.2 wt% of Ru	29	18	/	61	/	/	14.65 @ 100mV	*Nat. Commun.* 12, 4587 (2021)
Pt_{SA}-NiO/Ni @Ag NWs*	Self-supporting	/	27.07	26	~60 [b]	85	/	/	20.6 @ 100 mV	*Nat. Commun.* 12, 3783 (2021)
Pt-Ni NTAs	Self-supporting	16 μg/cm^2	38	23	~45	71	/	/	4.27 @ 50 mV	*Energy Environ. Sci.* 14, 1594-1601 (2021)
Ru/RuO_2	Nafion	/	35	17	~40	~70	/	/	/	*Energy Environ. Sci.* 14, 5433-5443 (2021)
$PtNi_5$-0.3	Nafion	/	19.2	26.8	/	~90	/	/	2.36 @ 70 mV	*Adv. Funct. Mater.* 31, 2008298 (2021)
RuIr@NrC	Nafion	~3 wt% metal	35	28	~70	~100			6.97 @ 100mV	*Chem. Eng. J.* 417, 128105 (2021)
Pt_{SA}-C_1N_1 *	Nafion	~2.5 wt% (ICP)	36.8	46	~120	~200	/	/	/	*Nat. Commun.* 11, 1029 (2020)
Ru@MWCNT	Nafion	/	27	17	/	/	/	/	0.186 @ 20 mV	*Nat. Commun.* 11, 1278 (2020)
Ni_5P_4-Ru	Nafion	/	52	54	/	/	/	/	/	*Adv. Mater.* 32, 1906972 (2020)
Pt_{SA}–$Co(OH)_2$@Ag NW *	Self-supporting	~2.8 wt% determined by ICP	35.72	29	77	104	/	/	1.6 @ 100mV	*Energy Environ. Sci.* 13, 3082-3092 (2020)
NiRu-MOF/NF	Self-supporting	Ru/Ni = 5.4/94.6	90	51	~110	156	/	/	/	*ACS Appl. Mater. Interfaces* 12, 34728 (2020)
Pt/Ni_3S_2/NF	Self-supporting	53 μg/cm^2	73	10	/	~90	~120	207	5.52 @ 150 mV	*ACS Appl. Mater. Interfaces* 12, 39163 (2020)

Pt_{at}–CoP MNSs/CFC *	Self-supporting	5.89 μg/cm^2	30.28	~13	~30	/	/	/	3 @ 70 mV	*J. Mater. Chem. A* 8, 11246 - 11254 (2020)
Cu-Ru/Ti	Self-supporting	63 μg/cm^2	34	23	65	99	140	/	~1.59 @ 99 mV [d]	*J. Mater. Chem. A* 8, 10787-10795 (2020)
pAu_3Pt/NF	Self-supporting	/	42.3	/	40.1	~55	/	/	/	*Sustainable Energy Fuels* 4, 4878-4883 (2020)
Ru NCs/BNG	Nafion	17.17 wt% Ru	28.9	14	50	/	/	/	/	*Nano Energy* 68, 104301 (2020)
Sr_2RuO_4	Nafion	/	61	51	~130	/	/	/	0.4 @ 100 mV	*Nat. Commun.* 10, 149 (2019)
RuNi-NSs@PANI	Self-supporting	7.2wt%	38.54	21.9	/	~160	/	/	/	*J. Catal.* 375, 249-256 (2019)
Ru-MoS_2/CC	Self-supporting	Ru in Ru-MoS_2: 0.37 wt%	114	41	~110	171	/	/	/	*Appl. Catal. B: Environ.* 249, 91-97 (2019)
Au-Ru NWs	Nafion	/	30.8	50	/	/	/	/	/	*Nat. Chem.* 10, 456–461 (2018)
Co-substituted Ru	Nafion	153 μg/cm^2	29	13	~30	/	/	/	~0.196 @ 30 mV [d]	*Nat. Commun.* 9, 4958 (2018)
RuP (L-RP/C)	Nafion	21.4 wt%	34	18	~55	71	/	/	/	*Adv. Mater.* 30, 1800047 (2018)
Pt-Ni ASs	Nafion	17 μg/cm^2	27	27.7	~70	/	/	/	2.8 @ 70 mV	*Adv. Mater.* 30, 1801741 (2018)
PtCo–Co/TiM	Self-supporting	/	35	28	~60	~100	/	/	/	*Nanoscale* 10, 12302-12307 (2018)
Ru@C_2N	Nafion	~285 μg/cm^2	38	17	/	/	/	/	~0.035 @ 17 mV [d]	*Nat. Nanotech.* 12, 441–446 (2017)
Hexagonal Pt-Ni alloy	Nafion	11.5 at%	74	65	/	/	/	/	/	*Nat. Commun.* 8, 15131 (2017)
RuCo@N-C	Nafion	3.58 wt.%	31	28	~130	218	/	/	/	*Nat. Commun.* 8, 14969 (2017)
Pt_3Ni_2-NWs-S/C	Nafion	15 μg/cm^2	/	42	/	/	/	/	~0.667 @ 42 mV [d]	*Nat. Commun.* 8, 14580 (2017)
Pt–$Co(OH)_2$/CC	Self-supporting	5.7 wt %	70	32	~75	122	/	/	/	*ACS Catal.* 7, 7131 (2017)
RuP_2@NPC	Nafion	/	69	52	~110	/	/	/	/	*Angew. Chem. Int. Ed.* 56, 11559–11564 (2017)

Note: [a] Due to the possible current contribution from the substrate for *in-situ* fabricated electrodes, the electrode performances were evaluated at current densities higher than 50 mA/cm^2 when reliable evaluation practices are not indicated. [b] The overpotential values with the symbol (~) are extracted from their corresponding LSV plots. [c] Mass activity is obtained by dividing the current density by the corresponding loading amount of PGMs at a certain overpotential. [d] Calculated value.

Acronyms: NWs – nanowires; SA, at and * - single atom; NF - nickel foam; CNT - carbon nanotube; GDY – graphdiyne; NrC - N-rich carbon matrix; CFC - carbon fiber cloth.

Table S3. Comparison between the TOF of various PGM-based catalysts in 1 M KOH/NaOH reported in recent literature.

Sample	TOF (S^{-1}) @ η=100 mV	Reference
**Ru@Cu-TiO_2/Cu**	**3.85**	**This work**
Ru-Cu-2	0.309	*Nano Energy* ***92****, 106763 (2022)*
Ru_1/D-NiFe LDH	1.27	*Nat. Commun.* ***12****, 4587 (2021)*
Pt/Ni_3S_2/NF	1.41	*ACS Appl. Mater. Interfaces* ***12****, 39163–39169 (2020)*
RuNi-NSs@PANI	0.0498	*J. Catal.* 375, 249-256 (2019)
Te@Ru-0.6/C	0.82	*Chem. Commun.* ***55****, 1490–1493 (2019)*
Sr_2RuO_4	0.90	*Nat. Commun.* ***10****, 149 (2019)*
Ni@Ni_2P-Ru	1.1	*J. Am. Chem. Soc.* ***140****, 2731–2734 (2018)*
Ru@NG	0.776	*J. Mater. Chem. A* ***6****, 13859–13866 (2018)*
PtCo–Co/TiM	1.23	*Nanoscale* ***10****, 12302–12307 (2018)*

First-principles simulations

Note S2: Methods for density functional theory simulations

Forces and energy calculations. Density Function Theory (DFT) simulations were performed through the Vienna Ab initio Simulation Package (VASP).[8] The Perdew-Burke-Ernzerhof (PBE)[9] exchange correlation functional was adopted within the framework of the projector augmented-wave (PAW) method.[10] A plane-wave energy cutoff of 500 eV was adopted throughout. Both self-consistent field procedures (for Kohn-Sham equations) and geometry optimizations were stopped when the energy difference between two successive cycles was lower than $1x10^{-6}$. The reciprocal space was sampled with the following k-points grids: 17x17x17 for bulk Cu, 15x15x9 for bulk Ru, and 3x3x1 for all slabs except the larger one (“Ru_xCu_{19}”) for which a 2x2x1 grid was adopted. This sampling scheme produces a roughly equal k-points spacing for all systems investigated, so as to avoid accuracy biases. Molecules in vacuum were instead simulated with a single k point (Gamma-point only).

Evaluation of reaction energies. The energies of reactions (2)-(4) of the main text were evaluated, respectively, through eqs. E2.1-E2.3:

$$\Delta E_2 = E(\text{slab-}H_2O) - E(H_2O) \qquad [E2.1],$$

$$\Delta E_3 = E(\text{slab-H}) + E(\text{slab-OH}) - E(\text{slab-}H_2O) - E(\text{slab}) \qquad [E2.2],$$

$$\Delta E_4 = E(\text{slab-OH}) + ½\, E(H_2) - E(\text{slab}) - E(H_2O) \qquad [E2.3],$$

in which E(slab) is the energy of a metal slab (Cu, Ru, or Cu-Ru surface alloy), while E(slab-”fragm”, “fragm”=H, OH or H_2O) is the energy of a metal slab with the “fragm” molecule or fragment adsorbed onto it. Only the most energetically favorable types of adsorption sites were considered, namely hollow-fcc sites for H and OH and atop for water. E_{H2O} and E_{H2} are the energies of isolated H_2O and H_2, respectively. Note that, as discussed in the “Experimental Section” of the main text, the reaction energy ΔE_4 corresponds to the reaction (6) of the main text, instead of eq. (4). However, in **Note S3.1** we demonstrate that, at the electric potential used for HER, the (free) energy change of those two reactions must be the same. Note also that, for reactions (2)-(4) of the main text, instead of the Gibbs free energy, whose changes along reactions determine chemical equilibria, we considered only the electronic energy (*i.e.,* the internal energy without vibrational contributions). The latter is the dominant term in the free energy, although other small yet non-negligible contribution exists, mainly the change in solvation and vibrational free energies upon adsorption. However, these two factors are independent from the catalyst adopted, hence they are not relevant for our (mostly qualitative) comparison of reaction energies among Cu-Ru alloys. For reactions (1) and (5) of the main text, instead, we estimated the Gibbs free energy changes, as described below.

Determination of the free energy of hydrogen adsorption and Volmer step (eqs. 5 and 1 of the main text, respectively). We obtained the free energy associated to the hydrogen desorption reaction by adding the contribution of entropy and zero-point vibrational energy to the electronic energy obtained from DFT, as done by Nørskov et al.[76]. However, we also considered the entropy of the adsorbed hydrogen, neglected in ref. 76. We adopted the value determined experimentally in ref. 77 for low-coverage H on Cu, that is 60 J mol^{-1} K^{-1}, corresponding to an entropic contribution ($T\Delta S$) to the adsorption free energy at T = 300 K of 0.11 eV (normalized to ½ H_2) rather than 0.21 eV of ref. 76. Thus, the expression for the free energy, accounting for entropy and zero-point vibrational energy changes, becomes:

$$\Delta G^{desorb}(½H_2) = \Delta E^{desorb}(½H_2) - 0.14 \text{ eV} \qquad [E2.4],$$

Where $\Delta E^{desorb}(½H_2)$ is the energy of H_2 desorption (eq. 5 of the main text) as determined by static DFT simulations, while $\Delta G^{desorb}(½H_2)$ is the corresponding free energy. This equation was applied to produce the plots of **Figure 5c** of the main text, since in the second part of the reaction, $H^*+OH^- \rightarrow ½ H_2+OH^-$, the OH^- ion does not participate in the reaction. Moreover, since the reactants and products have to have the same Gibbs free energy at the electrochemical potential adopted for the HER, the free energy for the step $H_2O+e^- \rightarrow H^* + OH^-$ is to be the same (opposite in magnitude) as the second part of the reaction, as also demonstrated in Note S3. Thus, the plot in **Figure 5c** is a Gibbs free energy diagram.

Systems settings. Full optimizations (cell and atomic positions) of bulk Cu and Ru were performed. The resulting geometries were adopted to build 4-layer slabs to be used for the calculations of adsorption/desorption energies. In these simulations, the bottom layer atoms and cell parameters were kept frozen at the bulk geometry (to simulate the effect of the underlying bulk material), while all remaining atoms were fully relaxed (geometry-optimized). The slabs were separated by a 10 Å vacuum along the z direction. Test calculations on Cu showed that the (electrostatic) interaction among slab images along z is negligible: by increasing the vacuum thickness to 17 Å, the adsorption energies changes by less than 2 meV/molecule (1.2, 0.2, and 0.3 meV for H_2O, H_2, and OH adsorption, respectively). On pure Cu and Ru, all possible adsorption positions were tested, and for both metals the most stable position was hollow-fcc (adsorbant lying among 3 metal atoms without a metal atom in the layer below) for OH and H, while for H_2O the most stable position was on top of a metal atom, with the lone pair pointing towards the surface (as deduced from the H-O-metal angle of 106°). For H_2O, a rotation of H atoms around the O-metal axis did not change the adsorption energy significantly (< 10 meV), even for Cu atoms surrounded by both Cu and Ru. The adsorption positions/sites just described were then adopted for all adsorption simulations on Cu-Ru alloys, except for the CuRu site in the "Ru_xCu_3" slab, for which no hollow-fcc site existed with that stoichiometry, so the hollow-hcp site was adopted. The error in doing so was estimated from the results on pure Cu to be 3 meV and 60 meV for H and OH adsorption, respectively (hence small enough not to change the overall energetic scenario). Finally, the isolated molecules for the calculations of adsorption energies were simulated by surrounding them with a 10 Å vacuum in all three Cartesian directions and optimizing their atomic positions with the DFT settings described in the previous paragraph.

Note S3: Thermodynamic considerations

S3.1: Equivalencies concerning the Volmer step and the OH adsorption (free) energies.

Equivalency between Volmer step and H_2 adsorption (free) energies.

Here we demonstrate that, at the electric potential adopted for the hydrogen evolution reaction (HER), the free energy for the Volmer step is equivalent to the hydrogen adsorption energy. At that electron potential, the following reaction can be considered at equilibrium:

$$2H_2O + 2e^- \rightleftharpoons H_2 + 2OH^- \qquad \text{(R 3.1)},$$

hence the corresponding Gibbs free energy change, ΔG, is zero (considering the ideal reaction, *i.e.* neglecting realistic effects such as voltage drops and the corresponding overpotential applied). Therefore, we have:

$$G(H_2O) + G(e^-) = ½\, G(H_2) + G(OH^-) \qquad \text{[E 3.1]},$$

G being the free energy of the various species. If we plug this equivalency into the equation for the free energy of Volmer reaction step (reaction 1 of the main text), we obtain:

$\Delta G^{Volmer} = G(slab\text{-}H) + G(OH^-) – G(slab) - [G(e^-) + G(H_2O)] =$

$= G(slab\text{-}H) + G(OH^-) – G(slab) - [G(OH^-) + ½ G(H_2)] =$

$= G(slab\text{-}H) - G(slab) – ½ G(H_2) = \Delta G^{ads}{}_H$ [E 3.2].

The meaning of the labels in round brackets is the same as in the eqs. E2.1-E2.3 of note S2. Thus, at the (ideal) electrode potential of HER, the free energy of the Volmer step is equivalent to the free energy of hydrogen adsorption. This is also understandable from a simple qualitative chemical consideration. As HER has ΔG=0 at the electric potential considered here (see above), the energy change associated to the Volmer step must be the same magnitude (opposite sign) as that associated to the completion of HER. Once the Volmer step has taken place, hydrogen desorption is the last step needed to complete the HER. It follows that hydrogen adsorption (*i.e.* the opposite of hydrogen desorption) and Volmer reaction must have the same ΔG.

Equivalency between electrochemical OH^- desorption and OH^- desorption involving H_2O and H_2.

We now demonstrate that, at the ideal potential of HER (see above) the energy for the OH desorption reaction (reaction 4 of the main text), namely

$OH^* + e^- \rightarrow OH^- + *$ (R 3.2),

is fully equivalent to the energy of the following reaction:

$OH^* + ½ H_2 \rightarrow * + H_2O$. (R 3.3).

The free energy change of reaction (R 3.2) is:

$\Delta G^{desorb}(OH^-) = G(OH^-) + G(slab) - G(slab\text{-}OH) - G(e^-)$ [E 3.3],

Calculating this $\Delta G^{desorb}(OH^-)$ directly is a daunting task, since it involves, besides the precise estimation of the electron free energy, the calculation of solvation energy change upon hydroxyl adsorption from solution. These quantities are generally one order of magnitude greater than the reaction energy itself (*e.g.* the hydration energy of OH^- is 4.6 eV[78]), hence even relatively small errors will make the reaction energy completely inaccurate. However, if we apply the equivalency [E 3.1] (moving $G(H_2O)$ to the right side) to [E 3.3], we obtain:

$\Delta G^{desorb}(OH^-) = G(OH^-)+G(slab)-G(slab\text{-}OH)-[½ G(H_2) + G(OH^-) - G(H_2O)]$

$= G(slab)-G(slab\text{-}OH)- ½ G(H_2) + G(H_2O)$ [E 3.4],

That is precisely the free energy change of reaction (R 3.3), *quod erat demonstrandum*.

S3.2: Energetics of Cu and Ru doping.

We studied the energetics of Cu- and Ru- substitution by considering the surface as a grand-canonical ensemble. Within this approach, that is commonly adopted to investigate the surface energy of non-stoichiometric surfaces in alloys and semiconductor compounds,[79] the bulk metal is considered as a reservoir, implying that the initial (final) chemical potential of atoms that migrate to (from) the surface is the same as in the bulk compound. Here, since Cu and Ru are thermodynamically immiscible, the bulk chemical potential considered is that of the pure element, *i.e.,* the energy per atom of the pure compound. Specifically, the free energy change for substituting N atoms of a metal (Me1) on the surface of another metal (Me2) is given by:

$\Delta G^{subst} = G(slab\text{-}Me1_xMe2_N) + N\, G(bulk\text{-}Me1) - G(slab\text{-}Me1_x) - N\, G(bulk\text{-}Me2)$.

In this expression, G(slab-Me1x) is the free energy of the slab made of metal 1, G(slab-$Me1_xMe2_N$) is the free energy of the slab made of metal 1 in which N atoms were substituted by metal 2, and G(bulk-Me1) is the free energy per atom of metal 1 in the bulk (same goes for G(bulk-Me2)).

For example, to calculate the energy of formation of the slab Ru_xCu_3 (**Figure 5** of the main text) from pure Ru, *i.e.* the energy of substitution of 3 Cu atoms on a Ru surface, the following equation is adopted:

$$\Delta G^{subst} = G(slab\text{-}Ru_xCu_3) + 3\ G(bulk\text{-}Ru) - G(slab\text{-}Ru) - 3\ G(bulk\text{-}Cu).$$

We investigated the substitution energies for the Cu-Ru slabs shown in **Figure 5** of the main text as well as of those shown in **Figure S36**. We approximated the free energy with the static (electronic) energy calculated through DFT, thus neglecting vibrational, solvation, and entropic contributions. Indeed, for these types of simulations the vibrational effects can be expected to be fully negligible (the difference in vibrational levels between the bulk and the surface is comparable between Cu and Ru), and solvation effects hardly play any role here (the solvation energy of surface atoms can be expected to be similar between Cu and Ru). As to the entropic contribution, a qualitative estimation can be obtained through the solid solution model ($S=k_B \ln[N_{tot}!/N_A!N_B!]$, S being the entropy and N the number of lattice sites). This simple calculation shows that the -TΔS contribution at T=300 K amounts to -7 meV/atom ($=k_B*\ln[30!/27!3!]*300/30$) for the mixing of 3 atoms per cell on the surface, that is for the Cu_xRu_3/Ru_xCu_3 systems. Thus, the entropic contribution is negligible, too, and the approximation of free energy by electronic energy remains valid. The results are shown in **Table S4**. It can be clearly seen that, as mentioned in the main text, the substitution of Cu atoms by Ru is highly unfavorable. Therefore, although in principle the corresponding surface may form as metastable state, they will not constitute a significant part of the sample. A similar reasoning can be applied to the substitution of subsurface atoms of Ru by Cu. Cu substitution on the Ru surface, instead, is strongly exothermic and thus it is likely that a significant part of the Ru surface in the catalyst if covered by one or more layers of Cu.

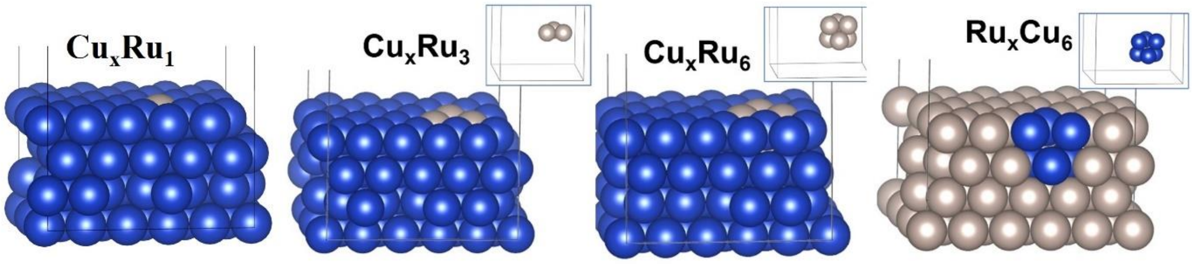

Figure S36. Slabs considered for the calculation of substitution energy (in addition to those shown in **Figure 5a** of the main text). Each slab is labelled to be identifiable in **Table S4**. For the slabs with more than one doping atom, the top right inset shows the slab with only the substituting atoms, to show clearly which atoms were substituted.

Table S4. Substitution energies on various Cu-Ru slab as calculated by DFT simulations.

Slab label	host/main atom type	substituting atoms per cell	ΔE^{subst} (eV/atom)
Ru_xCu_3	Ru	3 Cu on the surface	-0.07
Ru_xCu_6	Ru	6 Cu, 3 on surface, 3 in subsurface	+0.20
Cu-overlayer	Ru	30 (whole surface layer)	-0.29
Cu-stripe	Ru	15 (half of the whole surface layer)	-0.22

Ru_xCu_{19}	Ru	19 Cu on the surface	-0.21
Cu_xRu_1	Cu	1 Ru on the surface	+1.26
Cu_xRu3	Cu	3 Ru on the surface	+1.10
Cu_xRu3	Cu	6 Ru, 3 on surface, 3 in subsurface	+0.75

Note S4: Additional simulation results

4.1: Atomic charges

The atomic charges calculated according to the Quantum Theory of Atoms in Molecules (QTAIM),[80] also known as "Bader charges", are shown in **Table S5** for three representative compounds. For one of them, the atomic charges obtained through the Hirshfeld-I method,[81] are also reported to show that the results are qualitatively equivalent (Hirshfeld-I shows a somewhat greater charge transfer). Note that, in metals, due to the so-called "overspill" effects, surface atoms tend to be more negatively charged with respect to the underlying atoms, especially for Bader charges, as the atomic basins of surface atoms extend towards the vacuum. Thus, a meaningful comparison should be made among atoms on the surface and not between surface and bulk atoms. To avoid lengthy tables, we report the charges averaged for each layer in the slab.

Table S5. Atomic charges in three representative systems (Ru_xCu_3, Cu-overlayer, Cu-stripe, see **Figure 5** of the main text). The average is reported for charges of atoms of the same type and belonging to the same slab layer. For Cu-stripe, the Hirshfeld-I charges are also reported, in square parentheses.

layer # (from top (1) to bottom (4))	species	average charge (*e*)
	Ru_xCu_3	
1	Cu (3 atoms)	+0.02
1	Ru (17 atoms)	-0.06
2	Ru (30 atoms)	+0.05
3	Ru (30 atoms)	+0.05
4	Ru (30 atoms)	-0.05
	Cu overlayer	
1	Cu (30 atoms)	+0.01
2	Ru (30 atoms)	-0.03
3	Ru (30 atoms)	+0.09
4	Ru (30 atoms)	-0.08
	Cu stripe	
1	Cu (15 atoms)	+0.01 [+0.11]
1	Ru (15 atoms)	-0.06 [-0.07]
2	Ru (30 atoms)	+0.02 [-0.04]
3	Ru (30 atoms)	+0.07 [+0.03]
4	Ru (30 atoms)	-0.06 [+0.01]

4.2: Ru@Cu strain calculation.

The strain value (ε) can be calculated through the following equation:

$\varepsilon\%=(d_{Ru\text{-}Ru}-d_{Cu\text{-}Cu})/d_{Ru\text{-}Ru}*100=5.58\%$

where d_{Cu-Cu} and d_{Ru-Ru} are the Cu-Cu and Ru-Ru bond lengths on the respective surfaces. The relatively small value obtained (5.58%) indicates that Cu(111) can grow on the Ru(001) surface, as indeed observed experimentally.[82]

4.3: Geometric *vs.* electronic effects: results on model systems.

We performed simulations to understand whether the changes in adsorption energies on Cu sites in Cu-Ru surface layers with respect to pure Cu are due to geometric/strain (*i.e.,* changes in the geometry of the binding site) or electronic (*i.e.,* changes in the electronic structure due to Cu-Ru interactions) effects. In particular, we focused on the Cu-overlayer system. We built two model systems:

1. The Cu-overlayer slab compressed by 5.58%, so that the geometry of the first two layers of the slab is identical to that of Cu (the first two layers in Cu(111) of fcc Cu and Ru(001) are topologically identical). This system mostly excludes strain effects from the overlayer, since its geometry is the same as in Cu.
2. A slab with the Cu-overlayer geometry made of Cu atoms only, *i.e.,* all Ru atoms are substituted by Cu. This system maintains the strain as in the Cu-overlayer (*i.e.,* geometric effects are mostly unchanged with respect to Cu-overlayer), but excludes the electronic effects due to the Cu-Ru interaction.

The results on the adsorption/desorption energies are reported in **Table S6**. For H, the strain plays the major role in determining the adsorption/desorption energy, while for OH the electronic effects are dominant. Yet, in both cases, neither electronic nor geometric effects are fully negligible. For H_2O, geometric and electronic effects contribute equally. Thus, we conclude that the changes in adsorption energies on Cu-doped Ru with respect to Cu are due to an interplay between strain/geometric and electronic effects.

Table S6. Adsorption energies on Cu, Ru, Cu-overlayer model (see main text), and the model systems described in **Note S4.3**.

system	ΔE^{desorb}_{H2}	ΔE^{desorb}_{OH-}	ΔE^{ads}_{H2O}
Cu	0.22	-0.03	-0.20
Ru	0.44	0.28	-0.44
Cu-overlayer	0.25	0.32	-0.28
model 1 (no strain)	0.32	0.03	-0.24
model 2 (no Ru)	0.25	0.25	-0.24

Evaluation of AEL

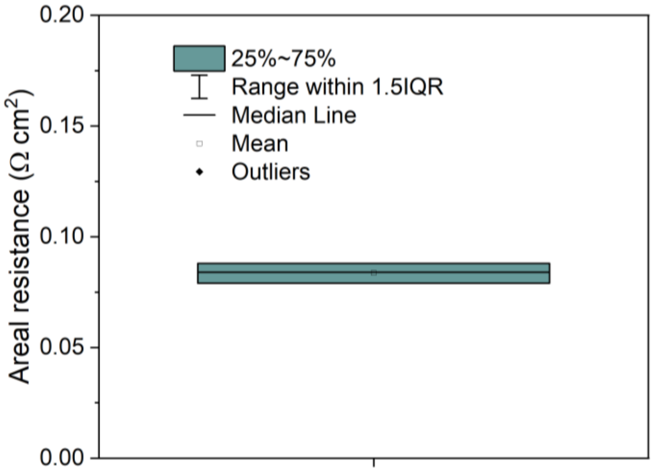

Figure S37. Statistic distribution of high-frequency-resistance (HFR) recorded on Zirfon Perl UTP 220 diaphragm measured before stability test. The diaphragm resistance was obtained by means of EIS measurements performed at the open circuit potential. The diaphragm/membrane resistance was determined from the intercept of the real axis of the Nyquist plot at high frequencies.

The high-frequency-resistance (HFR) recorded on the Zirfon Perl UTP220 demonstrated a low resistance (average HFR: 0.079 Ω cm^2) in 30 wt% KOH at 80 °C. Such resistance is much lower than that based on its thicker type (Zirfon Perl UPT500+) reported previously in literature, which is generally at 0.1–0.15 Ω cm^2 in 30% KOH at 80 °C.[83] The lower resistance realized on the thinner Zirfon diaphragm could ideally provide the higher performance than its thicker candidate, meanwhile provide robust stability (shown in **Figure 6d-f** in the main text), and hence become appropriate to replace the widely used Zirfon Perl UPT500+ diaphragm.

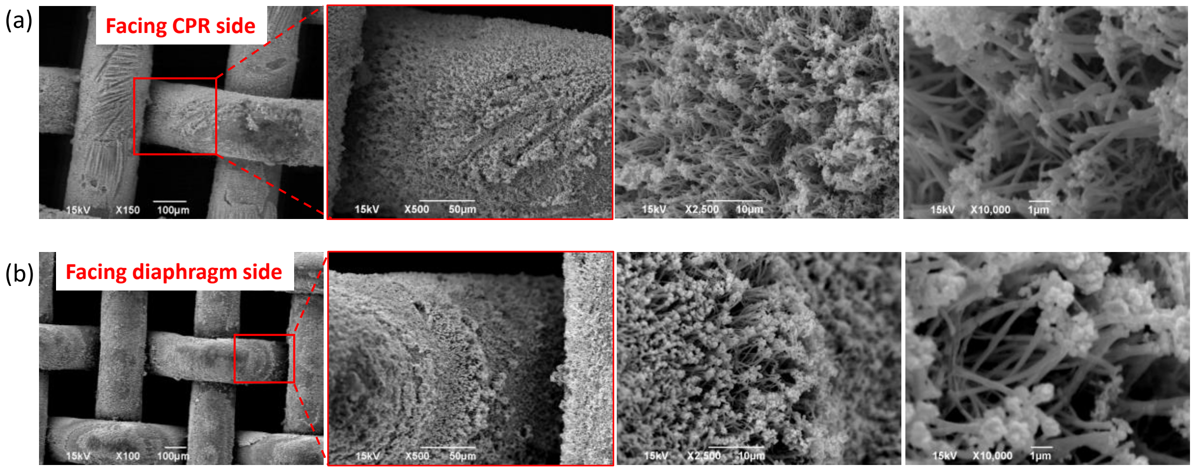

Figure S38. Morphology characterization of electrodes after 200 h of continuous stability test of the corresponding AEL (Ru@Cu-TiO_2/Cu || SSMs): SEM images, at increasing magnification moving from left to right, showing the Ru@Cu-TM surface facing the **a**, the CPR GDL, and **b**, Zirfon Perl UTP220 diaphragm. In some part of the cathode, the peel-off of catalyst layer was caused by its mechanical breakage during the disassembly operation of the AEL.

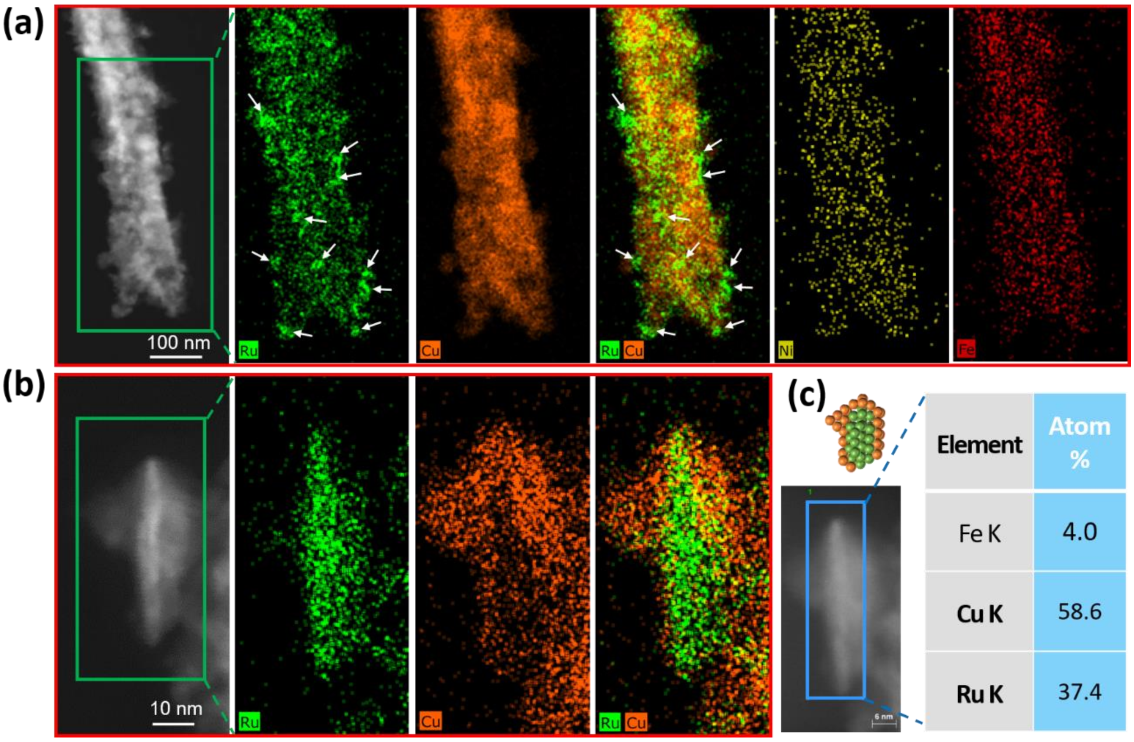

Figure S39. a, HAADF STEM image and the corresponding EDS maps of Ru, Cu, Ni, Fe on a segment of a single nanorod. The overlapped map for Ru-Cu is also given. The white arrows indicate the Ru nanoparticles. **b**, HAADF STEM image and the corresponding EDS maps of Ru, Cu, on a heterostructured particle within the green frame. The overlapped map for Ru-Cu is also given. The composition of the area included in the blue frame is given in **c**, where a sketch shows the hypothetical atom arrangement of the heterostructured particle. Cu (orange) and Ru (green).

We have performed ICP-OES, HAADF-STEM (and EDS) characterizations to analyze the compositional and structural change on the Ru@Cu-TiO_2/Cu electrode after AEL long-term polarization test.

We would like to first clarify that the Ru@Cu-TiO_2/Cu nanorod arrays on the CM surface were unavoidably damaged and peeled off during the disassembly process of the AEL cell after the stability measurement (as shown in **Figure S38**). This could result in a lower total amount of Ru after AEL testing when using quantification techniques like ICP-OES. Indeed, the ICP-OES results indicate that the Ru amount on the Ru@Cu-TiO_2/Cu electrode (the one whose stability results in the AEL test were shown in **Figure 6d**) decreased to 27 μg/cm^2 after the AEL test, compared to the initial loading of 52 μg/cm^2.

However, it is important to note that, although the surface catalyst layer was damaged and thus inevitably resulting in the underestimation of remaining Ru, more than half of the Ru is still retained on the electrode after exposure to harsh operating conditions (200 h at 1 A/cm^2 in 30 wt% KOH at 80°C). In addition, considering that the AEL performance remained stable throughout the 200 h test (as shown in **Figure 6d**), we are confident that Ru demonstrates stability under the working conditions of the AEL.

Interestingly, in contrast to the stability of Ru, we observed a significant decrease in the amount of Ti from 31 μg/cm^2 to 2 μg/cm^2 after the AEL stability test. This observation highlights that Ti is not as stable as Ru under AEL operation conditions. This finding suggests two important conclusions: **i)** The slight performance decay observed in the initial 5 h, as shown in **Figure 6d**, could be partially attributed to the loss of TiO_2. This loss of TiO_2 would slightly worsen the water dissociation step in the

electrochemical reaction. **ii)** The main active site responsible for water dissociation in the Ru@Cu-TiO_2/Cu electrode is Ru.

Furthermore, we also conducted HAADF-STEM imaging and STEM-EDS analyses on the electrode that underwent the AEL stability test (the same electrode used for the ICP-OES measurements mentioned earlier). As shown in **Figure S39a**, we observed a uniform distribution of Ru and Cu throughout the nanorod structure, with Ru appearing in the form of nanoparticles (indicated by the white arrow). Notably, we also identified contaminations of Fe and Ni in the electrode. These contaminations could possibly be attributed to the crossover diffusion of dissolved Fe and Ni from the SSM anode via the diaphragm separator. Although not directly observed in our stability test (**Figure 6d**), the presence of iron and nickel contaminations may impact on the efficiency and durability of AELs. This issue is a common challenge in practical hydrogen production using AELs.[15]

To quantify the mass ratio of Ru/Cu, our focus is on the Ru-Cu nanoheterostructures, which represent the main active species for HER in our study. **Figure S39b** clearly depicts the Ru-Cu nanoheterostructures, where Cu is observed to grow on the surface of Ru. The shape and size of these Ru-Cu nanoheterostructures remained similar to those of the fresh samples (**Figure 1**, **Figure S15**). However, after undergoing the AEL stability test, the Cu species appear to concentrate at the surface of Ru, resulting in a higher Cu/Ru ratio (**Figure S39c**) compared to that of the fresh sample (**Figure S15**, as indicated by the blue star in the heterostructured NP). The enrichment of Cu after the test aligns with XPS results (**Figure S28**), although it's important to note that XPS is a technique sensitive to the entire surface of the nanorod.

Table S7. Comparison between the water splitting performances of our AELs and those of electrolyzers (including PEM and AEM ones) reported in recent literature.

Electrolyzer type	Temperature (°C)	Cathode (mg/cm^2)	Anode (mg/cm^2)	Diaphragm/Membrane	@0.5 A/cm^2 (V)	@1 A/cm^2 (V)	@2 A/cm^2 (V)	Current density at 2V (A/cm^2)	Stability tested	Reference
**AEL**	**80 (30 wt% KOH)**	**Ru@Cu-TiO_2/Cu (Ru loading: 0.052)**	**SS mesh**	**Zirfon Perl UPT 220**	**1.66**	**1.77**	**1.91**	**2.75**	**205 h at 1 A/cm^2, stabilized at ~1.80 V**	**This work**
**AEL**	**80 (30 wt% KOH)**	**Pt/C-CPR (Pt loading: 0.075)**	**SS mesh**	**Zirfon Perl UPT 220**	**1.64**	**1.75**	**1.90**	**2.80**	**/**	
AEL	80 (30 wt% KOH)	Raney-Ni	NiFe-LDH	Zirfon Perl UPT 500	~1.75	~1.95	~2.4	~1.1	N.A.	*Chem. Eng. J.* 428, 131149 (2022)
	80 (30 wt% KOH)	Raney-Ni	NiFe-LDH	80 wt% ZrO_2/5 wt% CNCs	~1.62	~1.75	~1.95	/	N.A.	
	80 (10 wt% KOH)	Raney-Ni	NiFe-LDH	80 wt% ZrO_2/5 wt% CNCs	~1.75	1.9	2.3	~1.2	300 h at 0.6 A/cm^2, stabilized at ~1.84 V	
AEL	80 (30 wt% KOH)	Raney-Ni	NiFe-LDH	Home-made 300 μm-thick Z80 separator (ZrO_2 and PPSU-based film)	/	~1.8	~2.1	~1.8	300 h at 1 A/cm^2, approximately stabilized at ~1.83 V	*J. Membrane Sci.* 616, 118541 (2020)
AEL	80 (24 wt% KOH)	Raney-NiMo	Raney-Ni	Zirfon Perl UPT 500	~1.73	~1.95	~2.4	~1.1	N.A.	*Energy Environ. Sci.*, 12, 3313-3318 (2019)

AEL	80 (30 wt% KOH)	Ni plate	Ni plate	Zirfon Perl UPT 500	~2	/	/	0.5	N.A.	*ACS Sustainable Chem. Eng.* 6, 4829−4837 (2018)
AEL	80 (30 wt% KOH)	Raney-NiMo	Raney-Ni	Zirfon Perl UPT 500	/	~1.9	~2.3	~1.3	N.A.	*J. Electrochem. Soc.* 163, F3197 (2016)
PEM	50 (0.5 M H_2SO_4)	20 wt% Pt/C (0.2)	$Ta_{0.1}Tm_{0.1}Ir_{0.8}O_{2-\delta}$ (0.2)	Nafion 117	~1.61	1.766	1.935	/	500 h at 1.5 A/cm^2 *(~0.2 mV/h decay)*	*Nat. Nanotechnol.* 16, 1371–1377 (2021)
PEM	80 (H_2O)	FeMoS(mw) (4)	Ir black (2)	Nafion 212	1.77	1.85	/	/	24 h at cycling between 0.05 and 0.5 A/cm^2 (*~1.9 mV/h decay*)	*ACS Catal.* 10, 14336−14348 (2020)
PEM	80 (H_2O)	60 wt% Pt/C (0.5)	IrO_2@TiO_2 (Ir-loading: 0.4)	Nafion N212 or N115	~1.6	1.67	~1.78	~4.3	N.A.	*Appl. Catal. B: Environ.* 269, 118762, (2020)
PEM	60 (H_2O)	Pt/C (1.5)	$Y_{1.75}Ca_{0.25}Ru_2O_7$ (4.1)	Nafion 212	~1.58	~1.67	/	/	16.7 h at 0.2 A/cm^2 *(~9 mV/h decay)*	*Appl. Catal. B: Environ.* 260, 118176 (2020)
PEM*	60 (H_2O)	Pt/C (1.5)	IrO_2 (3)	Nafion 212	~1.64	~1.75	/	/	N.A.	
PEM	80 (H_2O)	Pt black (3)	$IrRuO_x$ (3)	Nafion 115	/	~1.67	1.84	/	N.A.	*Nano Energy* 47, 434–441 (2018)
PEM	80 (H_2O)	Pt/C (0.5)	$Ir_{0.7}Ru_{0.3}O_x$ (1.8)	Nafion 115	~1.54	1.66	~1.84	/	4 h at 0.2 A/cm^2	*Adv. Energy Mater.* 9, 1802136 (2018)
PEM	38 (H_2O)	Pt	Ir	Nafion 212	~1.7	~1.8	1.97	/	N.A.	*Energy Environ. Sci.* 10, 2521-2533 (2017)

PEM*	80 (H_2O)	Pt/C (2)	IrO_2 (2.2)	Nafion 115	~1.55	1.64	1.83	/	N.A.	*ACS Catal*. 6, 2626 (2016)
PEM	80 (H_2O)	Pyrite FeS_2 (5)	IrO_2 (2.2)	Nafion 115	~2	2.1	2.23	0.5	N.A.	
PEM	80 (H_2O)	40 wt% Pt/C (1)	IrO_x-Ir (1)	Nafion 212	~1.57	~1.64	~1.77	/	100 h at 2 A/cm^2, fluctuated between 1.78-1.8 V	*Angew. Chem. Int. Ed.* 55, 742-746 (2016)
PEM	80 (H_2O)	46.7 wt% Pt/C (~0.35 mg Pt)	IrO_2/TiO_2 (75 wt% iridium) (~2 mg Ir)	Nafion 212 (50 μm)	~1.52	1.57	~1.65	>6	N.A.	*J. Electrochem.* Soc. 163, F3179 (2016)
AEM	60 (1M KOH)	$MoNi_4/MoO_2$	Ni_2P @ $FePO_xH_y$	Sustainion X37-50	~1.75	1.84	/	/	72 h at 1.75 V, decrease from 0.5 to ~0.45 A/cm^2	*Appl. Catal. B: Environ*. 306, 121127 (2022)
AEM	45 (1 M KOH)	40 wt% Pt/C (1 mg Pt)	etched copper−cobalt oxide (~32 mg)	Sustainion X37-50T	1.686	~1.78	/	/	500 h at 0.5 A/cm^2 (0.17 mV/h)	*ACS Energy Lett*. 7, 2576−2583 (2022)
	45 (1 M KOH)	40 wt% Pt/C (1 mg Pt)	etched copper−cobalt oxide (~32 mg)	polycarbazole-based anion exchange membrane (QPC-TMA)	1.679	~1.78	/	/	500 h at 0.5 A/cm^2 (12 μV/h)	
AEM	50 (1 M KOH)	Raney NiMo (25.2)	$Ni(OH)_2$-Fe (4)	Tokuyama A201 (28 μm)	~1.7	~1.85	2.046	~1.8	486 h at 1 A/cm^2, approximately stable between 1.92 V and 1.85 V	*ACS Appl. Energy Mater*. 5, 2221–2230 (2022)
AEM	90 (6M KOH)	Pt/C (1.5)	IrO_2 (1.5)	NPBI^ ion-solvating membrane	~1.57	~1.69	~1.9	~2.5	298 h at 0.5 A/cm^2, fluctuated between 1.66-2 V	*J. Membrane Sci.* 643, 120042 (2022)

AEM	50 (0.3 M KOH)	PtNi (3.0 ± 0.1)	IrO_x (3.0 ± 0.1)	XION™ Composite-72–10CL-30 μm	/	1.79	/	/	720 h at 1A/cm^2, almost stable at 1.8 ± 0.05 V	*Electrochim. Acta* 409, 140001 (2022)
AEM	50 (1M KOH)	Pt/C	NiFeV LDH (3.95)	Sustainion X37-50	~1.56	~1.65	~1.79	/	100 h at 0.5 A/cm^2 *(~2 mV/h decay)*	*Small* 17, 2100639 (2021)
AEM	60 (H_2O)	PtRu/C (Pt, Ru loading: 2,2)	IrO_2 (~4)	HTMA-DAPP*(~50 μm)	~1.65	1.79	/	/	24 h at 0.2 A/cm^2, stable for the first 8 h, then increase by 100 mV	*ACS Appl. Mater. Interfaces* 13, 50957−50964 (2021)
AEM	80 (1 M KOH)	46.6% Pt/C (0.5), PFBP-14 ionomer	IrO_2 (2), PFTP-8 ionomer	PFTP-13 ♦	/	~1.6	~1.67	7.68	N.A.	*Energy Environ. Sci.*, 14, 6338 (2021)
AEM	60 (1 M KOH)	46.6% Pt/C (0.5), PFBP-14 ionomer	IrO_2 (2), PFTP-8 ionomer	PFTP-13	~1.61	~1.67	~1.78	~5.2	1100 h at 0.5A/cm^2, fast increased to ~2.3V, then fluctuated between 2.2-2.0 V	
AEM	90 (H_2O)	47% Pt/C (0.94 mg Pt)	fluoride-incorporated Fe_xNi_yOOH on Ni foam (4.8)	poly(aryl piperidinium) hydroxide exchange membrane (PAP-TP-85, 20 μm)	~1.65	~1.8	/	/	70 h at 0.5 A/cm^2 *(~1.81 mV/h decay)*; 160 h at 0.2 A/cm^2 *(~0.56 mV/h decay)*	*ACS Catal.* 11, 264−270 (2021)
AEM	42-45 (1 M KOH)	Pt/C (1)	$Ni_{0.75}Fe_{2.25}O_4$	Sustainion X37-50	~1.65	1.75	1.9	/	21 h at 0.5 A/cm^2 *(~9.5 mV/h decay)*	*Chem. Eng. J.* 420, 127670 (2021)
AEM*	42-45 (1 M KOH)	Pt/C (1)	IrO_2 (4)	Sustainion X37-50	~1.67	1.84	/	/	N.A.	

AEM	85 (H_2O)	PtRu/C (50wt% Pt and 25wt% Ru) (2)	Ni_2Fe_1 (3)	HTMA-DAPP*(26 μm)	~1.54	~1.61	~1.71	~3.5	10 h at 0.2 A/cm^2, rapidly increased to ~2.3 V	*Nat. Energy* 5, 378–385 (2020)
AEM	60 (1 M KOH)	PtRu/C (50wt% Pt and 25wt% Ru) (2)	IrO_2 (2.5)	HTMA-DAPP*(26 μm)	~1.8	~1.95	/	~1.15	100 h at 0.2 A/cm^2, rapidly increase to 2.1 V within initial 3 h, then become stable	
AEM	70 (1 M KOH)	60 wt.% Pt/C (0.4 mg Pt)	60 wt.% PtRu/C (0.4 mg Pt-Ru metal)	AEM based on quaternised poly-carbazole (QPC-TMA) (50 μm)	~1.55	~1.62	~1.74	4.5	10000 s at 1.6 V cell voltage, current density decreased by ~50 %	*Energy Environ. Sci.*, 13, 3633-3645 (2020)
AEM	45 (1 M KOH)	40 wt% Pt/C (1)	IrO_x (4)	Sustainion X37-50	/	~1.75	~1.87	/	64 h at 0.5 A/cm^2	*Appl. Catal. B: Environ.* 278, 119276 (2020)
AEM	80 (1 M KOH)	Pt/C (1.3)	NiFe-LDH (2.5)	Sustainion X37-50	~1.52	1.59	/	/	6 h at 1 A/cm^2 *(~3.3 mV/h decay)*	*ACS Catal.* 10, 1886-1893 (2020)
AEM*	60 (1 M KOH)	Pt/C (1.2)	IrO_x (1.9)	Sustainion X37-50	~1.55	1.67	/	/	6 h at 1 A/cm^2 *(~10 mV/h decay)*	
AEM	50 (1 M KOH)	40 wt% Pt/C (1)	$Cu_{0.5}Co_{2.5}O_4$ (10)	Sustainion X37-50	~1.65	~1.74	/	/	100 h at 0.5 A/cm^2 *(~0.6 mV/h decay)*	*J. Mater. Chem. A* 8, 4290–4299 (2020)
AEM	20 (1 M KOH)	NiMo-NH_3/H_2 (3)	Fe-NiMo-NH_3/H_2 (3)	Sustainion X37-50	~1.66	1.77	/	/	25 h at 0.5 A/cm^2, Increased from ~1.66V to ~1.72V	*Adv. Energy Mater.* 10, 2002285 (2020)

AEM	80 (1 M KOH)	NiMo-NH_3/H_2 (3)	Fe-NiMo-NH_3/H_2 (3)	Sustainion X37-50	1.52	1.57 V	/	/	N.A.	
AEM	50 (H_2O)	Pt black (3)	NiCoOx:Fe (3)	FAA-3 (FumaTech)	~2.1	~2.45	/	~0.37	3 h at 0.2 A/cm^2 *(~150 mV/h decay)*	*ACS Catal.* 9, 7–15, (2019)
AEM	30 (0.1 M KOH)	40 wt% Pt/C (1)	$Cu_{0.81}Co_{2.19}O_4$	Fumasep FAA-3-PE-30	~1.87	/	/	/	100 h at 0.1 A/cm^2 *(~0.2 mV/h decay)*	*ACS Appl. Mater. Interfaces* 10, 38663-38668 (2018)
AEM	60 (1 M KOH)	NiFeCo (2)	$NiFe_2O_4$ (2)	Sustainion 37-50	~1.8	1.9	/	~1.7	1950 h at 1 A/cm^2, almost stable at 1.9-1.92 V	*Int. J. Hydrogen Energy* 42, 29661–29665 (2017)

Note: * Commercial PGM (*e.g.*, Pt, Ir and Ru)-based ELs; † ccm: Cubic Centimeter per Minute (cm^3/min, equals mL/min); ★hexamethyl trimethyl ammonium-functionalized Diels-Alder polyphenylene; ^ Poly[2,2'-(1,4-naphthalene)-5,5'-bibenzimidazole]; ♦ poly(fluorenyl-co-terphenyl piperidinium-13).

Carbon paper (CPR) was adopted as GDL at the cathode side in our AEL assembly, while no GDL was used at the anode side.

The cell voltage values with the symbol (~) are extracted from their corresponding polarization plots.

The operating pressure of electrolyzers is not always mentioned.

We had no bias and tried to collect some literature on AELs based on PGM catalysts for comparison. Interestingly, **we found that PGM-based catalysts were rarely reported in AEL configuration**. In many cases they have been used in AEMEL and we have already included many of them in **Table S7**. Most likely, this is due to the reason that AEMEL has been widely regarded as the next generation technology to replace the current one based on AEL. Nevertheless, the AEL still has its big advantage (e.g., much cheaper CAPEX, robust stability, matured technology, etc) and would still play significant role in the coming short-medium future. Hence, we are keen to compare the performance of AEL based on our developed Ru@Cu-TiO_2/Cu and benchmark Pt/C-CPR (75 μgPt/cm^2). Most likely, this is due to the reason that AEMEL has been widely regarded as the next-generation technology to replace the current one based on AEL. Nevertheless, **the AEL still has its big advantage** (e.g., much cheaper CAPEX, robust stability, matured technology, etc) and would still play a main/significant role in the upcoming short-medium future.

Calculation of mass and price activity

The commercial Ni-based electrode dedicated for alkaline water electrolzers is purchased from Fuel Cell Store.[33] Where the Raney Ni catalyst is pasted firmly on a thin Ni foam substrate to get a dense layer. The mass loading of Ni catalyst is ca. 150 mg/cm^2 according to our careful measurement on the nickel catalyst layer broken away from the Ni foam substrate.

To simplify the comparison between their HER activities, we only consider the key part of the catalyst that affects the price, while the substrate is not taken into account.

Mass activity

The mass activities of the cathodes were calculated by dividing the current density, recorded at the overpotential of -100 mV (vs. RHE), by the mass loading of PGM metals (Ru or Pt):

$$j_{\mathrm{mass}}^{\mathrm{Ru@Cu-TiO2/Cu}} = \frac{381\ (\mathrm{mA/cm^2})}{0.052\ (\mathrm{mg/cm^2})} = 7330\ \mathrm{A/mg}\ = 7.33\ \mathrm{A/mg}\,;$$

$$j_{\mathrm{mass}}^{\mathrm{Pt/C-CPR}} = \frac{108\ (\mathrm{mA/cm^2})}{0.1\ (\mathrm{mg/cm^2})} = 1080\ \mathrm{A/g}\ =\ 1.08\ \mathrm{A/mg}\,;$$

$$j_{\mathrm{mass}}^{\mathrm{commercial\ Ni\ electrode}} = \frac{7.9\ (\mathrm{mA/cm^2})}{150\ (\mathrm{mg/cm^2})} = 0.053\ \mathrm{A/g}\ =\ 0.000053\ \mathrm{A/mg}$$

Price activity

The price activities of the cathodes were simply computed by dividing their mass activity, by the unit price of Ru or Pt.[84] For the commercial Ni-based electrode, we referred to the average price of Raney Ni in the market from the website of alibaba.com.[85]

$$j_{\mathrm{price}}^{\mathrm{Ru@Cu-TiO2/Cu}} = \frac{7330.33 * 1000\ (\mathrm{A/g})}{6.88\ (\$/\mathrm{g})} = 1065.4\ \mathrm{A/US\$}$$

$$j_{\mathrm{price}}^{\mathrm{Pt/C-CPR}} = \frac{1080.08 * 1000\ (\mathrm{A/g})}{34.36\ (\$/\mathrm{g})} = 31.4\ \mathrm{A/US\$}$$

$$j_{\mathrm{price}}^{\mathrm{commercial\ Ni\ electrode}} = \frac{0.053\ (\mathrm{A/g})}{0.0233\ (\$/\mathrm{g})} = 2.27\ \mathrm{A/US\$}$$

Estimation of operating cost for H_2 production in our AELs

In this section, the operating cost (only considering the electricity consumed by electrolyzer component) for the H_2 production in our AELs were calculated using the experimental data acquired during the stability test of our Ru@Cu-TiO_2/Cu || SSMs AEL using Zirfon Perl UTP220 diaphragm. In particular, an average voltage recorded in the stability test on the AEL operating at 1 A/cm^2 for 200 h, rather than a single polarization measurement, was used to provide a more reliable result. Therefore, a voltage of 1.80 V was considered for the 1 A/cm^2 operation of our AEL.

Hence:

Rate of H_2 generation (*a*) on 1 cm^2 electrode at <u>1 A/cm^2</u>

$$a = \frac{j}{F \times n} \times N_A$$

$$= \frac{(Current\ density)}{F \times 2} \times 6.022 * 10^{23}$$

$$= \frac{1}{96485 \times 2} \times 6.022 * 10^{23}$$

$$= 3.12 \times 10^{18}\ H_2\ \text{molecules}/(\text{cm}^2 \times \text{s})$$

For <u>200 h, 1cm^2</u> AEL can produce the following H_2 mass and volume:

$$\text{Mass}\ (H_2) = \frac{a \times S_{electrolyzer} \times t}{N_A} \times M_w$$

$$= \frac{(H2\ production\ rate) \times Electrolyzer\ area \times Time}{6.022 * 10^{23}} \times (\text{Molar mass of } H_2)$$

$$= \frac{3.12 * 10^{18} \times 1 \times (200 * 3600)}{6.022 * 10^{23}} \times 2.016$$

$$= 7.46\ \text{g}\ H_2$$

Considering only the electricity consumed by electrolyzer component, the operating cost of H_2 per kilogram of H_2 is (see TEA analysis in the subsequent section):

Operating cost (H_2/kg) = energy consumption × electricity cost

$$= \frac{1\ \text{A/cm}^2 \times 1\ \text{cm}^2 \times 1.8\ \text{V} \times 200\ \text{h}}{1000 \times 0.00746\ \text{kg}} \times \underline{\$0.02/\text{Kw h}}$$

$$= 48.26\ \text{Kw h / kg}\ H_2 \times \$0.02/\text{Kw h}$$

$$= \$\ 0.97\ /\ \text{kg}\ H_2$$

Note: Assuming the unite price of electricity is $0.02/Kw h

Techno-economic analysis (TEA)

Additional experimental and calculations details

In the following, all the calculations and assumption made in the annexed Excel Spreadsheet are sheet-by-sheet described and commented. All parameters fixed and assumptions made are gathered in **Tables S7-10**.

"Description": reports the rationale and boundaries of the TEA.

"Single cell": reports the calculation of the unitary cost (\$ per 1 cm^2) of a diaphragm/electrodes package (DEP) based on the technology reported by the present paper. The DEP includes the cost of cathode, anode and diaphragm, as detailed in the following:

Cathode: comprises the active phase, *i.e.*, Ru, the metallic support, *i.e.*, CM, and the sputter-coated layers of Cu and Ti. In addition, the manufacturing costs related to the synthetic route has also been considered, including the price of used precursors, process water and energy consumption.

Raw materials/components: The unitary costs of each raw material included in the manufacturing of the cathode were retrieved from different websites reporting updated market prices of metals. The exact Ru and Ti loadings were determined by ICP-OES, as described in the main text. The actual unitary cost of the raw materials/components has been calculated by multiplying the mass loading (or area, in the case of the support, CM) per cm^2 by the price of the component itself.

Synthetic route: the cost of manufacturing has been calculated as the sum of the cost of each individual synthetic step, as detailed in the following.

Synthesis of the skeleton of 3D $Cu(OH)_2$ NRs on CM: the cost of the chemical bath used to grow $Cu(OH)_2$ NRs on CM has been calculated according to the volume of process water and mass of reagents (i.e. ammonium persulfate and sodium hydroxide) used. Normalization by a factor that accounts for the ratio between the area of CM immersed and the volume of the chemical bath, allowed us to calculate the unitary (per cm^2) manufacturing cost of this synthetic step.

Sputtering Cu and Ti layers onto the surface of $Cu(OH)_2$ NRs: since it is quite complex to rationalize, the cost associated with the sputtering of Cu and Ti onto the $Cu(OH)_2$-functionalized support has been neglected. On the other hand, the cost of the raw materials (metal) employed has been considered in the cathode cost calculation (see previous paragraph "*Raw materials/components*").

In-situ $Cu(OH)_2$ electroreduction and following Ru electrodeposition to obtain the final Ru@Cu-yTiO_2/xCu: calculations have been split as for the actual procedure, made of two steps. For $Cu(OH)_2$ electroreduction, process water and NaOH costs have been included in addition to the energetic input required for the reduction (details available in the Excel spreadsheet). Similarly, the energetic input has been considered also for the Ru electrodeposition, while the cost of the Ru(IV) precursor has been neglected to avoid

double-counting with the Ru cost evaluated in the "*Raw materials/components*" section (details available in the Excel spreadsheet).

Anode: constituted by 5 stacked Type 316 SSM pieces, each one sized 5 cm^2. Its price was retrieved from its supplier since it is a commercial product.

Diaphragm: constituted by Zirfon PERL UTP 220. Being Zirfon PERL UTP 220 a commercial product, its price was also retrieved from the supplier.

"CAPEX – Single AEL cells – Std & ours": reports the calculations of the CAPEX of the ideal 1 MW-scale AEL plant based on the DEP configurations studied in the present work. At first, the CAPEX of a generic 1 MW-scale AEL plant was estimated from the literature data.[15,16] as already reported in previous studies.[86] Then, following the cost breakdown of a generic MW-scale AEL plant reported by IRENA[15] and assuming AEL performances to meet those reported by the Korean Institute of Energy Research,[16] the CAPEX was split into stack, DEP and Balance of Plant (BoP) costs and relative subcomponents.
Setting the size of the AEL single cells to 700 cm^2 and assuming the cell manufacturing costs to match those retrieved for the previously described generic 1 MW-scale AEL plant, the CAPEX associated with a single cell of an ideally up-scaled AEL based on our DEP technology has been calculated. Note that such CAPEX estimation was made under the assumption that cathode and anode costs scale linearly with plant size.
Finally, a capital recovery factor (CRF), used in the following sheets to calculate the annual from the total CAPEX of the plant, has been estimated according to the following equation[16]:

$$\mathrm{CRF} = \frac{\mathrm{i_{Rate}} \times (1 + \mathrm{i_{Rate}})^{\mathrm{n}}}{(1 + \mathrm{i_{rate}})^{\mathrm{n}} - 1}$$

where i_{rate} is the discount rate and n is the AEL plant lifetime (in years).

"CAPEX & OPEX Calculations": reports the electrochemical data (*i.e.*, current-voltage relationships) collected on a 5 cm^2 AEL single cell operating under industrially relevant conditions and the related energy efficiencies, based on the hydrogen higher heating value (energy $efficiency_{HHV}$).
The ideal up-scaling procedure has been carried out by setting a 1 MW total net power of the system for each configuration (i.e. for each couple of current/voltage). Based on this assumption, the amount of H_2 (in moles and kilograms) produced every year by the ideal plant can be calculated and is the same for different operative current density. On the other hand, different operative current densities result in a different gross power of the system and therefore in a variable number of cells required to meet the 1 MW net power requirement. Obviously, the number of cells/stacks of the AEL, despite the fixed net power of the latter, impact on both CAPEX and OPEX (and therefore on the Levelized Cost of Hydrogen, LCOH) of the overall system.

The total CAPEX of ideal 1 MW-scale AELs has been computed from the number of cells and the CAPEX breakdown reported in the dedicated spreadsheet ("CAPEX – Single AEL cells – Std & ours"). Annual CAPEX has been retrieved from the total one by multiplying the latter by a proper CRF ("CAPEX – Single AEL cells – Std & ours").

For what concerns OPEX, the main operating expenses *i.e.*, the electricity fed to the electrolyzer, the process water consumed, labor, maintenance and ancillary costs, were all considered. The OPEX related

to electricity and process water, which are dependent on the electrolyzer performance, were calculated according to the gross power of the system and the water annual consumption, respectively.
In details, the $OPEX_{Electricity}$, related to the energy consumption of the actual electrolysis process, was calculated according to the following equations:

$$I_{Total} = i \times A_{Single\ cell-Ideal\ 1\ MW-scale\ AEL} \times n_{Cells\ per\ stack} \times n_{Stacks\ per\ system}$$

$$P_{AEL\ (gross)} = I_{Tot} \times E_{Cell} \times t_{Annual\ AEL\ operation}$$

$$OPEX_{Electricity} = P_{AEL\ (gross)} \times C_{Electricity}$$

where I (i) indicates the current (current density) flowing through the electrolyzer, A stands for area, E for voltage, P for power, t for time and C for cost.
The expenses related to process water consumption have been estimated through the following equations:

$$m_{H2O\ consumed\ (per\ year)} = m_{Produced\ H2\ (per\ year)} \times m_{Average\ H2O\ consumption\ per\ kg\ of\ H2}$$

$$OPEX_{H2O} = m_{H2O\ consumed\ (per\ year)} \times C_{H2O}$$

where m stands for mass.
On the other hand, labor, maintenance and other ancillary OPEX contributions were calculated as percentages of the total CAPEX of the whole system.
All the calculations reported in this sheet have been carried out using both H_2 higher heating value (HHV) and hydrogen lower heating value (LHV).

"Annual H2 productivity-1 MW net power": reports the total current flowing through the AEL and the amount of yearly produced hydrogen for each operative condition, all up-scaled to a 1 MW scale. In virtue of the previously made assumption (1 MW net power), these values are the same for each operative condition.

"LCOH": reports the final calculation of the production cost of H_2, starting from CAPEX and OPEX values and H_2 annual productivity for each operative condition, allowing to spot the most profitable one. According to reports on currently operative AELs,[15] the energy consumption for the actual electrolytic process accounts only for the 50% of the overall energy fed to the whole system, with BoP auxiliaries (gas and liquid circulation, gas compression…) requiring a similar energy fed. Therefore, the annual OPEX has been calculated doubling the $OPEX_{Electricity}$. Refer to the dedicated paragraph in the Experimental Section and to the Excel spreadsheet for additional LCOH calculation details.

Fixed parameters and assumptions made throughout the TEA

In the following, all the parameters that were fixed and assumptions that were made throughout the TEA are gathered in the form of tables (**Tables S7-10)**. All the assumptions made and parameters retrieved from literature are closely related to those reported in previous papers[86] and are provided here only for the sake of clarity. In addition, the same data are reported in the annexed Excel Spreadsheet.

In-depth techno-economic discussion

A preliminary TEA has been carried out to evaluate the LCOH produced by an ideal 1 MW (net power)-scale AEL implementing the single cell technology designed in this work. In particular, the impact of the

operative current density/cell voltage, has been studied. With the constrain of a 1 MW total net power of the system, lower operative current densities result in a larger number of AEL cells/stacks to achieve the desired power and *vice versa*. Thus, the operative current density/cell voltage influences both the CAPEX and OPEX of the whole system.

As expected, the total CAPEX related to the deployment of the plant decreases exponentially when moving from low to high current densities, because of the diminished number of cells required to achieve the target 1 MW net power (**Figure S40**). Obviously, the same trend is observed for the annual CAPEX (**Figure 7** in the main text**)** as it is retrieved from a simple linear depreciation model based on a fixed capital recovery factor (refer to the previous section).

On the other hand, OPEX has a more complex trend (**Figure 7** in the main text), which reflects the multiple dependence of its constituting entries: for example, $OPEX_{Electricity}$ depends mainly on the electrochemical performance of the systems and on the cost of electricity,[15] while labor and maintenance are generally computed as percentages of the system CAPEX.[15] The OPEX indeed decreases exponentially when passing from 100 to *ca*. 300 mA/cm^2 and then linearly increases throughout the whole range of current density explored, although with a modest slope (**Figure 7** in the main text). Such trend may be rationalized analyzing the variation of CAPEX and energy efficiency with the operative current density. Indeed, for low current densities, large CAPEX increases the influence of labor, maintenance and auxiliaries OPEX entries, transposing the exponential trend observed for the CAPEX (**Figure 7** in the main text, **Figure S40**) to the OPEX. With the increase of the operative current density (and flattening of the CAPEX curve), the main factor defining the OPEX becomes the actual electrolysis (i.e., the electric energy consumption, $OPEX_{Electricity}$); the moderate increment of OPEX toward higher current densities is indeed related to the slight reduction in the voltage and energy efficiency of the system operating at sustained H_2 productivity.

The LCOH dependence on the operative current density is a hybrid of CAPEX and OPEX trends (**Figure 7** in the main text), as suggested by the equation used in its calculation (refer to the previous section and the annexed **Excel Spreadsheet**). Consistently, LCOH reaches minimum when the AEL is operated at 500 mA/cm^2 (@ 1.66 V single cell voltage), achieving a value as low as US$2.12/$kg_{H2}$ (additional key assumptions made: H_2 HHV-based calculations and plant lifetime of 30 years). Noticeably, the LCOH is lower than US$2.50/$kg_{H2}$ throughout the whole current density (**Figure 7** in the main text and **Excel spreadsheet**), evidencing that the Ru-based cathodes and the overall technology reported herein are promising catalytic systems for massive H_2 production. In addition, considering a shorter plant life (*i.e*., 10 years, **Figure S41**) does not heavily affect the LCOH, which even in this case lingers below the US$2.50/$kg_{H2}$, hitting a minimum of US$2.22/$kg_{H2}$ (**Figure S41** and **Excel spreadsheet**).

Focusing on the most profitable operative conditions (500 mA/cm^2 @ 1.66 V single cell voltage), the CAPEX and OPEX breakdowns come in handy to pinpoint the main factors contributing to the LCOH. Starting from the CAPEX breakdown (**Figure S42**), it is worth noticing that the main contributors to the final cost of the Ru@Cu-TiO_2/Cu NR cathodes are the metals used (*ca*. 75% of the related CAPEX), with manufacturing costs accounting only for the remaining 25%. In particular, more than half of the manufacturing costs are allocated to the $Cu(OH)_2$ electroreduction, the longest and more energy-demanding step of the process. It is also interesting to notice that, conversely to what might be expected, Ru accounts only for the 25% of the CAPEX associated with cathode components, with the support (*i.e*., copper mesh) covering the remaining share. This result indicates that when the loading of PGMs (in this case, Ru) is minimized by a proper catalyst design, neither PGMs nor manufacturing cost substantially impact on the CAPEX. In fact, the overall cost of the Ru-based cathode represent only the 2.5% of the final DEP (**Figure S42**) against an

estimated 4% for the typical Ni-coated perforated stainless steel plates (**Figure S43**) that represent the electrode benchmarks for large-scale AELs.[15] Extending the CAPEX breakdown comparison between our ideal 1 MW-scale AEL plant (**Figure S42**) and an average of the currently operative AELs worldwide (**Figure S43**),[15] it can be noticed that the larger number of cells of our AEL entails a larger impact of the DEP packages at stack level. In turn, such discrepancy leads to a higher system CAPEX the AEL based on our technology (*ca*. 20% higher than current AELs).

Moving to the OPEX breakdown, the pie chart in **Figure S44a** depicts an expected dominance of the energy-driven OPEX entries: $OPEX_{Electricity}$ (encompassing both the energy fed to the electrolyzer and the BoP powering) indeed accounts for the 92% of annual OPEX. Moreover, the comparison of the annual OPEX with the corresponding CAPEX (OPEX:CAPEX *ca*. 10:1, **Figure S44b**) further supports the common sense that achieving better electrochemical performance at the expense of a moderate increase in the CAPEX turns out in more profitable AELs. [87]

Table S8. CAPEX-related parameters that have been set/retrieved from different sources throughout the TEA.

Parameters for single cell CAPEX calculation			
DEP components[a]	Ruthenium	19.93	$ g^{-1}
	Cu mesh	27	$ m^{-2}
	Cu (sputtered)	8.06	$ kg^{-1}
	Ti (sputtered)	28	$ kg^{-1}
	SSM	11.20	$ m^{-2}
	Zirfon Perl UTP 220	380	$ m^{-2}
Reactants for cathode manufacturing[a]	Ammonium persulfate	1.15	$ kg^{-1}
	Sodium hydroxide	0.75	$ kg^{-1}
	Process water[88]	0.0014	$ L^{-1}_{H2}
Electrode area	Lab-scale AEL	5	cm^2
	Ideal 1 MW-scale AEL	700	cm^2
Parameters for breaking down the cost of a generic 1 MW-scale AEL plant			
System CAPEX breakdown	Average stack cost (1 MW)[16]	270	$ kW^{-1}
	Stack CAPEX share[15]	45	% of total CAPEX
	BoP CAPEX share[15]	55	% of total CAPEX
System operative parameters[16]	Operative voltage	1.8	V
	Operative current density	1	A cm^{-2}
	Electrode area	700	cm^2
	Single cell power	1260	W
	n_{Cell} (cells per stack)	200	cells
	n_{Stacks} (stacks per system)	5	stacks
	Gross system power	1.26	MW
	Energy $efficiency_{HHV}$	82	%
	Net system power	1.03	MW

[a] *Check the Excel spreadsheet for the price sources;*

Table S9. OPEX-related parameters assumed in the TEA.

$OPEX_{Process\ water}$	Process water usage[15]	10	L kg^{-1}_{H2}
	Process water cost[16]	0.0014	\$ L^{-1}_{H2}
Other OPEX entries[15]	$OPEX_{Labour}$	0.3	% of total CAPEX
	$OPEX_{Maintainance}$	2.5	% of total CAPEX
	$OPEX_{Ancillary}$	1	% of total CAPEX
-	Time of operation	8400	h $year^{-1}$

Table S10. Financial parameters assumed in the TEA.

Interest rate	4.5	%
Plant lifetime[a]	30	years
Capital Recovery Factor	0.061	
Electricity cost[15]	0.02	\$ kWh^{-1}

[a] *Average plant lifetime for MW-scale AELs is ca. 30 years.*[15]

Table S11. Electrochemical and process-related parameters assumed in the TEA.

Faradaic efficiency	100	%
Number of exchanged electrons	2	mol_{e-} mol^{-1}_{H2}
Faraday's constant	96485	C mol^{-1}_{e-}
H_2 molar mass	2.016	g mol^{-1}
HHV	141.7	kJ g^{-1}_{H2}
LHV	120.0	kJ g^{-1}_{H2}

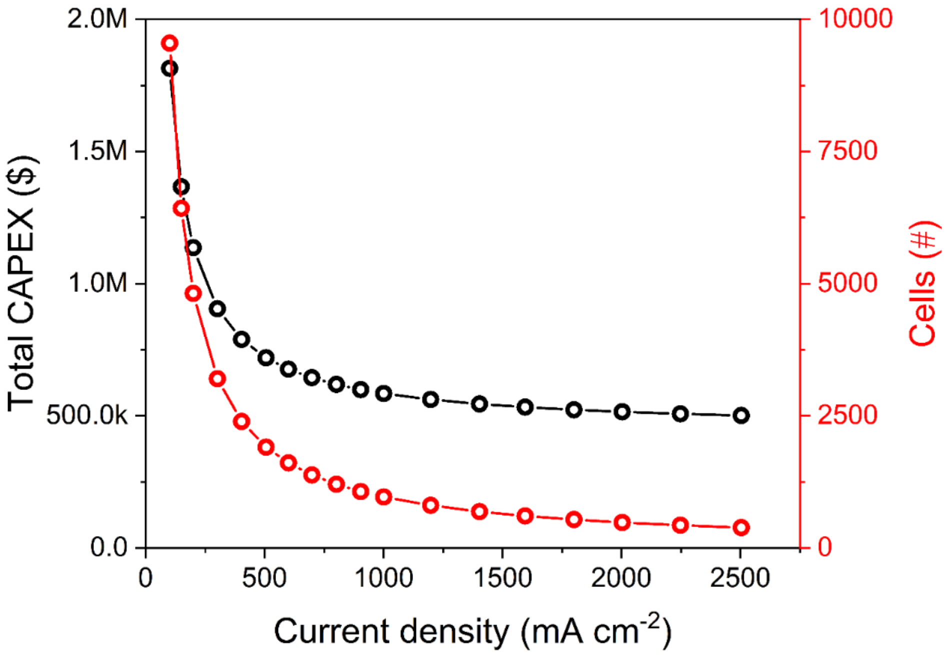

Figure S40. Total CAPEX and cells number as a function of the operative current density for an ideal 1 MW-scale AEL based on the DEP technology reported in the work. All calculations have been carried out using the H_2 HHV.

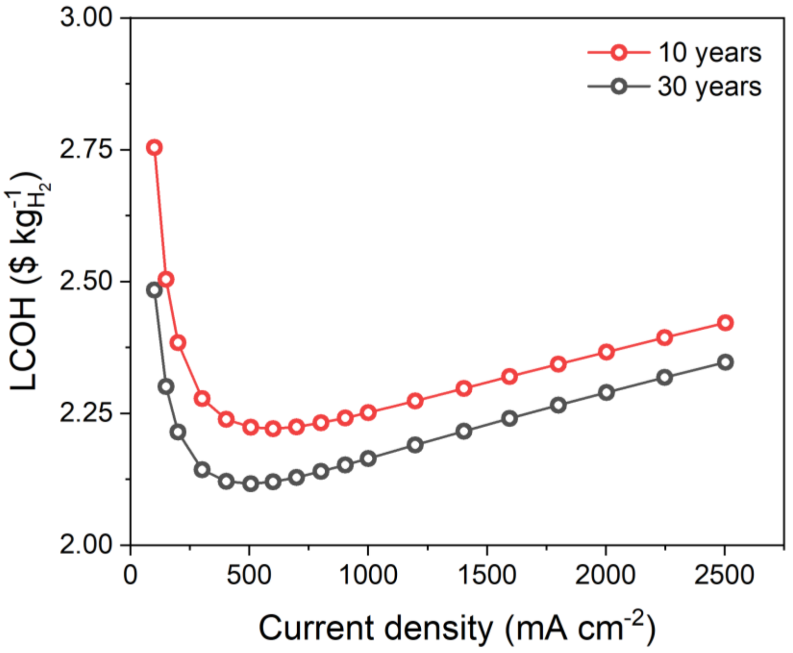

Figure S41. Trends of LCOH versus operative current density of an ideal 1 MW-scale AEL based on the DEP technology reported in this work for different plant lifetimes. All calculations have been carried out using the H_2 HHV.

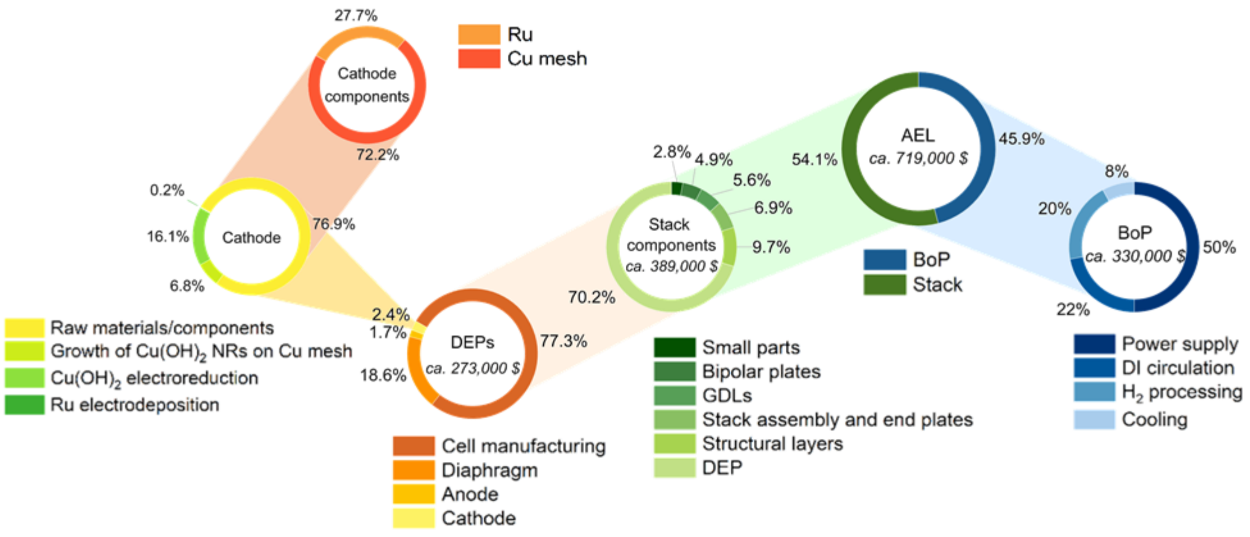

Figure S42. CAPEX breakdown of an ideal 1 MW-scale AEL based on the DEP technology reported in this work. All data are related to an AEL plant of 30-year lifetime operating at 500 mA/cm^2 current density. All calculations have been carried out using the H_2 HHV.

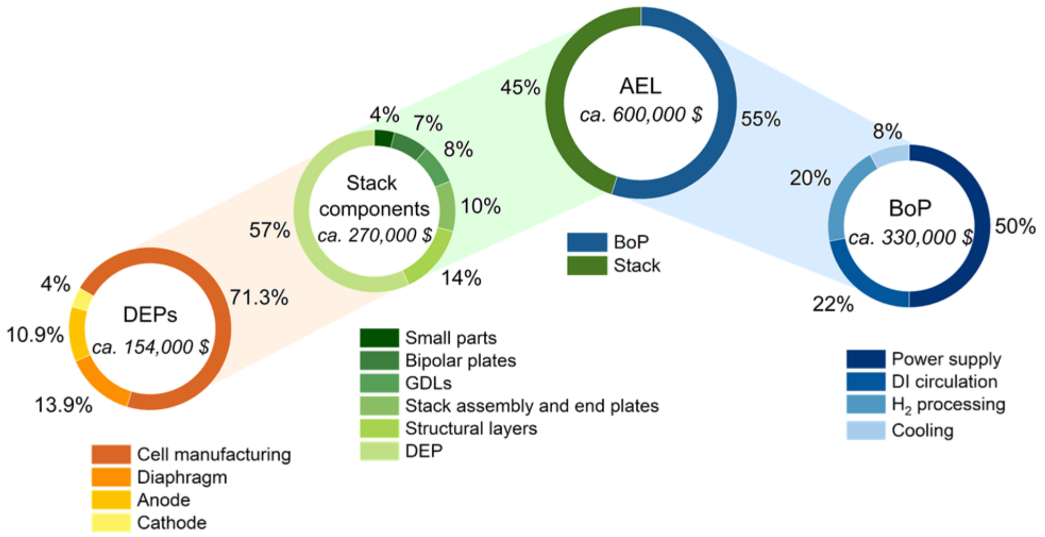

Figure S43. CAPEX breakdown for a generic 1 MW-scale AEL. Data retrieved from IRENA[15] and Lee *et al*.[16]

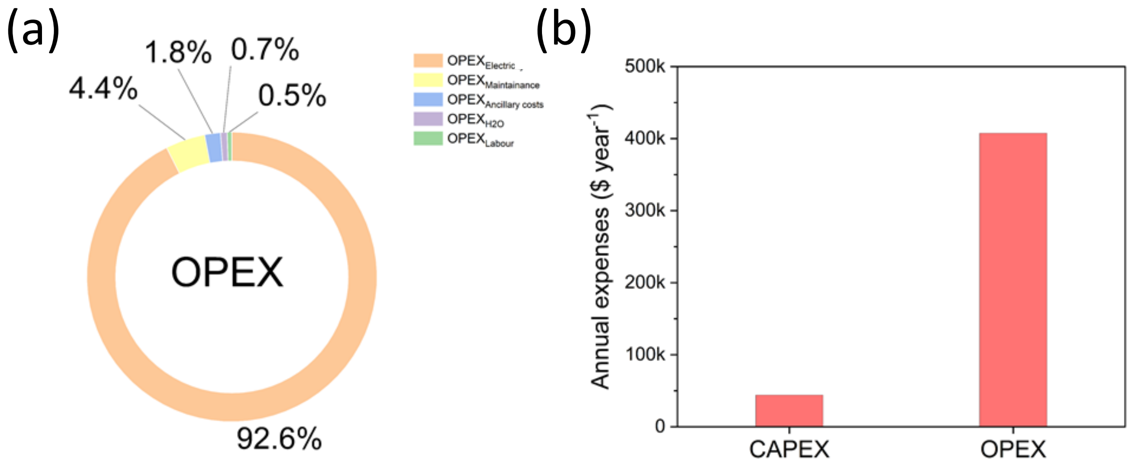

Figure S44. a, OPEX breakdown for an ideal 1 MW-scale AEL based on the DEP technology reported in this work; **b**, Comparison of annual CAPEX and OPEX. All data reported in the present figure are related to an AEL plant operating at 500 mA/cm^2 current density and for which energy efficiency calculations have been carried out using the H_2 HHV.

References

(1) Chen, R.; Yang, C.; Cai, W.; Wang, H. Y.; Miao, J.; Zhang, L.; Chen, S.; Liu, B. Use of Platinum as the Counter Electrode to Study the Activity of Nonprecious Metal Catalysts for the Hydrogen Evolution Reaction. *ACS Energy Lett.* **2017**, *2* (5), 1070–1075. https://doi.org/10.1021/acsenergylett.7b00219.

(2) Lee, J.; Bang, J. H. Reliable Counter Electrodes for the Hydrogen Evolution Reaction in Acidic Media. *ACS Energy Lett.* **2020**, *5* (8), 2706–2710. https://doi.org/10.1021/acsenergylett.0c01537.

(3) Najafi, L.; Oropesa-Nunez, R.; Bellani, S.; Martin-Garcia, B.; Pasquale, L.; Serri, M.; Drago, F.; Luxa, J.; Sofer, Z.; Sedmidubsky, D.; Brescia, R.; Lauciello, S.; Zappia, M. I.; Shinde, D. V.; Manna, L.; Bonaccorso, F. Topochemical Transformation of Two-Dimensional VSe2into Metallic Nonlayered VO2for Water Splitting Reactions in Acidic and Alkaline Media. *ACS Nano* **2022**, *16* (1), 351–367. https://doi.org/10.1021/acsnano.1c06662.

(4) Kweon, D. H.; Okyay, M. S.; Kim, S. J.; Jeon, J. P.; Noh, H. J.; Park, N.; Mahmood, J.; Baek, J. B. Ruthenium Anchored on Carbon Nanotube Electrocatalyst for Hydrogen Production with Enhanced Faradaic Efficiency. *Nat. Commun.* **2020**, *11* (1), 1278. https://doi.org/10.1038/s41467-020-15069-3.

(5) McCrory, C. C. L.; Jung, S.; Peters, J. C.; Jaramillo, T. F. Benchmarking Heterogeneous Electrocatalysts for the Oxygen Evolution Reaction. *J. Am. Chem. Soc.* **2013**, *135* (45), 16977–16987. https://doi.org/10.1021/ja407115p.

(6) Cicco, A. Di; Aquilanti, G.; Minicucci, M.; Principi, E.; Novello, N.; Cognigni, A.; Olivi, L. Novel XAFS Capabilities at ELETTRA Synchrotron Light Source. *J. Phys. Conf. Ser.* **2009**, *190*, 12043. https://doi.org/10.1088/1742-6596/190/1/012043.

(7) Ravel, B.; Newville, M. ATHENA, ARTEMIS, HEPHAESTUS: Data Analysis for X-Ray Absorption Spectroscopy Using IFEFFIT. *J. Synchrotron Radiat.* **2005**, *12* (4), 537–541. https://doi.org/10.1107/S0909049505012719.

(8) Kresse, G.; Furthmüller, J. Efficiency of Ab-Initio Total Energy Calculations for Metals and Semiconductors Using a Plane-Wave Basis Set. *Comput. Mater. Sci.* **1996**, *6* (1), 15–50. https://doi.org/10.1016/0927-0256(96)00008-0.

(9) Perdew, J. P.; Burke, K.; Ernzerhof, M. Generalized Gradient Approximation Made Simple. *Phys. Rev. Lett.* **1996**, *77* (18), 3865–3868. https://doi.org/10.1103/PhysRevLett.77.3865.

(10) Joubert, D. From Ultrasoft Pseudopotentials to the Projector Augmented-Wave Method. *Phys. Rev. B - Condens. Matter Mater. Phys.* **1999**, *59* (3), 1758–1775. https://doi.org/10.1103/PhysRevB.59.1758.

(11) Trinke, P.; Haug, P.; Brauns, J.; Bensmann, B.; Hanke-Rauschenbach, R.; Turek, T. Hydrogen Crossover in PEM and Alkaline Water Electrolysis: Mechanisms, Direct Comparison and Mitigation Strategies. *J. Electrochem. Soc.* **2018**, *165* (7), F502–F513. https://doi.org/10.1149/2.0541807jes.

(12) Brauns, J.; Turek, T. Experimental Evaluation of Dynamic Operating Concepts for Alkaline Water Electrolyzers Powered by Renewable Energy. *Electrochim. Acta* **2022**, *404*, 139715. https://doi.org/10.1016/j.electacta.2021.139715.

(13) Morozan, A.; Johnson, H.; Roiron, C.; Genay, G.; Aldakov, D.; Ghedjatti, A.; Nguyen, C. T.; Tran, P.

D.; Kinge, S.; Artero, V. Nonprecious Bimetallic Iron–molybdenum Sulfide Electrocatalysts for the Hydrogen Evolution Reaction in Proton Exchange Membrane Electrolyzers. *ACS Catal.* **2020**, *10* (24), 14336–14348. https://doi.org/10.1021/acscatal.0c03692.

(14) Lamy, C.; Millet, P. A Critical Review on the Definitions Used to Calculate the Energy Efficiency Coefficients of Water Electrolysis Cells Working under near Ambient Temperature Conditions. *J. Power Sources* **2020**, *447*, 227350. https://doi.org/10.1016/j.jpowsour.2019.227350.

(15) IRENA. Hydrogen Cost Reduction: Scaling Up Electrolysers to Meet the 1.5 OC Climate Goal. *Intenraional Renew. Energy Agency. Abu Dhabi.* **2020**, 105.

(16) Lee, B.; Cho, H. S.; Kim, H.; Lim, D.; Cho, W.; Kim, C. H.; Lim, H. Integrative Techno-Economic and Environmental Assessment for Green H2 Production by Alkaline Water Electrolysis Based on Experimental Data. *J. Environ. Chem. Eng.* **2021**, *9* (6), 106349. https://doi.org/10.1016/j.jece.2021.106349.

(17) Oswald, H. R.; Reller, A.; Schmalle, H. W.; Dubler, E. Structure of Copper(II) Hydroxide, Cu(OH)2. *Acta Crystallogr. Sect. C Cryst. Struct. Commun.* **1990**, *46* (12), 2279–2284. https://doi.org/10.1107/s0108270190006230.

(18) Member, A. C. A. *General , Atomic and Crystallographic Properties and Features of Copper*. https://www.copper.org/resources/properties/atomic_properties.html (accessed 2022-08-02).

(19) Shinde, D. V.; Dang, Z.; Petralanda, U.; Palei, M.; Wang, M.; Prato, M.; Cavalli, A.; De Trizio, L.; Manna, L. In Situ Dynamic Nanostructuring of the Cu-Ti Catalyst-Support System Promotes Hydrogen Evolution under Alkaline Conditions. *ACS Appl. Mater. Interfaces* **2018**, *10* (35), 29583–29592. https://doi.org/10.1021/acsami.8b09493.

(20) Huang, B.; Kobayashi, H.; Yamamoto, T.; Matsumura, S.; Nishida, Y.; Sato, K.; Nagaoka, K.; Kawaguchi, S.; Kubota, Y.; Kitagawa, H. Solid-Solution Alloying of Immiscible Ru and Cu with Enhanced CO Oxidation Activity. *J. Am. Chem. Soc.* **2017**, *139* (13), 4643–4646. https://doi.org/10.1021/jacs.7b01186.

(21) Ru (Ruthenium) Binary Alloy Phase Diagrams. *Alloy Phase Diagrams* **2018**, 579–581. https://doi.org/10.31399/asm.hb.v03.a0006200.

(22) Cu (Copper) Binary Alloy Phase Diagrams. *Alloy Phase Diagrams* **2018**, 304–326. https://doi.org/10.31399/asm.hb.v03.a0006159.

(23) Massalski, E. T. B.; Okamoto, H.; Subramanian, P.; Kacprzak, L. *Binary alloy phase diagrams Second Edition. ASM International*. https://www.asminternational.org/binary-alloy-phase-diagrams-2nd-edition/results/-/journal_content/56/57718G/PUBLICATION/ (accessed 2023-08-10).

(24) Chyan, O.; Arunagiri, T. N.; Ponnuswamy, T. Electrodeposition of Copper Thin Film on Ruthenium. *J. Electrochem. Soc.* **2003**, *150* (5), C347. https://doi.org/10.1149/1.1565138.

(25) Wu, Q.; Luo, M.; Han, J.; Peng, W.; Zhao, Y.; Chen, D.; Peng, M.; Liu, J.; De Groot, F. M. F.; Tan, Y. Identifying Electrocatalytic Sites of the Nanoporous Copper-Ruthenium Alloy for Hydrogen Evolution Reaction in Alkaline Electrolyte. *ACS Energy Lett.* **2020**, *5* (1), 192–199. https://doi.org/10.1021/acsenergylett.9b02374.

(26) Kusada, K.; Kobayashi, H.; Yamamoto, T.; Matsumura, S.; Sumi, N.; Sato, K.; Nagaoka, K.; Kubota, Y.; Kitagawa, H. Discovery of Face-Centered-Cubic Ruthenium Nanoparticles: Facile Size-Controlled

Synthesis Using the Chemical Reduction Method. *J. Am. Chem. Soc.* **2013**, *135* (15), 5493–5496. https://doi.org/10.1021/ja311261s.

(27) Wu, Q.; Luo, M.; Han, J.; Peng, W.; Zhao, Y.; Chen, D.; Peng, M.; Liu, J.; De Groot, F. M. F.; Tan, Y. Identifying Electrocatalytic Sites of the Nanoporous Copper-Ruthenium Alloy for Hydrogen Evolution Reaction in Alkaline Electrolyte. *ACS Energy Lett.* **2020**, *5* (1), 192–199. https://doi.org/10.1021/acsenergylett.9b02374.

(28) Chen, F. Y.; Wu, Z. Y.; Gupta, S.; Rivera, D. J.; Lambeets, S. V.; Pecaut, S.; Kim, J. Y. T.; Zhu, P.; Finfrock, Y. Z.; Meira, D. M.; King, G.; Gao, G.; Xu, W.; Cullen, D. A.; Zhou, H.; Han, Y.; Perea, D. E.; Muhich, C. L.; Wang, H. Efficient Conversion of Low-Concentration Nitrate Sources into Ammonia on a Ru-Dispersed Cu Nanowire Electrocatalyst. *Nat. Nanotechnol.* **2022**, *17* (7), 759–767. https://doi.org/10.1038/s41565-022-01121-4.

(29) Huang, H.; Jung, H.; Li, S.; Kim, S.; Han, J. W.; Lee, J. Activation of Inert Copper for Significantly Enhanced Hydrogen Evolution Behaviors by Trace Ruthenium Doping. *Nano Energy* **2022**, *92*, 106763. https://doi.org/10.1016/j.nanoen.2021.106763.

(30) Yang, Y.; Yang, F.; Sun, C. J.; Zhao, H.; Hao, S.; Brown, D. E.; Zhang, J.; Ren, Y. Ru-Fe Alloy Mediated α-Fe2O3 Particles on Mesoporous Carbon Nanofibers as Electrode Materials with Superior Capacitive Performance. *RSC Adv.* **2017**, *7* (12), 6818–6826. https://doi.org/10.1039/c6ra27324f.

(31) Huang, B.; Kobayashi, H.; Yamamoto, T.; Matsumura, S.; Nishida, Y.; Sato, K.; Nagaoka, K.; Kawaguchi, S.; Kubota, Y.; Kitagawa, H. Solid-Solution Alloying of Immiscible Ru and Cu with Enhanced CO Oxidation Activity. *J. Am. Chem. Soc.* **2017**, *139* (13), 4643–4646. https://doi.org/10.1021/jacs.7b01186.

(32) Biesinger, M. C. *X-Ray Photoelectron Spectroscopy (XPS) Reference Pages. Titanium.* http://www.xpsfitting.com/2008/09/titanium.html%0Awww.xpsfitting.com/search/label/Titanium (accessed 2022-07-26).

(33) *Alkaline Water Electrolyzer System(1Kw) User Manual*. https://www.fuelcellstore.com/alkaline-water-electrolyzer-stack-20-cell?search=alkaline water ele (accessed 2023-08-10).

(34) Dinh, C. T.; Jain, A.; de Arquer, F. P. G.; De Luna, P.; Li, J.; Wang, N.; Zheng, X.; Cai, J.; Gregory, B. Z.; Voznyy, O.; Zhang, B.; Liu, M.; Sinton, D.; Crumlin, E. J.; Sargent, E. H. Multi-Site Electrocatalysts for Hydrogen Evolution in Neutral Media by Destabilization of Water Molecules. *Nat. Energy* **2019**, *4* (2), 107–114. https://doi.org/10.1038/s41560-018-0296-8.

(35) Jiang, K.; Liu, B.; Luo, M.; Ning, S.; Peng, M.; Zhao, Y.; Lu, Y. R.; Chan, T. S.; de Groot, F. M. F.; Tan, Y. Single Platinum Atoms Embedded in Nanoporous Cobalt Selenide as Electrocatalyst for Accelerating Hydrogen Evolution Reaction. *Nat. Commun.* **2019**, *10* (1), 1–9. https://doi.org/10.1038/s41467-019-09765-y.

(36) Wu, R.; Xiao, B.; Gao, Q.; Zheng, Y.; Zheng, X.; Zhu, J.; Gao, M.; Yu, S. A Janus Nickel Cobalt Phosphide Catalyst for High-Efficiency Neutral-pH Water Splitting. *Angew. Chemie* **2018**, *130* (47), 15671–15675. https://doi.org/10.1002/ange.201808929.

(37) Zhang, L.; Han, L.; Liu, H.; Liu, X.; Luo, J. Potential-Cycling Synthesis of Single Platinum Atoms for Efficient Hydrogen Evolution in Neutral Media. *Angew. Chemie - Int. Ed.* **2017**, *56* (44), 13694–13698. https://doi.org/10.1002/anie.201706921.

(38) Gao, X.; Chen, Y.; Sun, T.; Huang, J.; Zhang, W.; Wang, Q.; Cao, R. Karst Landform-Featured

Monolithic Electrode for Water Electrolysis in Neutral Media. *Energy Environ. Sci.* **2020**, *13* (1), 174–182. https://doi.org/10.1039/c9ee02380a.

(39) Li, C.; Liu, M.; Ding, H.; He, L.; Wang, E.; Wang, B.; Fan, S.; Liu, K. A Lightly Fe-Doped (NiS2/MoS2)/Carbon Nanotube Hybrid Electrocatalyst Film with Laser-Drilled Micropores for Stabilized Overall Water Splitting and PH-Universal Hydrogen Evolution Reaction. *J. Mater. Chem. A* **2020**, *8* (34), 17527–17536. https://doi.org/10.1039/d0ta04586a.

(40) Wang, Y.; Chen, L.; Yu, X.; Wang, Y.; Zheng, G. Superb Alkaline Hydrogen Evolution and Simultaneous Electricity Generation by Pt-Decorated Ni3N Nanosheets. *Adv. Energy Mater.* **2017**, *7* (2), 1601390. https://doi.org/10.1002/aenm.201601390.

(41) Anantharaj, S.; Noda, S.; Driess, M.; Menezes, P. W. The Pitfalls of Using Potentiodynamic Polarization Curves for Tafel Analysis in Electrocatalytic Water Splitting. *ACS Energy Lett.* **2021**, *6* (4), 1607–1611. https://doi.org/10.1021/acsenergylett.1c00608.

(42) Tian, X.; Zhao, P.; Sheng, W. Hydrogen Evolution and Oxidation: Mechanistic Studies and Material Advances. *Adv. Mater.* **2019**, *31* (31), 1808066. https://doi.org/10.1002/adma.201808066.

(43) Xie, C.; Chen, W.; Du, S.; Yan, D.; Zhang, Y.; Chen, J.; Liu, B.; Wang, S. In-Situ Phase Transition of WO3 Boosting Electron and Hydrogen Transfer for Enhancing Hydrogen Evolution on Pt. *Nano Energy* **2020**, *71*, 104653. https://doi.org/10.1016/j.nanoen.2020.104653.

(44) Chen, W.; Wu, B.; Wang, Y.; Zhou, W.; Li, Y.; Liu, T.; Xie, C.; Xu, L.; Du, S.; Song, M.; Wang, D.; Liu, Y.; Li, Y.; Liu, J.; Zou, Y.; Chen, R.; Chen, C.; Zheng, J.; Li, Y.; Chen, J.; Wang, S. Deciphering the Alternating Synergy between Interlayer Pt Single-Atom and NiFe Layered Double Hydroxide for Overall Water Splitting. *Energy Environ. Sci.* **2021**, *14* (12), 6428–6440. https://doi.org/10.1039/d1ee01395e.

(45) Chen, Y.; Ren, Z.; Fu, H.; Zhang, X.; Tian, G.; Fu, H. NiSe-Ni0.85Se Heterostructure Nanoflake Arrays on Carbon Paper as Efficient Electrocatalysts for Overall Water Splitting. *Small* **2018**, *14* (25), 1800763. https://doi.org/10.1002/smll.201800763.

(46) Hu, Z.; Zhang, L.; Huang, J.; Feng, Z.; Xiong, Q.; Ye, Z.; Chen, Z.; Li, X.; Yu, Z. Self-Supported Nickel-Doped Molybdenum Carbide Nanoflower Clusters on Carbon Fiber Paper for an Efficient Hydrogen Evolution Reaction. *Nanoscale* **2021**, *13* (17), 8264–8274. https://doi.org/10.1039/d1nr00169h.

(47) Trasatti, S.; Petrii, O. A. Real Surface Area Measurements in Electrochemistry. *J. Electroanal. Chem.* **1992**, *327* (1–2), 353–376. https://doi.org/10.1016/0022-0728(92)80162-W.

(48) Zappia, M. I.; Mastronardi, V.; Bellani, S.; Zuo, Y.; Bianca, G.; Gabatel, L.; Gentile, M.; Bagheri, A.; Beydaghi, H.; Drago, F.; Ferri, M.; Moglianetti, M.; Pompa, P. P.; Manna, L.; Bonaccorso, F. Graphene vs. Carbon Black Supports for Pt Nanoparticles: Towards next-Generation Cathodes for Advanced Alkaline Electrolyzers. *Electrochim. Acta* **2023**, *462*, 142696. https://doi.org/10.1016/j.electacta.2023.142696.

(49) Łosiewicz, B.; Martin, M.; Lebouin, C.; Lasia, A. Kinetics of Hydrogen Underpotential Deposition at Ruthenium in Acidic Solutions. *J. Electroanal. Chem.* **2010**, *649* (1–2), 198–205. https://doi.org/10.1016/j.jelechem.2010.04.002.

(50) Lin, G.; Ju, Q.; Liu, L.; Guo, X.; Zhu, Y.; Zhang, Z.; Zhao, C.; Wan, Y.; Yang, M.; Huang, F.; Wang, J. Caged-Cation-Induced Lattice Distortion in Bronze TiO 2 for Cohering Nanoparticulate Hydrogen Evolution Electrocatalysts . *ACS Nano* **2022**. https://doi.org/10.1021/acsnano.2c04513.

(51) Najafi, L.; Oropesa-Nunez, R.; Bellani, S.; Martin-Garcia, B.; Pasquale, L.; Serri, M.; Drago, F.; Luxa, J.; Sofer, Z.; Sedmidubsky, D.; Brescia, R.; Lauciello, S.; Zappia, M. I.; Shinde, D. V.; Manna, L.; Bonaccorso, F. Topochemical Transformation of Two-Dimensional VSe2into Metallic Nonlayered VO2for Water Splitting Reactions in Acidic and Alkaline Media. *ACS Nano* **2022**, *16* (1), 351–367. https://doi.org/10.1021/acsnano.1c06662.

(52) Fletcher, S. Tafel Slopes from First Principles. *J. Solid State Electrochem.* **2009**, *13* (4), 537–549. https://doi.org/10.1007/s10008-008-0670-8.

(53) Hu, Q.; Gao, K.; Wang, X.; Zheng, H.; Cao, J.; Mi, L.; Huo, Q.; Yang, H.; Liu, J.; He, C. Subnanometric Ru Clusters with Upshifted D Band Center Improve Performance for Alkaline Hydrogen Evolution Reaction. *Nat. Commun.* **2022**, *13* (1), 1–10. https://doi.org/10.1038/s41467-022-31660-2.

(54) Zhao, Y.; Kumar, P. V.; Tan, X.; Lu, X.; Zhu, X.; Jiang, J.; Pan, J.; Xi, S.; Yang, H. Y.; Ma, Z.; Wan, T.; Chu, D.; Jiang, W.; Smith, S. C.; Amal, R.; Han, Z.; Lu, X. Modulating Pt-O-Pt Atomic Clusters with Isolated Cobalt Atoms for Enhanced Hydrogen Evolution Catalysis. *Nat. Commun.* **2022**, *13* (1), 2430. https://doi.org/10.1038/s41467-022-30155-4.

(55) Fan, Z.; Liao, F.; Ji, Y.; Liu, Y.; Huang, H.; Wang, D.; Yin, K.; Yang, H.; Ma, M.; Zhu, W.; Wang, M.; Kang, Z.; Li, Y.; Shao, M.; Hu, Z.; Shao, Q. Coupling of Nanocrystal Hexagonal Array and Two-Dimensional Metastable Substrate Boosts H2-Production. *Nat. Commun.* **2022**, *13* (1), 5828. https://doi.org/10.1038/s41467-022-33512-5.

(56) Li, G.; Jang, H.; Liu, S.; Li, Z.; Kim, M. G.; Qin, Q.; Liu, X.; Cho, J. The Synergistic Effect of Hf-O-Ru Bonds and Oxygen Vacancies in Ru/HfO2 for Enhanced Hydrogen Evolution. *Nat. Commun.* **2022**, *13* (1), 1270. https://doi.org/10.1038/s41467-022-28947-9.

(57) Zhu, L.; Lin, H.; Li, Y.; Liao, F.; Lifshitz, Y.; Sheng, M.; Lee, S. T.; Shao, M. A Rhodium/Silicon Co-Electrocatalyst Design Concept to Surpass Platinum Hydrogen Evolution Activity at High Overpotentials. *Nat. Commun.* **2016**, *7* (1), 12272. https://doi.org/10.1038/ncomms12272.

(58) Shinagawa, T.; Garcia-Esparza, A. T.; Takanabe, K. Insight on Tafel Slopes from a Microkinetic Analysis of Aqueous Electrocatalysis for Energy Conversion. *Sci. Rep.* **2015**, *5* (1), 13805. https://doi.org/10.1038/srep13801.

(59) Anantharaj, S.; Kundu, S. Do the Evaluation Parameters Reflect Intrinsic Activity of Electrocatalysts in Electrochemical Water Splitting? *ACS Energy Lett.* **2019**, *4* (6), 1260–1264. https://doi.org/10.1021/acsenergylett.9b00686.

(60) Anantharaj, S.; Karthik, P. E.; Noda, S. The Significance of Properly Reporting Turnover Frequency in Electrocatalysis Research. *Angew. Chemie - Int. Ed.* **2021**, *60* (43), 23051–23067. https://doi.org/10.1002/anie.202110352.

(61) McCrum, I. T.; Koper, M. T. M. The Role of Adsorbed Hydroxide in Hydrogen Evolution Reaction Kinetics on Modified Platinum. *Nat. Energy* **2020**, *5* (11), 891–899. https://doi.org/10.1038/s41560-020-00710-8.

(62) Ledezma-Yanez, I.; Wallace, W. D. Z.; Sebastián-Pascual, P.; Climent, V.; Feliu, J. M.; Koper, M. T. M. Interfacial Water Reorganization as a PH-Dependent Descriptor of the Hydrogen Evolution Rate on Platinum Electrodes. *Nat. Energy* **2017**, *2* (4), 17031. https://doi.org/10.1038/nenergy.2017.31.

(63) Chen, W.; Wu, B.; Wang, Y.; Zhou, W.; Li, Y.; Liu, T.; Xie, C.; Xu, L.; Du, S.; Song, M.; Wang, D.; Liu, Y.; Li, Y.; Liu, J.; Zou, Y.; Chen, R.; Chen, C.; Zheng, J.; Li, Y.; Chen, J.; Wang, S. Deciphering the

Alternating Synergy between Interlayer Pt Single-Atom and NiFe Layered Double Hydroxide for Overall Water Splitting. *Energy Environ. Sci.* **2021**, *14* (12), 6428–6440. https://doi.org/10.1039/d1ee01395e.

(64) Guidelli, R.; Compton, R. G.; Feliu, J. M.; Gileadi, E.; Lipkowski, J.; Schmickler, W.; Trasatti, S. Defining the Transfer Coefficient in Electrochemistry: An Assessment (IUPAC Technical Report). *Pure Appl. Chem.* **2014**, *86* (2), 245–258. https://doi.org/10.1515/pac-2014-5026.

(65) Antipin, D.; Risch, M. Calculation of the Tafel Slope and Reaction Order of the Oxygen Evolution Reaction between PH 12 and PH 14 for the Adsorbate Mechanism. *chemrxiv* **2021**. https://doi.org/10.26434/CHEMRXIV-2021-HGBQ6.

(66) Zhu, L.; Lin, H.; Li, Y.; Liao, F.; Lifshitz, Y.; Sheng, M.; Lee, S. T.; Shao, M. A Rhodium/Silicon Co-Electrocatalyst Design Concept to Surpass Platinum Hydrogen Evolution Activity at High Overpotentials. *Nat. Commun.* **2016**, *7* (1), 12272. https://doi.org/10.1038/ncomms12272.

(67) Li, D.; Batchelor-McAuley, C.; Compton, R. G. Some Thoughts about Reporting the Electrocatalytic Performance of Nanomaterials. *Appl. Mater. Today* **2020**, *18*, 100404. https://doi.org/10.1016/j.apmt.2019.05.011.

(68) Li, D.; Lin, C.; Batchelor-McAuley, C.; Chen, L.; Compton, R. G. Tafel Analysis in Practice. *J. Electroanal. Chem.* **2018**, *826*, 117–124. https://doi.org/10.1016/j.jelechem.2018.08.018.

(69) Shiddiky, M. J. A.; O'Mullane, A. P.; Zhang, J.; Burke, L. D.; Bond, A. M. Large Amplitude Fourier Transformed AC Voltammetric Investigation of the Active State Electrochemistry of a Copper/Aqueous Base Interface and Implications for Electrocatalysis. *Langmuir* **2011**, *27* (16), 10302–10311. https://doi.org/10.1021/la2017819.

(70) Härtinger, S.; Pettinger, B.; Doblhofer, K. Cathodic Formation of a Hydroxyde Adsorbate on Copper (111) Electrodes in Alkaline Electrolyte. *J. Electroanal. Chem.* **1995**, *397* (1–2), 335–338. https://doi.org/10.1016/0022-0728(95)04297-5.

(71) Chen, R.; Yang, C.; Cai, W.; Wang, H. Y.; Miao, J.; Zhang, L.; Chen, S.; Liu, B. Use of Platinum as the Counter Electrode to Study the Activity of Nonprecious Metal Catalysts for the Hydrogen Evolution Reaction. *ACS Energy Lett.* **2017**, *2* (5), 1070–1075. https://doi.org/10.1021/acsenergylett.7b00219.

(72) Zhang, J.; Zhang, L.; Liu, J.; Zhong, C.; Tu, Y.; Li, P.; Du, L.; Chen, S.; Cui, Z. OH Spectator at IrMo Intermetallic Narrowing Activity Gap between Alkaline and Acidic Hydrogen Evolution Reaction. *Nat. Commun.* **2022**, *13* (1), 5497. https://doi.org/10.1038/s41467-022-33216-w.

(73) Li, C.; Wang, Z.; Liu, M.; Wang, E.; Wang, B.; Xu, L.; Jiang, K.; Fan, S.; Sun, Y.; Li, J.; Liu, K. Ultrafast Self-Heating Synthesis of Robust Heterogeneous Nanocarbides for High Current Density Hydrogen Evolution Reaction. *Nat. Commun.* **2022**, *13* (1), 3338. https://doi.org/10.1038/s41467-022-31077-x.

(74) Tian, M.; Cousins, C.; Beauchemin, D.; Furuya, Y.; Ohma, A.; Jerkiewicz, G. Influence of the Working and Counter Electrode Surface Area Ratios on the Dissolution of Platinum under Electrochemical Conditions. *ACS Catal.* **2016**, *6* (8), 5108–5116. https://doi.org/10.1021/acscatal.6b00200.

(75) Cui, Z.; Sheng, W. Thoughts about Choosing a Proper Counter Electrode. *ACS Catal.* **2023**, *13* (4), 2534–2541. https://doi.org/10.1021/acscatal.2c05145.

(76) Nørskov, J. K.; Bligaard, T.; Logadottir, A.; Kitchin, J. R.; Chen, J. G.; Pandelov, S.; Stimming, U. Trends in the Exchange Current for Hydrogen Evolution. *J. Electrochem. Soc.* **2005**, *152* (3), J23. https://doi.org/10.1149/1.1856988/XML.

(77) Alexander, C. S.; Pritchard, J. Chemisorption of Hydrogen on Evaporated Copper Films. *J. Chem. Soc. Faraday Trans. 1 Phys. Chem. Condens. Phases* **1972**, *68* (0), 202–215. https://doi.org/10.1039/F19726800202.

(78) Pliego, J. R.; Riveros, J. M. New Values for the Absolute Solvation Free Energy of Univalent Ions in Aqueous Solution. *Chem. Phys. Lett.* **2000**, *332* (5–6), 597–602. https://doi.org/10.1016/S0009-2614(00)01305-1.

(79) Sun, W.; Ceder, G. Efficient Creation and Convergence of Surface Slabs. *Surf. Sci.* **2013**, *617*, 53–59. https://doi.org/10.1016/j.susc.2013.05.016.

(80) Matta, C. F.; Boyd, R. J. The Quantum Theory of Atoms in Molecules: From Solid State to DNA and Drug Design. *Quantum Theory Atoms Mol. From Solid State to DNA Drug Des.* **2007**, 1–527. https://doi.org/10.1002/9783527610709.

(81) Vanpoucke, D. E. P.; Bultinck, P.; Van Driessche, I. Extending Hirshfeld-I to Bulk and Periodic Materials. *J. Comput. Chem.* **2013**, *34* (5), 405–417. https://doi.org/10.1002/jcc.23088.

(82) Houston, J. E.; Peden, C. H. F.; Blair, D. S.; Goodman, D. W. Monolayer and Multilayer Growth of Cu on the Ru(0001) Surface. *Surf. Sci.* **1986**, *167* (2–3), 427–436. https://doi.org/10.1016/0039-6028(86)90715-6.

(83) de Groot, M. T.; Vreman, A. W. Ohmic Resistance in Zero Gap Alkaline Electrolysis with a Zirfon Diaphragm. *Electrochim. Acta* **2021**, *369*, 137684. https://doi.org/10.1016/j.electacta.2020.137684.

(84) Zhu, Y.; Tahini, H. A.; Hu, Z.; Dai, J.; Chen, Y.; Sun, H.; Zhou, W.; Liu, M.; Smith, S. C.; Wang, H.; Shao, Z. Unusual Synergistic Effect in Layered Ruddlesden−Popper Oxide Enables Ultrafast Hydrogen Evolution. *Nat. Commun.* **2019**, *10* (1), 149. https://doi.org/10.1038/s41467-018-08117-6.

(85) *Raney Ni-Raney Ni Manufacturers, Suppliers and Exporters on Alibaba.comOther Game Accessories*. https://www.alibaba.com/trade/search?spm=a2700.product_home_newuser.the-new-header_fy23_pc_search_bar.keydown__Enter&tab=all&searchText=Raney+Ni (accessed 2023-08-10).

(86) Zappia, M. I.; Bellani, S.; Zuo, Y.; Ferri, M.; Drago, F.; Manna, L.; Bonaccorso, F. High-Current Density Alkaline Electrolyzers: The Role of Nafion Binder Content in the Catalyst Coatings and Techno-Economic Analysis. *Front. Chem.* **2022**, *10,* 1045212. https://doi.org/10.3389/FCHEM.2022.1045212.

(87) Buttler, A.; Spliethoff, H. Current Status of Water Electrolysis for Energy Storage, Grid Balancing and Sector Coupling via Power-to-Gas and Power-to-Liquids: A Review. *Renew. Sustain. Energy Rev.* **2018**, *82*, 2440–2454. https://doi.org/10.1016/j.rser.2017.09.003.

(88) Yates, J.; Daiyan, R.; Patterson, R.; Egan, R.; Amal, R.; Ho-Baille, A.; Chang, N. L. Techno-Economic Analysis of Hydrogen Electrolysis from Off-Grid Stand-Alone Photovoltaics Incorporating Uncertainty Analysis. *Cell Reports Phys. Sci.* **2020**, *1* (10), 100209. https://doi.org/10.1016/j.xcrp.2020.100209.
